# The primate gut mycobiome-bacteriome interface is impacted by environmental and subsistence factors

**DOI:** 10.1038/s41522-022-00274-3

**Published:** 2022-03-17

**Authors:** Ashok K. Sharma, Sam Davison, Barbora Pafco, Jonathan B. Clayton, Jessica M. Rothman, Matthew R. McLennan, Marie Cibot, Terence Fuh, Roman Vodicka, Carolyn Jost Robinson, Klara Petrzelkova, Andres Gomez

**Affiliations:** 1 Department of Animal Science, University of Minnesota, St. Paul, MN USA; 2 Institute of Vertebrate Biology, Czech Academy of Sciences, Brno, Czech Republic; 3 Department of Biology, University of Nebraska at Omaha, Omaha, NE USA; 4 Callitrichid Research Center (CRC, Marmoset Colony) at the University of Nebraska at Omaha, Omaha, NE USA; 5 Department of Food Science and Technology, University of Nebraska-Lincoln, Lincoln, NE USA; 6 Primate Microbiome Project, University of Nebraska-Lincoln, Lincoln, NE USA; 7 Department of Anthropology, Hunter College of the City University of New York, 695 Park Avenue, New York, NY USA; 8 New York Consortium in Evolutionary Primatology, New York, NY USA; 9 Department of Social Sciences, Faculty of Humanities and Social Sciences, Oxford Brookes University, Oxford, UK; 10 Bulindi Chimpanzee & Community Project, Hoima, Uganda; 11 Anicoon Vétérinaires, Ploemeur, France; 12 WWF Central African Republic, Bayanga, Central African Republic; 13 Prague Zoo, Prague, Czech Republic; 14 Department of Anthropology, Purdue University, West Lafayette, IN USA; 15 Institute of Parasitology, Biology Centre, Czech Academy of Sciences, Ceske Budejovice, Brno, Czech Republic; 16 Department of Food Science and Nutrition, University of Minnesota, St. Paul, MN USA; 17 Present Address: Department of Gastroenterology, Inflammatory Bowel & Immunology Research Institute, Cedars Sinai Medical Center, Los Angeles, CA USA

**Keywords:** Microbial ecology, Symbiosis, Microbiome

## Abstract

The gut microbiome of primates is known to be influenced by both host genetic background and subsistence strategy. However, these inferences have been made mainly based on adaptations in bacterial composition - the bacteriome and have commonly overlooked the fungal fraction - the mycobiome. To further understand the factors that shape the gut mycobiome of primates and mycobiome-bacteriome interactions, we sequenced 16 S rRNA and ITS2 markers in fecal samples of four different nonhuman primate species and three human groups under different subsistence patterns (*n* = 149). The results show that gut mycobiome composition in primates is still largely unknown but highly plastic and weakly structured by primate phylogeny, compared with the bacteriome. We find significant gut mycobiome overlap between captive apes and human populations living under industrialized subsistence contexts; this is in contrast with contemporary hunter-gatherers and agriculturalists, who share more mycobiome traits with diverse wild-ranging nonhuman primates. In addition, mycobiome-bacteriome interactions were specific to each population, revealing that individual, lifestyle and intrinsic ecological factors affect structural correspondence, number, and kind of interactions between gut bacteria and fungi in primates. Our findings indicate a dominant effect of ecological niche, environmental factors, and diet over the phylogenetic background of the host, in shaping gut mycobiome composition and mycobiome-bacteriome interactions in primates.

## Introduction

The gut mycobiome, the communities of fungal species that colonize the gastrointestinal tract of humans and animals, still constitutes a poorly explored fraction of the gut microbiome. These neglected microbial communities have received increasing attention, as they have been recently associated with roles in regulating host immune responses and various chronic gastrointestinal diseases^1–4^. Bacterial-fungal interactions, either through direct contact or exchange of small molecules, have also been reported to have clinical importance at various anatomical sites^5–9^. Recently, mice models have been used to show that the gut mycobiome is strongly influenced by environment and diet, including potential influences on mice metabolic phenotypes^10^. However, our understanding of how gut fungal communities adapt and/or interact with bacterial communities in the context of lifestyles and diet, is still limited.

In recent years, the study of nonhuman primate microbiomes has offered valuable insights for understanding the evolutionary and ecological factors shaping the human microbiome. Although most of the work in this research area has concentrated on the gut bacteriome, some reports have investigated other microbial kingdoms in the nonhuman primate gut^11–13^. These reports highlight positive correlations between eukaryotic and bacterial diversity, weakly structured biodiversity of gut eukaryotes according to primate phylogeny, unshared dominant fungal taxa across different nonhuman primates, and involvement of anaerobic fungi in the degradation of complex polysaccharides, further facilitating bacterial fermentation in the gut^13^. Influence of host phylogeny, captivity, fermentation strategy, and dietary fiber content on the composition of different anaerobic fungi has been reported in primates^14–16^. However, our understanding of the relationship between mycobiome and bacteriome, in the context of phylosymbiosis, host ecology and subsistence across diverse members of the primate order, including humans, is still limited.

In the present study, we produced ITS2 and 16 S rRNA MiSeq data in 52 fecal samples collected from three human groups characterized by different subsistence strategies (urban or industrialized, traditional agriculture and hunting-gathering), and compared them with data obtained in 97 samples from seven nonhuman primate populations; composed of wild and captive apes (chimpanzees and gorillas), and the distantly related wild agile mangabeys. By investigating this multi-kingdom and multi-host comparison, we intend to shed light on the phylogenetic and ecological factors shaping fungal diversity in the primate gut, and on how the interface between the mycobiome and bacteriome is impacted by these factors.

## Results

After collecting fecal samples of western lowland gorillas (*Gorilla gorilla gorilla*, *n* = 19), agile mangabeys (*Cercocebus agilis*, *n* = 11), BaAka hunter-gatherers (*n* = 27), Bantu agriculturalists (*n* = 13), eastern chimpanzees (*Pan troglodytes schweinfurthii*, *n* = 11), captive western lowland gorillas (*n* = 18), captive chimpanzees (*n* = 12), mountain gorillas (*Gorilla beringei beringei*, *n* = 26), and a US human cohort (*n* = 12), we profiled gut bacteriome and mycobiome composition via 16 S rRNA and ITS2 MiSeq sequencing.

### Distinct mycobiome composition in phylogenetically similar primates

**Fig. 1:**
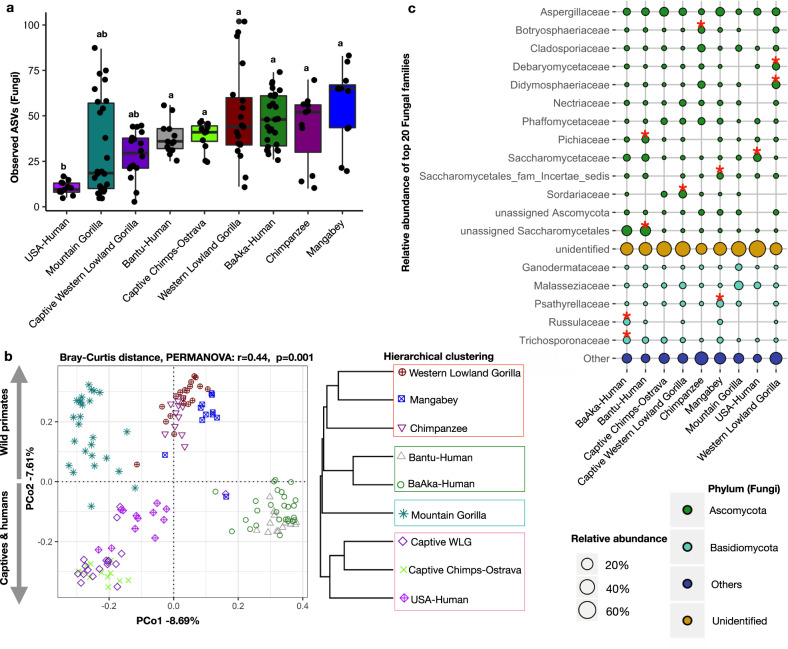
Gut mycobiome composition differs based on subsistence strategy across different primates. **a** Alpha diversity analysis showing lower fungal richness in US humans, compared with other primate populations. **b** Principal coordinate analysis based on Bray-Curtis distances showing different fungal community composition across different primate groups. Each symbol represents mycobiome composition, at the ASV level, in the fecal samples of an individual primate. A Bray-Curtis distance dendrogram (hierarchical clustering, average distances) based on average fungal ASV abundances showed similarities between phylogenetically distinct primate groups. Each group with similar mycobiome composition is shown to reflect PAM clustering (Supplementary Fig. 2). **c** Relative abundance of top 20 fungal families and their mean distribution among different primate groups are shown in a Bubble plot. Color code represents membership of each family to a specific Fungal Phylum. Differentially abundant taxa (Supplementary Table 1) were identified using Indicator Species Analysis and the families marked with stars are representative of a given primate group. The identifiers used for primate groups are Western Lowland Gorilla: WLG, Mangabey: Agile Mangabeys and Chimps: Chimpanzee. In boxplots, center values indicate the median; bounds of box represents lower/upper quartiles; whiskers show inner fences.

We obtained 1,530,381 ITS2 and 3,688,979 16 S rRNA filtered reads, respectively, with an average sequencing depth of 10,271/sample and 24,758/sample for mycobiome and bacteriome, respectively. Mycobiome diversity was highly variable in individuals of each sampled group, but not significantly different across most primate groups studied; except for US humans, who showed the lowest fungal richness and diversity (Fig. 1a, and Supplementary Fig. 1). Principal coordinates analysis (PCoA) based on Bray-Curtis distances on the relative abundances of amplicon sequence variants (ASVs) showed significant stratification (PERMANOVA, *R*2 = 0.44 and *P* < 0.001) in the gut mycobiome composition of all groups studied (Fig. 1b). Hierarchical clustering analysis on cumulative relative abundances of each population (average distance method) showed the presence of two main clusters, one composed of US humans and all captive apes and another one composed of all wild primates and the traditional human populations (Fig. 1b right panel). To further validate these grouping patterns, an unsupervised clustering analysis led to the identification of four specific clusters (K means clustering Supplementary Fig. 2a); composed of mountain gorillas (Cluster1); US humans and all captive apes (captive chimpanzee and captive western lowland gorillas) (Cluster2); all wild primates (wild chimpanzees, wild agile mangabeys and wild western lowland gorillas) (Cluster3); and both traditional human groups (BaAka hunter-gatherers and Bantu agriculturalists) (Cluster4) (Supplementary Fig. 2b, c). These ecologically driven clustering patterns further highlight the dominant role of subsistence strategies over host phylogeny in shaping the primate gut mycobiome.

### Specific taxonomic signatures distinguish primate populations and ecological clusters

Taxonomic assessment of the most abundant fungal families led to the identification of Ascomycota and Basidiomycota as the main fungal phyla in the primate gut (Fig. 1c and Supplementary Fig. 3). However, an analysis of representative fungal families indicates that the majority of ITS2 sequences generated in all samples could not be assigned to any taxonomic group (unidentified sequences, Fig. 1c), indicating that, in general, the primate gut mycobiome, including that of humans, is largely unknown. Regardless, we identified some families that were characteristic of specific primate populations (indicator species analysis, indicator value > 0.3, *p* < 0.05, Fig. 1c, Supplementary Table 1).

**Fig. 2:**
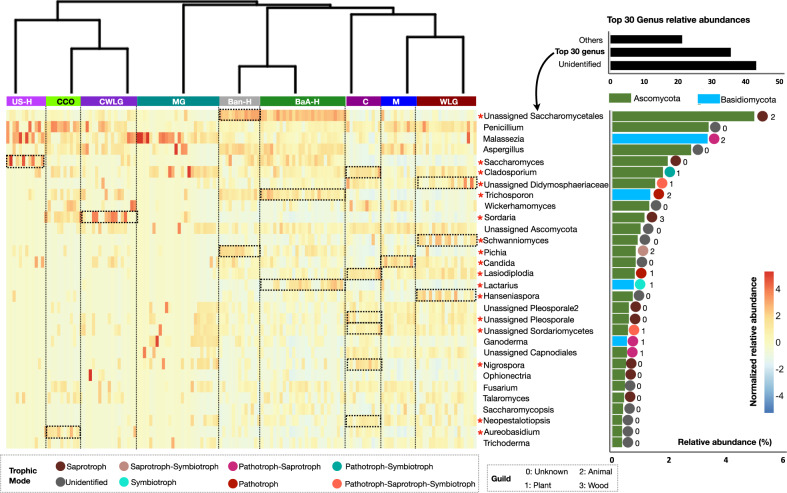
Heatmap distribution of 30 most abundant fungal genera. Heatmap showing the relative distribution of the 30 most abundant fungal genera based on their normalized relative abundances in each group. Color distribution of individual fungal genera is reported in color key based on normalized *Z* scores. The primate groups were arranged as per the hierarchical clustering shown in Fig. 1b. Cumulative relative abundance of individual genera across groups is shown in the separate bar plot. Genera highlighted in green and deepskyblue belong to the Ascomycota and Basidiomycota phyla, respectively. Tropic mode and guild of these most abundant genera were selected from the total assigned trophic mode and guild for each ASV. To make FUNGuild assignments more accurate, only ASVs ranked as “Probable” and “Highly Probable” hits were considered for final classification. ASVs ranked as “Possible” were not considered. These categories are shown at the bottom of the heatmap with different colors and numbers. The dotted boxes along with the asterisk are drawn to show statistical significance (based on species indicator analysis, indval > 0.3, and *p* < 0.05) of each fungal genera in the respective primate group. The identifiers used for primate groups are US-H: US-Human, CCO: Captive Chimpanzees Ostrava, CWLG: Captive Western Lowland Gorilla, MG: Mountain Gorilla, Ban-H: Bantu-Human, BaA-H: BaAka-Human, C**:** Chimpanzee, M**:** Agile Mangabey, and WLG: Western Lowland Gorilla.

The relative abundance of the 30 most abundant fungal genera is shown in a hierarchically clustered heatmap on Fig. 2. This analysis identified taxonomic signatures specific to individual primate populations and taxa that clustered different groups together based on ecological similarities. For example, *Trichosporon* and *Lactarius* distinguished the BaAka hunter gatherers, while *Pichia* characterized the Bantu agriculturalists; however, both human populations, who constituted a single cluster (Cluster4, Supplementary Fig. 2b, c), harbored high abundance of unidentified fungi of the Saccharomycetales order. US humans and all captive apes (chimpanzees and western lowland gorillas) (Cluster2) seemed to harbor the highest abundances of *Penicillium*; nonetheless, US humans were also uniquely distinguished by *Saccharomyces*, while *Sordaria* was more characteristic of captive western lowland gorillas. In contrast, the cluster distinguishing wild western lowland gorillas, chimpanzees, and agile mangabeys (Cluster3) showed the lowest levels of *Saccharomyces* across all primates analyzed, including humans. In addition, individual groups within the wild primates cluster showed high variability in the number of group-specific taxa. Remarkably, the cluster composed of only of mountain gorillas (Cluster1) was not characterized by any specific fungal indicator; however, Fig. 2 shows that there is high interindividual variability in the presence and abundance of specific fungal taxa detected in mountain gorillas, in concordance with the high variation in alpha diversity patterns observed in this group (Fig. 1a).

Ecological importance of these fungal genera was assessed by assigning them into predictive functional categories using FUNGuild, based on their probable guild source and trophic mode^17^. Guild source provides information on functional groups or categories of related or unrelated groups of species that likely exploit the same environmental resources. The assigned guilds (7 out of 12), show that *Cladosporium*, unassigned Didymosphaeriaceae, *Lasidodiplodia*, *Lactarius*, Unassigned Sordariomycetes, *Ganoderma* and Unassigned Capnodiales, all distinguishing traditional human populations and wild apes, are associated with plant material (Fig. 2). Indeed, the proportion of plant associated fungi was always greater in all wild apes compared with captive apes, and in Bantu traditional agriculturalists and BaAka hunter gatherers compared with US humans (Supplementary Fig. 4). Trophic mode information of these taxa, as predicted by FUNGuild, revealed potential interactions between each host and its mycobiome, showing variable relationships including saprotrophic, phototrophic and symbiotrophic (Fig. 2).

### Mycobiome and bacteriome interacted differently across primate populations, and were affected by ecological factors unique to each population and individuals within populations

**Fig. 3:**
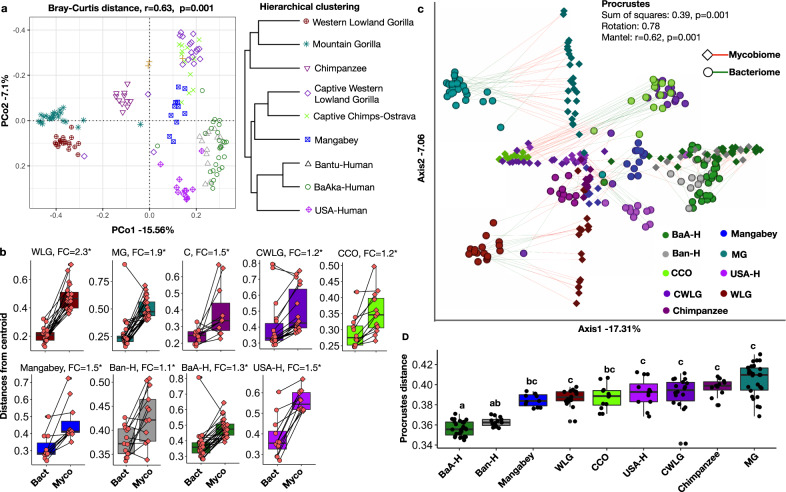
Combined gut bacteriome and mycobiome community composition differ across different primate species. **a** Principal coordinate analysis based on Bray-Curtis distances showing significant differences in combined bacteriome and mycobiome community composition across different primate groups. Each symbol represents the combined bacteriome and mycobiome composition at the ASV level in fecal samples of an individual primate species. A Bray-Curtis distance dendrogram (hierarchical clustering, method = average) based on mean abundances**. b** Distances to centroid show higher inter-individual variations in mycobiome community composition as compared to bacteriome across different primate groups. Fold changes along with the statistical significance based on wilcoxon rank-sum tests (*p* < 0.05, highlighted by *) are provided at the top of each boxplot (**c**) Correspondence between bacteriome and mycobiome composition as shown by Procrustes analysis on Bray-Curtis distances (Protest corr = 0.78, *p* = 0.001 and mantel test, *r* = 0.63, *p* = 0.001). **D** Procrustes distances between bacteriome and mycobiome composition in each primate group show greater correspondence in traditional human populations. The identifiers used for primate groups are US-H: US-Human, CCO: Captive Chimpanzee-Ostrava, CWLG: Captive Western Lowland Gorilla, MG: Mountain Gorilla, Ban-H: Bantu-Human, BaA-H: BaAka-Human, and WLG: Western Lowland Gorilla. In boxplots*,* center values indicate the median; bounds of box represents lower/upper quartiles; whiskers show inner fences.

Relative proportions of bacteriome and mycobiome in each primate group were accessed after merging the centered log-ratio (CLR) transformed compositional data separately (see “Methods”). Merged absolute CLR transformed counts indicated that abundance of bacteria was higher relative to that of gut fungi (bacteriome mean = 85.73%; mycobiome mean = 14.26%) (Supplementary Fig. 5). However, we noted that the mycobiome is structured differently across species compared with the bacteriome, and that interactions between mycobiome and bacteriome were specific to each primate population and individual without following a consistent pattern. Adding the mycobiome and bacteriome fractions together, resulted in ordination patterns that are slightly more concordant with a phylogenetic-driven dynamics^18^, except for captive apes and wild agile mangabeys, both of which harbored more similarities with all human groups (Fig. 3a).

Another important distinction is the significantly higher interindividual nature of the mycobiome relative to the bacteriome. For example, distance to centroid in ordination space, which indicates how dispersed individuals are relative to the average distance in their group, was on average 1.3 times greater for mycobiome compared with the bacteriome (Fig. 3b). This difference was the highest for western lowland gorillas and mountain gorillas (fold change for mycobiome:bacteriome disimilarity = 2.3 and 1.9, respectively, wilcoxon rank-sum tests, *p* < 0.05), who are the most folivorous primates in our dataset, and lowest for traditional BaAka hunter-gatherers and Bantu agriculturalists (1.1–1.3) (Fig. 3b). The US human group, remarkably, also showed high interindividual variation in mycobiome composition compared with the bacteriome.

Procrustes and mantel tests were applied to specifically test for correspondence between the bacteriome and mycobiome across all studied groups, indicating that the level of correspondence between these two microbial fractions across all populations studied is significant (Protest corr = 0.78, *p* = 0.001 and mantel test, *r* = 0.62, *p* = 0.001). However, correspondence patterns were unique to each species. For instance, the greatest correspondence between mycobiome and bacteriome (lowest distance) was observed in both the traditional human populations BaAka and Bantu, and the lowest correspondence (greater distance) in mountain gorillas (Fig. 3D). Greater overlap or correspondence between the mycobiome and bacteriome in the traditional human groups was evident when measuring the level of association between ordination scores obtained from mycobiome and bacteriome according to Bray-Curtis distances (plots in Fig. 1b and Supplementary Fig. 6b, respectively); this association analysis, seen in Supplementary Fig. 7a, shows how the samples that most overlapped in the combined ordination scores, reflected by their closeness to a regression line, are those of the BaAka hunter-gatherers and Bantu agriculturalists, followed by wild chimpanzees and agile mangabeys. In contrast, the samples that showed the greatest distance from the regression line between mycobiome and bacteriome were those of the two wild gorilla populations (Supplementary Fig. 7a).

Thus, similarities in mycobiome-bacteriome correspondence between groups that share some bacteriome traits (e.g., humans practicing non-industrialized subsistence strategies, wild chimpanzees and wild agile mangabeys) as reported before^19^ was not always observed; indeed, gorillas, all captive apes and US humans, all of which showed substantial ecological divergence showed a similar level of mycobiome-bacteriome dissimilarity (Fig. 3c and Supplementary Fig. 7a). Associations between fungal and bacterial richness (alpha diversity) when considering all groups were positive and significant, but weak (Spearman *r* = 0.36, *p* = 4.7e–06, Supplementary Fig. 7b). This observation indicates that greater bacterial richness did not necessarily correspond to higher fungal richness in the primate populations studied, which is perhaps more evident in the case of mountain gorillas (Supplementary Fig. 7b). However, low bacterial richness always corresponded with low fungal richness, specifically, in US humans. All analyses on bacterial diversity, including ordination, alpha diversity and taxonomic composition can be seen in Supplementary Figs. 6 and 8.

### Network dynamics between mycobiome and bacteriome also showed group-specific patterns

**Fig. 4:**
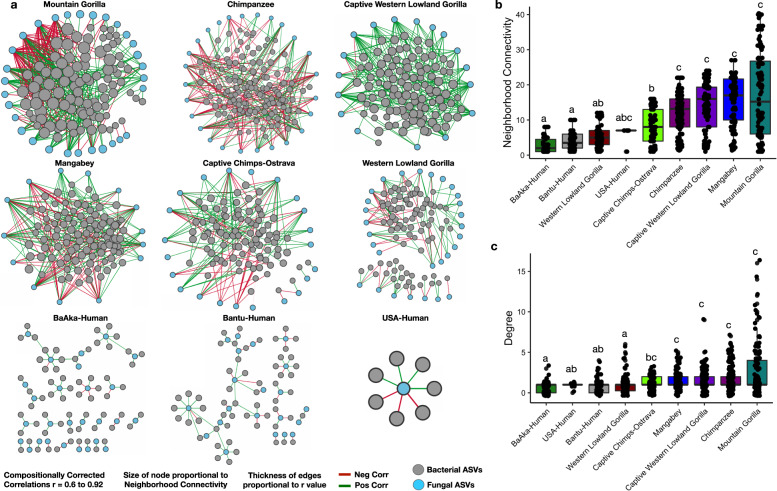
Complexity of bacteriome-mycobiome co-occurrence networks varies among different primate groups. **a** Co-occurrence networks constructed using all significant correlations (compositionally corrected corrs *r* > +/−0.6 and *p* < 0.01) showed clear distinctions in network complexity between human and nonhuman primates. Size of the node shows the number of connections of each fungal ASV. Color of nodes represents bacterial and fungal ASVs shown in Fig. 4a. Each symbol represents an individual primate species. Edge color represents negative (red lines) and positive (green lines) correlations respectively. Bacterial fungal co-occurrence network attributes (**b**) Neighborhood connectivity and (**c**) Degree shows a smaller number of bacterial fungal connections in all humans and in western lowland gorillas. Different letters denote significant differences according to Kruskal–Wallis tests. In boxplots, center values indicate the median; bounds of box represents lower/upper quartiles; whiskers show inner fences. The identifier used for primate groups are Mangabey: Agile Mangabeys and Chimps: Chimpanzee.

To understand the nature of specific associations between mycobiome and bacteriome, we performed a co-occurrence network analysis based on compositionally corrected correlations between bacterial and fungal ASVs in each primate group. We considered only significant positive and negative correlations (*r* > +/−0.6, *q* < 0.01), and calculated neighborhood connectivity, which denotes the number of local and wide (direct/indirect) interactions between fungal and bacterial ASVs^20^, as well as modularity, degree and hub scores. These primate-specific networks showed significant differences in the number of mycobiome-bacteriome interactions (Fig. 4a and Supplementary Fig. 9a), with all nonhuman primates showing denser networks (higher number of bacteria-fungi associations) but lower modularity (dense connections between nodes across different modules) relative to all humans. However, the gut microbiome of US humans always showed the fewest number of associations between bacteria and fungi; for example, only one fungal taxon was found associated with a few bacterial species *(r* > 0.6, *q* < 0.01), therefore, the US-humans network displayed zero modularity.

Denser networks in nonhuman primates were further supported by the higher number of observed hub nodes, which ranged from 7 to 31. This metric was based on a hub score >0.2, which measures the number of nodes significantly more connected within a network. This observation is in contrast with all humans, which showed hub nodes ranging from 1 to 5. (Supplementary Table 2, all hub nodes based on high hub score >0.2 are highlighted in green). Additionally, the degree of connectivity of microbial communities in each sample was assessed using a cohesion metric, for negative and positive correlations separately. These analyses showed similar patterns for positive and negative cohesion, demonstrating that high or low cohesion values do not correlate with ecological niche; for example, wild and captive chimpanzees and the wild mountain gorillas showed high positive and negative cohesion values. However, US humans always displayed the lowest cohesion values compared with any other group (Supplementary Fig. 10). We didn’t find an influence of number of samples per group on number of significant correlations detected in each primate group (Supplementary Fig. 9c).

Neighborhood connectivity, degree, modularity, and cohesion (Fig. 4b, c, Supplementary Figs. 9a, b, and 10) showed that each primate group displayed unique mycobiome-bacteriome interactions. For instance, mountain gorillas displayed the greatest number of interactions between fungi and bacteria. This is an interesting observation considering mountain gorillas showed the lowest correspondence between these two microbial fractions (Fig. 3c, d) and poor associations between fungal and bacterial diversity (Supplementary Fig. 7b). Likewise, despite the fact that the agricultural and foraging human populations showed the greatest degree of correspondence between bacteriome and mycobiome composition, they still showed less dense networks and network connectivity compared with all nonhuman primates in this study (Figs. 4a, b, c). However, US humans were the only group that consistently showed a low degree of association between mycobiome and bacteriome, low mycobiome and bacteriome diversity and low number of interactions between these two microbial fractions. Thus, in most cases, alpha diversity of bacteria and fungi, and similarities between mycobiome and bacteriome composition do not explain co-occurrence or interaction patterns between specific bacteria and fungi in the primate gut.

**Fig. 5:**
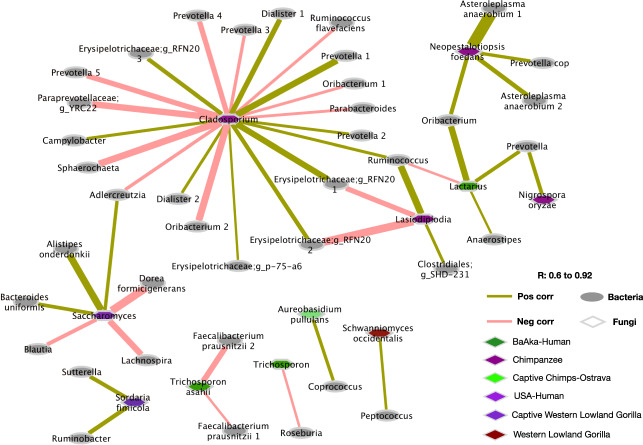
Associations between bacterial taxa and representative fungal genus of individual primate groups. Compositional based correlations between all ASVs of significantly discriminating (species indicator analysis, *p* < 0.01) fungal genus and bacterial ASVs to show co-occurring (pairs connected using citrus color) and co-exclusive (pairs connected using light salmon pink) fungal bacterial pairs. Color code in the bottom of the plot shows the enrichment of fungal genus in respective primate groups. Shape of the node represents bacterial and fungal ASVs. Edge thickness represents correlation strength.

Identification of specific interactions between fungal taxa distinguishing a given primate group (e.g., indicator taxa as shown in Fig. 2) and any bacterial taxa, also revealed unique patterns (Fig. 5 and Supplementary Fig. 11). For example, wild chimpanzees showed multiple associations between *Cladosporium* and various gut bacterial commensals (e.g., positive with *Prevotella*, *g__RFN20*, *Dialister*, *Campylobacter*, *Ruminococcus*, and *g__p-75-a6;* negative with *Parabacteroides, Oribacterium, Ruminococcus flavefaciens, Prevotella, Adlercreutzia, g__YRC22*, and *Sphaerochaeta). Cladosporium* was identified as one of the hub taxa in wild chimpanzees, based on hub score of 0.23 (Supplementary Table 2) and was classified as plant-associated pathotroph/symbiotroph according to FUNGuild. This observation contrasts with the only gut fungal marker of western lowland gorillas, *Schwanniomyces*, which only showed one positive association with *Peptococcus*. However, none of the unique fungal markers of the remaining two wild primate populations, agile mangabeys and mountain gorillas, showed any significant associations with bacterial taxa (*r* > 0.6, *q* < 0.01).

Captive groups also showed a few associations between their indicator fungi and bacterial taxa. For example, *Aureobasidium pullulans*, the gut fungal marker of captive chimpanzees, showed a positive association with *Coprococcus;* whereas *Sordaria*, found in captive western lowland gorilla and classified as a saprotroph that feeds on decaying wood matter according to FUNGild, showed positive associations with *Ruminobacter* and *Sutterella*. A few group-specific association patterns also characterized the three human populations; *Lactarius*, key taxa in BaAka hunter gatherers based on the network hub score 1; (Supplementary Table 2) and classified as a plant symbiotroph, showed positive associations with *Oribacterium, Prevotella and Anaerostipes*, and a negative association with *Ruminococcus. Trichosporon,* a pathotroph also distinguishing the BaAka, showed negative associations with *Roseburia* and *Faecalibacterium prausnitzii*. The only gut fungal marker of the US human population, the pathotroph *Saccharomyces*, key taxa based on a network hub score =1 (Supplementary Table 2), showed positive associations with *Alistipes onderdonkii*, *Bacteroides uniformis*, *Adlercreutzia* and negative associations with *Blautia*, *Lachnospira* and *Dorea formicigenerans*. However, the fungal makers of Bantu agriculturalists, *Pichia* and unclassified Saccharomycetales, did not show association with any bacterial taxa (Fig. 5, Supplementary Fig. 11).

## Discussion

This report presents an overview of the gut mycobiome and its interactions with bacteria in different wild and captive nonhuman primates, and human populations with diverse subsistence strategies. The results reinforce the important role of diet and subsistence strategy over phylogeny in shaping the primate gut mycobiome and bacteriome. However, the data indicate that, compared with the gut bacteriome, gut fungal communities in primates are more variable, and perhaps more driven by environmental, individual and/or intrinsic factors characterizing populations and specific individuals within each population.

### Dietary, environmental and individual factors have a greater influence on shaping the primate gut mycobiome than the bacteriome

Just as reported with the bacteriome^19,21–23^, these results indicate that the primate gut mycobiome is significantly driven by ecological and subsistence factors over the phylogenetic background of the host. However, fungal fingerprints in the groups studied were significantly more variable compared with the bacteriome, indicating that gut fungi may be even more influenced by environmental, individual or behavioral factors, concordant with previous data reported in healthy humans^24^. This contention is further supported by our findings: weaker influence of phylogenetic constraints and higher inter-individual variability within groups in mycobiome composition, weak correspondence between fungal and bacterial community assemblies, and inconsistent nature of interactions observed between fungi and bacteria in the primate gut. In line with these data, the strong influence of dietary and environmental sources in seeding the gut mycobiome has been reported recently in mice^10^ and nonhuman primates^16,25,26^. Compared with the bacteriome, the gut environment may be particularly susceptible to food-derived and environmental fungi, albeit transiently^27^, possibly triggering the heterogeneous compositional patterns we observed between individuals within the same primate population.

It is unclear why the BaAka hunter gathers showed the greatest correspondence (and less inter-individual variation) between bacterial and fungal composition compared to any other group studied, including US humans and the folivorous mountain gorillas. This observation warrants further investigation, especially in regards to how interactions between diet, fungi and bacteria contribute to ecological assembly and structural resilience in the human gut, and how these interactions define patterns of health and disease in the host^2^. It was clear that US humans exhibited poor correspondence and the lowest degree of connectivity between bacterial and fungal fractions, and low fungal alpha diversity compared to the traditional human groups and any other nonhuman primate group analyzed. This observation is remarkable in light of the previously hypothesized connections between western diets, extinction of the gut microbiome, and diseases of civilization^28,29^. It is possible that high sanitation of western food systems impedes seeding of food-derived fungi in western subjects^30^, which is in contrast with the higher number of food or environmental derived fungi we observed in the nonhuman primates and traditional human populations. Thus, our data generate questions on the role of food-associated microorganisms and their impact on human health and the gut mycobiome in particular, as a symbiotic population affected by food sanitation and industrialized subsistence patterns; these potential associations had previously focused on the bacteriome only.

The observation that merging the bacteriome and mycobiome in ordination space results in patterns more consistent with a phylogenetic-based arrangement of each primate group, indicates that, in primates, the host genetic background may have more influence over the gut bacteriome than the mycobiome. Weak phylosymbiosis between fungal commensals and hosts has been shown previously, including other microbial populations in the gut of various wild primates (e.g., with nematodes)^11^. For instance, we did not observe a mycobiome overlap between the two closely related lowland and mountain gorillas, as reported before with bacterial communities^19^. Instead, wild lowland gorillas shared mycobiome similarity with wild chimpanzees and wild agile mangabeys, which are more phylogenetically divergent. Mycobiome convergence between these wild primate populations could be explained by similar feeding behaviors, specifically by a similar degree of reliance on fruit^31–33^, but also by environmental seeding sources from plants and soil shared in a common niche^16,25^. However, selectivity and preference of specific fruit substrates vary greatly among wild primates, even when sharing the same location and resources, which may explain why - although a mycobiome overlap was observed between these three species - each shows a set of unique indicator fungal taxa. These indicator taxa, including Schwanniomyces^34^, *Cladosporium*^35^, *Lasiodiplodia*^36^, *Nigrospora*^37^ and *Hanseniaspora*^38^ are all yeast commonly associated with specific plant foods, including leaves and fruits, concordant with their classification as plant-associated fungi according to FUNGuild. However, it is also likely that these wild primates purposely forage on fungi (mycophagy), as it has been reported for wild bonobos, chimpanzees and even mountain gorillas, the later of which are reported to specifically forage on *Ganoderma*, consistent with the compositional mycobiome patterns observed in this population (Fig. 2)^39–41^. *Penicillium*, a taxon common to captive apes and US humans, colonizes a wide variety of food items, which indicates that their presence in these groups may also be explained by dietary or environmental sources and not due to permanent colonization in the gut^27,42,43^. These data indicate that collective and individual feeding behaviors have a strong influence in shaping the seeding of fungal communities in the primate gut. However, the specific dietary and/or environmental determinants of these fungal patterns in every group of wild and captive primates and US humans remains unknown.

The enrichment of *Lactarius* and *Pichia* in the BaAka hunter-gatherers and Bantu agriculturists, respectively, may be better explained by specific dietary behaviors. For example, *Lactarius* is a mushroom genus that includes several edible species^44^, concordant with the observation that African foragers pick and consume staple mushrooms frequently^45–47^. Edible *Lactarius* is reported to have antitumor, antioxidant, and immunostimulant functions^48,49^. *Pichia*, a fungal taxon characterizing the Bantu agriculturalists, is common in many traditional, cereal-based fermented African foods^50,51^, and have been deemed to have probiotic properties including antioxidant and cholesterol-lowering effects^52^. *Saccharomyces*, common in all human populations studied, are found in several foods and beverages^53^, and are also reported to have probiotic properties^54^. These observations provide additional evidence on the potential role that diet-derived microorganisms, including fungi, play when reaching the human gut and influencing health^55^. Thus, an important question centers on elucidating the extent to which the presence of these fungi in the primate gut, many of them likely associated with external, dietary sources and hence largely transient in the gastrointestinal tract, represents any digestive, nutritional or health consequences for the host. Based on this dataset, which is compositional in nature, it is not possible to make such functional inferences. However, we sought to explore if mapping associations between fungal communities and the bacteriome could help in elucidating the ecological relevance, in the gut environment, of the primate mycobiome.

### Mycobiome-bacteriome interactions shed light on the ecological and physiological contributions of the gut mycobiome in primates

Patterns of association between fungi and bacteria may offer clues to understand the ecological and physiological relevance of fungi in different primate hosts. For instance, it has been shown that diet alterations impact interkingdom metabolic networks, with potential physiological consequences for the host^10^. Indeed, co-abundance patterns between bacteria and fungi have been shown to be significantly affected by western diets^56^. These observations may help explain the lower number of interactions observed between gut bacteria and fungi in humans compared with nonhuman primates. Particularly, US humans showed the least dense networks, the lower neighborhood connectivity, degree, low number of hub taxa, and lower modularity. It is likely that less reliance on dietary fiber, specifically depleted in western diets, has affected potential metabolic interactions between the mycobiome and the bacteriome in humans along evolutionary timescales. The bacteria-fungi co-abundance network analyses could also indicate that high fiber diets, such as those characterizing the most folivorous primates (e.g., gorillas), require increased synergistic interactions between bacteria and fungi in the gut. However, since captive gorillas and chimpanzees, which likely subsist on less dietary fiber, showed a similar degree of connectivity, dietary fiber may not always predict interactions or metabolic synergism between bacteria and fungi in the primate gut. An additional, possible explanation is that the foods consumed by nonhuman primates, regardless of specific diet, exhibit a higher content of food-associated fungi that can reach the gut environment. In this regard, it has been shown that more diverse diets (fruits, vegetables, lean meat and whole grains) exhibit a higher microbial load (including yeast and bacteria) compared with typical American diets^57^, which could affect the rate at which fungi and bacteria interact in the gut.

Also, these data show that fungal or bacterial diversity in the primate gut do not necessarily correspond with the number of interactions between bacteria and fungi. For instance, mountain gorillas exhibit low, and highly variable fungal diversity, and bacterial diversity that is comparable to that seen in all other nonhuman primates and traditional human populations. Yet, mountain gorillas show the densest, most connected bacterial-fungi co-occurrence networks. In contrast, the traditional human populations showed higher fungal and similar bacterial diversity as compared to mountain gorillas; nonetheless, they showed the least number of interactions. An interesting question to pursue, in light of these findings, is to determine the extent to which degree of external microbial seeding is a more relevant factor in predicting associations between fungi and bacteria in the primate gut. Based on a greater influence of environment in shaping gut fungi, in contrast to bacteria, as proposed here, one could speculate that the degree of fungal seeding from foods and the environment may be an important determinant of the number of bacteria-fungi interactions. In that case, non human primates would be more likely to be seeded from external sources, compared to any human, but particularly, compared to US humans. Moreover, if most fungi are mainly present partly due to infections from external sources, one would expect that the number of fungi-bacteria interactions would vary significantly across spatiotemporal scales (e.g., season)^58^. These observations highlight the importance of environmental cues in shaping cooperation and/or competition among diverse host-associated microbial kingdoms^59^.

Fungal and bacterial taxa with similar functional potential may interact to accomplish common metabolic roles; such may be the case of *Lasiodiplodia* in wild chimpanzees, which is known to produce various enzymes involved in plant cell wall degradation^60,61^, and *Ruminococcus* and Clostridiales, typical symbionts associated with fiber-degradation roles^62,63^. Antagonistic interactions may show competition for common ecological niches but can also represent a physiological advantage for the host. For example, *Faecalibacterium* and *Roseburia*, which are butyrate producers and are associated with optimal intestinal integrity and immune modulation^64^, were seen to antagonize with *Trichosporon* in the BaAka hunter-gatherers; this fungus is a classical opportunistic pathogen whose infection causes trichosporonosis^65^. This observation adds to the growing body of evidence pointing to the mycobiome, and their interactions with the bacteriome, as key factors shaping health and disease phenotypes in the host through pathogen or inflammation control^2,66,67^. Bacteria may also directly degrade or metabolize fungal cell walls, which are rich in α-mannans^68^, potentially supporting the multiple associations observed between different fungi, and bacterial taxa typically regarded as fibrolytic, saccharolytic, or fermentative^64^. However, the functional relevance of these bacteria-fungi interactions in the primate gut, in terms of their physiological consequences for the host, remains unknown.

In summary, these data highlight the role of environment and subsistence strategy in shaping gut mycobiome structure and mycobiome-bacteriome interactions in different primate populations. The weak phylogenetic-based assortment and high inter-individual variation of fungal communities, relative to the bacteriome, indicates that ecological, behavioral and individual factors define the assembly and persistence of fungal communities and the degree to which fungi interact with bacteria in the primate gut. However, these data cannot assess the mechanisms that dictate gut fungal community assembly and environmental seeding, as it pertains to each primate species or their specific ecological niche. Nor can we assess the metabolic or phenotypic impact of these interactions (or lack of thereof). Moving forward it would be key to measure the extent to which soil and specific food sources in the ecological niche of each primate host determine mechanisms of fungal assembly in their gastrointestinal tract. Likewise, the relevance of transient fungi in the primate gut needs to be elucidated beyond in silico analyses, focusing on interactions between environment, diet, bacteria, and fungi and the consequences of these interactions for the host physiological landscape.

## Methods

### Subjects and samples

Samples from four social groups of western lowland Gorillas (*Gorilla gorilla gorilla*, *n* = 19), one group of agile Mangabeys (*Cercocebus agilis*, *n* = 11), BaAka hunter-gatherers (*n* = 27), and Bantu agriculturalists (*n* = 13) were collected at the Dzanga Sangha Protected Areas, Central African Republic. Samples from one group of eastern chimpanzees (*Pan troglodytes schweinfurthii, n* = 11) were collected in Bulindi, Uganda. Samples from captive western lowland gorillas (*n* = 18) were collected at Como zoo in St Paul Minnesota, USA (with some individuals sampled up to three times), while samples from captive chimpanzees were collected at Ostrava (*n* = 12) zoos in the Czech Republic. Samples from mountain gorillas (*Gorilla beringei beringei*, *n* = 26) from four social groups were collected at Bwindi Impenetrable National Park, Uganda. Samples from the US human population (*n* = 12) were collected from healthy subjects in St. Paul MN. All samples were collected between 2012 and 2016. About 1 gr of fecal sample, taken from the inner core of feces, avoiding the exterior and within 1–2 h after voiding, was collected and then stored in 5 ml tubes containing RNAlater (Qiagen, Germany, 1 g of fecal sample in 2 volumes of solution). Then, the solution was mixed thoroughly to homogenize the sample. Depending on field/site infrastructure, samples remained at room temperature from 24 h for a maximum of three weeks before storage at −20 ^o^C until DNA extraction^22,69–71^.

#### Ethics

##### Human samples

Approval to collect samples from US humans was granted by the University of Minnesota, Twin Cities, and its Institutional review board (IRB) for the protection of human subjects, protocol number STUDY00004208. All work carried out with hunter-gatherers and agriculturalists from the Central African Republic, including sample collection, was approved according to the rules and regulations from the Ministre de l’Education Nationale, de l’Alphabetisation, de l’Enseignement Superieur, and de la Recherche (Central African Republic), as well as the IRB for the protection of human subjects from the University of Illinois at Urbana-Champaign (permit number 13045). All participants provided written informed consent to take part in the study.

##### Nonhuman primate samples

Samples from wild western lowland Gorillas and agile mangabeys from the Central African Republic were collected with approval by the Ministre de l’Education Nationale, de l’Alphabetisation, de l’Enseignement Superieur, and de la Recherche (Central African Republic) while samples from wild mountain gorillas were collected with approval from the Uganda Wildlife Authority and the Uganda National Council for Science and Technology. Samples from wild chimpanzees were collected with approval from Makerere University in Uganda, protocol number HDREC421 and from Oxford Brooks University in the UK, protocol number UREC-160989. Samples from captive apes from St. Paul, Minneapolis, USA and Europe were collected under protocol ID 2003-37934A granted by the University of Minnesota, Twin Cities.

### DNA extraction, amplicon library preparation, sequencing and data processing

Genomic DNA was extracted using the Power Soil DNA extraction kit of MoBio (Carlsbad, CA). To determine bacterial composition, the V4 variable region of the 16 S rRNA gene was amplified using 16S-515F (GTGCCAGCMGCCGCGGTAA) and 16S-806R (GGACTACHVGGGTWTCTAAT) primers. To determine fungal composition, the internal transcribed spacer 2 (ITS2) was amplified using ITS3 (GCATCGATGAAGAACGCAGC) and ITS4 (TCCTCCGCTTATTGATATGC) primers. Sequencing of pooled libraries was carried out using Illumina MiSeq platform at the University of Minnesota to generare 2*300 bp of sequences, including negative controls (libraries constructed on a water solution). 16 S rRNA and ITS2 sequences were processed using custom-made Perl scripts and the Qiime2 pipeline^72^. Raw sequencing data were processed to remove primers and low-quality reads (phred quality score < 30) using cutadapt and fastx_toolkit, respectively. These high-quality reads were considered for denoising, merging, chimera removal and finally to generate unique amplicon sequence variants (ASV) using the Dada2 plugin of Qiime2. Representative sequences of each ASV were aligned using MAFFT and phylogenetic trees both rooted and unrooted were constructed using FasTree. Taxonomic assignments of bacterial ASVs were carried out by trained naive Bayes classifiers on reference sequences (clustered at 99% sequence identity) from Greengenes 13_8, and fungal ASVs were carried out using the UNITE database. For both taxonomic assignments, Qiime2 plugins feature-classifier fit-classifiernaive-bayes and feature-classifier classifier-sklearn were used. Generated bacterial and fungal ASV tables were converted to relative proportions using total reads per sample and the ASVs which were not present in at least 5 samples (~3% of total samples) were omitted from the data set. Furthermore, bacterial, and fungal taxa present in both negative control samples were identified and removed before using bacterial and fungal relative abundances at different taxonomic levels for downstream analysis.

### Guild analysis

For assigning ecological functions to each fungal ASV, we have used the FUNGuild program. ASVs for which tropic modes and guilds were identified as “probable” and “highly probable” were considered for further downstream analysis.

### Microbial networks

Bacterial fungal association networks for each microcosm were created by first creating a compositionally corrected correlation matrix using CCREPE function in R^73^. All strong significant correlations (*r* > +/−0.6 and *q* < 0.01) were then used in the Cytoscape to generate undirected networks^74^. These networks were used to calculate the various network parameters such as neighborhood connectivity, degree, and cohesion along with the keynote taxa based on hubScore and modularity of network using the Igraph package in R^75^. From these networks, the number of interactions was counted, and network complexity specifically to understand overall connectivity of microbial communities was reported using degree, neighborhood connectivity, modularity and positive/negative cohesion.

### Statistical analyses

All microbial community analyses were performed within the R statistical interface. Briefly, for alpha diversity, beta diversity, permutational multivariate analyses of variance (PERMANOVA), multiple R packages such as vegan, ape, phyloseq were used^76–78^. CLR transformation was done on each table separately to estimate the relative proportions of mycobiome and bacteriome using. Significantly discriminating bacterial and fungal taxa were identified using species indicator analysis using labdsv package in R. Average genus abundances of each primate group were used to generate phylogenetic trees based on Bray-Curtis distances and hclust function within the R ape package. Bacterial and fungal summary analyses were performed on the phyloseq object using plot_bar function in R. Kruskal–Wallis tests were used to check the statistical significance among multiple groups using the kruskalmc function of pgirmess package in R^79^, whereas, Wilcoxon rank-sum tests were used to check the statistical significance among sbacterial and fungal interindividual variation within each primate group using the wilcox.test function in R. Heatmap was generated using aheatmap function in R NMF package^80^. All graphs were plotted using ggplot function in R^81^.

### Reporting summary

Further information on research design is available in the Nature Research Reporting Summary linked to this article.

## Supplementary information

Supplementary material

Reporting Summary

## Data Availability

16 S rRNA and ITS2 sequences generated in this study have been deposited in the NCBI SRA under the BioProject identifier (ID) code: PRJNA686661.

## Code availability

All relevant codes are available on https://github.com/ashoks773/mycobiome_bacteriome. Other supporting data/information will be available from the corresponding author upon request.
