# Supplementary material for: The primate gut mycobiome-bacteriome interface is impacted by environmental and subsistence factors
Source: NPJ Biofilms Microbiomes. 2022 Mar 17;8:12. doi: 10.1038/s41522-022-00274-3 (PMC8930997; doi:10.1038/s41522-022-00274-3)
Supplement: Supplementary file 1 — Supplementary material [file 41522_2022_274_MOESM1_ESM.pdf]

Supplementary Table 1. Taxonomic signatures in each primate group were identified using indicator species analysis.

Family	group	indval	pvalue
Basidiomycota.Russulaceae	BaAka-human	0.48	0.001
Basidiomycota.Trichosporonaceae	BaAka-human	0.32	0.017
Basidiomycota.Polyporaceae	BaAka-human	0.31	0.004
Ascomycota.Pichiaceae	Bantu-Human	0.59	0.001
Basidiomycota.Agaricostilbaceae	Bantu-Human	0.56	0.001
Ascomycota.unassigned.Saccharomycetales	Bantu-Human	0.51	0.001
Basidiomycota.Wallemiaceae	Bantu-Human	0.45	0.002
Ascomycota.Ascodesmidaceae	Bantu-Human	0.38	0.003
Ascomycota.unassigned.Capnodiales	Captive chimps-Hodonin	0.49	0.007
Ascomycota.Niessliaceae	Captive chimps-Hodonin	0.33	0.007
Ascomycota.Leptosphaeriaceae	Captive chimps-Hodonin	0.33	0.019
Ascomycota.Arachnomycetaceae	Captive chimps-Hodonin	0.32	0.013
Basidiomycota.Fomitopsidaceae	Captive chimps-Hodonin	0.31	0.004
Basidiomycota.Holtermanniales_fam_Incertae_sedis	Captive chimps-Hodonin	0.31	0.022
Ascomycota.Magnaporthaceae	Captive chimps-Hodonin	0.31	0.013
Basidiomycota.Erythrobasidiaceae	Captive chimps-Hodonin	0.31	0.017
Basidiomycota.Sporidiobolaceae	Captive chimps-Ostrava	0.46	0.004
Ascomycota.Aureobasidiaceae	Captive chimps-Ostrava	0.41	0.007
Ascomycota.Pleosporaceae	Captive chimps-Ostrava	0.33	0.018
Basidiomycota.Filobasidiaceae	Captive chimps-Ostrava	0.30	0.017
Ascomycota.Lasiosphaeriaceae	Captive western lowland gorilla	0.47	0.003
Ascomycota.Sordariaceae	Captive western lowland gorilla	0.42	0.007
Mucoromycota.Pilobolaceae	Captive western lowland gorilla	0.33	0.014
Ascomycota.Aplosporellaceae	Chimpanzee	0.63	0.001
Ascomycota.unassigned.Xylariales	Chimpanzee	0.59	0.003
Ascomycota.Chaetothyriales_fam_Incertae_sedis	Chimpanzee	0.56	0.002
Ascomycota.Trichosphaeriaceae	Chimpanzee	0.52	0.002
Ascomycota.Botryosphaeriaceae	Chimpanzee	0.51	0.006
Ascomycota.Periconiaceae	Chimpanzee	0.47	0.007
Ascomycota.Sporocadaceae	Chimpanzee	0.46	0.003
Ascomycota.Coniothyriaceae	Chimpanzee	0.45	0.003
Basidiomycota.Rhynchogastremataceae	Chimpanzee	0.45	0.001
Ascomycota.unassigned.Dothideomycetes	Chimpanzee	0.44	0.007
Ascomycota.Corynesporascaceae	Chimpanzee	0.43	0.001
Basidiomycota.Tremellaceae	Chimpanzee	0.42	0.002
Ascomycota.Cladosporiaceae	Chimpanzee	0.38	0.012
Ascomycota.Mycosphaerellaceae	Chimpanzee	0.38	0.01
Ascomycota.unassigned.Sordariomycetes	Chimpanzee	0.36	0.05

Ascomycota.unassigned.Lecanorales	Chimpanzee	0.34	0.002
Ascomycota.unassigned.Pleosporales	Chimpanzee	0.32	0.033
Basidiomycota.Bulleribasidiaceae	Chimpanzee	0.30	0.01
Basidiomycota.Psathyrellaceae	Mangabey	0.55	0.002
Ascomycota.Lophiostomataceae	Mangabey	0.51	0.003
Ascomycota.Capnodiaceae	Mangabey	0.47	0.001
Ascomycota.Hypocreales_fam_Incertae_sedis	Mangabey	0.45	0.003
Ascomycota.unassigned.Hypocreales	Mangabey	0.42	0.006
Basidiomycota.Agaricaceae	Mangabey	0.40	0.016
Ascomycota.Saccharomycetales_fam_Incertae_sedis	Mangabey	0.40	0.007
Basidiomycota.Hydnodontaceae	Mangabey	0.40	0.004
Ascomycota.Cordycipitaceae	Mangabey	0.40	0.018
Ascomycota.Sympoventuriaceae	Mangabey	0.38	0.011
Ascomycota.Elaphomycetaceae	Mangabey	0.35	0.002
Ascomycota.Saccharomycetaceae	USA-Human	0.30	0.044
Ascomycota.Saccharomycodaceae	Western lowland gorilla	0.49	0.004
Ascomycota.Didymosphaeriaceae	Western lowland gorilla	0.43	0.036
Ascomycota.Debaryomycetaceae	Western lowland gorilla	0.43	0.009
Basidiomycota.unassigned.Tremellales	Western lowland gorilla	0.36	0.015
Ascomycota.Archaeorhizomycetaceae	Western lowland gorilla	0.35	0.001
Mucoromycota.unidentified	Western lowland gorilla	0.32	0.01

Supplementary Table2. Sheet1. Key taxa in BaAka-Human identified based on the network hub score.

ASV	Hub Score	Taxa
a5df85791aea1c3c93996213e511e798	1	k__Fungi;p__Basidiomycota;c__Agaricomycetes;o__Russulales;f__Russulaceae;g__Lactarius
f86825cfba011a54bc8799a74662a8a8	0.225544475	k__Bacteria; p__Firmicutes; c__Clostridia; o__Clostridiales; f__Lachnospiraceae
f70335502c78e0a62f836a8ff964ff66	0.194884143	k__Bacteria; p__Bacteroidetes; c__Bacteroidia; o__Bacteroidales; f__[Paraprevotellaceae]; g__; s__
X1c8b530be4fb53dd9556a1cbf028f3f2	0.178331453	k__Fungi;p__Ascomycota;c__Dothideomycetes;o__Capnodiales;f__Cladosporiaceae;g__Cladosporium;s__Cladosporium_sphaerospermum
b2c9343b51ebe2282da3eda2fafb8693	0.168864018	k__Bacteria; p__Firmicutes; c__Clostridia; o__Clostridiales; f__Lachnospiraceae; g__Oribacterium; s__
X2b67477b5db889cc9274e8eed8e81bd0	0.168864018	k__Bacteria; p__Bacteroidetes; c__Bacteroidia; o__Bacteroidales; f__Prevotellaceae; g__Prevotella; s__
e2f5328726e4b0bb7e365a0c1d9e85b7	0.168864018	k__Bacteria; p__Firmicutes; c__Clostridia; o__Clostridiales; f__Ruminococcaceae; g__; s__
X24284cec3ba6a25cbdeb7322cc8450f8	0.168864018	k__Bacteria; p__Verrucomicrobia; c__Verruco-5; o__WCHB1-41; f__RFP12; g__; s__
a55a010c9525ce2943a553dca1421b1c	0.168864018	k__Bacteria; p__Firmicutes; c__Clostridia; o__Clostridiales; f__Lachnospiraceae; g__Anaerostipes; s__
df14354ec277348c54d0c9dd49eb4da0	0.168864018	k__Bacteria; p__Firmicutes; c__Clostridia; o__Clostridiales; f__Ruminococcaceae; g__Ruminococcus; s__
X79a2af8f3676378c234767c28b52a70f	0.157325943	k__Fungi;p__Basidiomycota;c__Agaricomycetes;o__Russulales;f__Russulaceae;g__Lactarius;s__unidentified
X406879750b15643051eadb1d747dafd5	0.154089221	k__Fungi;p__Ascomycota;c__Sordariomycetes;o__Microascales;f__Microascaceae;g__Scedosporium;s__Scedosporium_prolificans
X49153948ebd600987669e92e9934960a	0.030113766	k__Bacteria; p__Firmicutes; c__Clostridia; o__Clostridiales; f__Lachnospiraceae; g__; s__
c139024f4ea8e9e81fa0e7a36a10a36b	0.026020125	k__Bacteria; p__Bacteroidetes; c__Bacteroidia; o__Bacteroidales; f__; g__; s__
X6916d386b67ae389d75219a14bf80740	1.07E-15	k__Fungi;p__Ascomycota;c__Dothideomycetes;o__Capnodiales;f__Cladosporiaceae;g__Cladosporium
X0a7fe3595e40caeafecb26f55ecf526d	2.31E-16	k__Fungi;p__Ascomycota;c__Dothideomycetes;o__Capnodiales;f__Cladosporiaceae;g__Cladosporium
X274d44a105684512d6df0a983a5b6663	1.64E-16	k__Bacteria; p__Firmicutes; c__Clostridia; o__Clostridiales; f__Clostridiaceae; g__02d06; s__

X73cd65bf815e718c410a590cf1e58392	1.31E-16	k__Bacteria; p__Firmicutes; c__Clostridia; o__Clostridiales; f__; g__; s__
d54f71f28a2092111fd16b901d39254c	1.31E-16	k__Bacteria; p__Firmicutes; c__Clostridia; o__Clostridiales; f__[Mogibacteriaceae]; g__; s__
b66a7645cdaca2472b4a6371220803b1	1.31E-16	k__Bacteria; p__Tenericutes; c__Mollicutes; o__RF39; f__; g__; s__
cd86e3c16167d3db6cc1d8ae8b8b8348	1.31E-16	k__Bacteria; p__Firmicutes; c__Clostridia; o__Clostridiales
X0b4aeb45916a24fd10e3655fe6481966	1.31E-16	k__Bacteria; p__Firmicutes; c__Clostridia; o__Clostridiales; f__Christensenellaceae; g__; s__
cd7b0604bdc8b4cf6c69e0a150266791	9.63E-17	k__Fungi;p__Basidiomycota;c__Agaricomycetes;o__Agaricales;f__Lyophyllaceae;g__Termitomyces
X5ef58296652d5359831628286945e59f	9.63E-17	k__Fungi;p__Ascomycota;c__Saccharomycetes;o__Saccharomycetales
a9e2afd4297fd011649a216315249232	3.01E-17	k__Bacteria; p__Bacteroidetes; c__Bacteroidia; o__Bacteroidales; f__Prevotellaceae; g__; s__
X69f869ff6af6e63d8debb202e7eb73e7	3.01E-17	k__Bacteria; p__Firmicutes; c__Clostridia; o__Clostridiales; f__Ruminococcaceae; g__; s__
X421cbd1d71d34704c6d53377261e213c	1.11E-17	k__Bacteria; p__Firmicutes; c__Clostridia; o__Clostridiales; f__Lachnospiraceae; g__Anaerostipes; s__
X05760923db45c20388aeb2ff51ac3f2e	1.11E-17	k__Bacteria; p__Tenericutes; c__Mollicutes; o__RF39; f__; g__; s__
ab149525cad479f6599c7923acc49691	1.11E-17	k__Bacteria; p__Firmicutes; c__Clostridia; o__Clostridiales; f__; g__; s__
e671233da36fe99a7ce1b39ccbddd2c0	1.11E-17	k__Bacteria; p__Firmicutes; c__Clostridia; o__Clostridiales; f__Ruminococcaceae; g__; s__
X7c104699e795bbf45f90fceac906e6b4	1.11E-17	k__Bacteria; p__Firmicutes; c__Clostridia; o__Clostridiales; f__; g__; s__
X7717c98118439762317647199c6005f7	1.11E-17	k__Bacteria; p__Firmicutes; c__Clostridia; o__Clostridiales; f__Ruminococcaceae; g__; s__
d76d59ec71de0e3b22da0c9cd564d41a	1.11E-17	k__Bacteria; p__Firmicutes; c__Clostridia; o__Clostridiales; f__Lachnospiraceae
X45c80843dac191f771b11018ff90d94c	1.11E-17	k__Bacteria; p__Firmicutes; c__Clostridia; o__Clostridiales; f__Ruminococcaceae; g__Ruminococcus; s__callidus
d71feb1923fa288790187009e8efa6d3	1.11E-17	k__Bacteria; p__Firmicutes; c__Clostridia; o__Clostridiales; f__Lachnospiraceae; g__Blautia; s__

c6c3ab4e828fb40d6e05967b7aac9338	1.11E-17	k__Bacteria; p__Firmicutes; c__Clostridia; o__Clostridiales; f__Lachnospiraceae; g__Blautia; s__

Supplementary Table2. Sheet2. Key taxa in Bantu-Human identified based on the network hub score.

ASV	Hub Score	Taxa
X0b24831e597f08faedac938088911adb	1	k__Fungi;p__Ascomycota;c__Eurotiomycetes;o__Eurotiales;f__Aspergillaceae;g__Aspergillus;s__Aspergillus_penicillioides
X7d2997b35503bc54e9ef5a63975fed39	0.292390685	k__Fungi;p__Basidiomycota;c__Tremellomycetes;o__Trichosporonales;f__Trichosporonaceae;g__Trichosporon;s__Trichosporon_asahii
X5ef58296652d5359831628286945e59f	0.275505639	k__Fungi;p__Ascomycota;c__Saccharomycetes;o__Saccharomycetales
b2c9343b51ebe2282da3eda2fafb8693	0.244533307	k__Bacteria; p__Firmicutes; c__Clostridia; o__Clostridiales; f__Lachnospiraceae; g__Oribacterium; s__
X2ed442f2b4b06a701ea21c178567a128	0.198781978	k__Fungi;p__Ascomycota;c__Eurotiomycetes;o__Eurotiales;f__Aspergillaceae;g__Aspergillus;s__unidentified
X7a6a394fe7285b50ee54b56f316a3944	0.176548051	k__Bacteria; p__Bacteroidetes; c__Bacteroidia; o__Bacteroidales; f__Rikenellaceae; g__; s__
X43119e5f1eb41e9ee1776d7a6504126f	0.138414168	k__Bacteria; p__Firmicutes; c__Clostridia; o__Clostridiales; f__; g__; s__
X9272b77aeff548a2efc2b1917e111018	0.138414168	k__Bacteria; p__Firmicutes; c__Clostridia; o__Clostridiales; f__Ruminococcaceae; g__; s__
fe095885b690e99fffca72514e3fcef9	0.138414168	k__Bacteria; p__Firmicutes; c__Clostridia; o__Clostridiales; f__; g__; s__
b53e06f05e66b10fae9bf6eb67365f0e	0.138414168	k__Bacteria; p__Bacteroidetes; c__Bacteroidia; o__Bacteroidales; f__Prevotellaceae; g__Prevotella; s__stercorea
a2217cc8df6a4c209aee7f31a304c2b0	0.138414168	k__Bacteria; p__Firmicutes; c__Clostridia; o__Clostridiales; f__Ruminococcaceae; g__; s__
X54175402c30892510113a18348858786	0.138414168	k__Bacteria; p__Firmicutes; c__Erysipelotrichi; o__Erysipelotrichales; f__Erysipelotrichaceae; g__; s__
c0a8f5074b73b0648d1a21b1f3d8ffaf	0.138414168	k__Bacteria; p__Firmicutes; c__Clostridia; o__Clostridiales; f__Clostridiaceae; g__02d06; s__
deb90060cd7e33d6b3e583a968e4a11e	0.138414168	k__Bacteria; p__Bacteroidetes; c__Bacteroidia; o__Bacteroidales; f__Prevotellaceae; g__Prevotella; s__copri
X4bd01f90398375480101204bbcdd2ceb	0.040471013	k__Bacteria; p__Firmicutes; c__Clostridia; o__Clostridiales; f__Ruminococcaceae
f55d22a8e61d493b7f4c5e3f09326add	0.040471013	k__Bacteria; p__Firmicutes; c__Clostridia; o__Clostridiales; f__Lachnospiraceae; g__Butyrivibrio; s__crossotus
a0d16414f352e45bb94592a4cfdd6c37	0.040471013	k__Bacteria; p__Cyanobacteria; c__4C0d-2; o__YS2; f__; g__; s__
X6c324279c1fc2965e594486ac26b0548	0.040471013	k__Bacteria; p__Firmicutes; c__Clostridia; o__Clostridiales; f__Lachnospiraceae; g__Coprococcus; s__eutactus
X96cf4779be686036f92ee15e58b72d52	0.040471013	k__Bacteria; p__Firmicutes; c__Clostridia; o__Clostridiales; f__Veillonellaceae; g__Phascolarctobacterium; s__
b0b24b383b2d7d47b8858b31c76b40ee	0.031769773	k__Bacteria; p__Firmicutes; c__Clostridia; o__Clostridiales; f__Ruminococcaceae; g__; s__
X87f1f89372c1542c10504ef404c7ba31	0.030744906	k__Fungi;p__Ascomycota;c__Saccharomycetes;o__Saccharomycetales
c56fc2972887d4e6f0cadcd072640003	0.027514242	k__Bacteria; p__Firmicutes; c__Clostridia; o__Clostridiales; f__Ruminococcaceae; g__; s__
X8f27e96468755ca144a3b4edca1d4c4e	0.0108459	k__Fungi;p__Basidiomycota;c__Tremellomycetes;o__Trichosporonales;f__Trichosporonaceae;g__Trichosporon
X6aed27fe0a1493fe286a0d9dbd827347	0.006883406	k__Fungi;p__Ascomycota;c__Saccharomycetes;o__Saccharomycetales
X93f0debe0549ef4b266405e4e356d8f1	0.006709518	k__Bacteria; p__Actinobacteria; c__Actinobacteria; o__Actinomycetales; f__Corynebacteriaceae; g__Corynebacterium
ec6732c2e0d4cf64b3d0350e7fe3defb	0.004255531	k__Bacteria; p__Firmicutes; c__Clostridia; o__Clostridiales; f__Lachnospiraceae; g__Roseburia
a1c8a4289a5c93d48a8c7cc17815ba8a	0.004255531	k__Bacteria; p__Firmicutes; c__Erysipelotrichi; o__Erysipelotrichales; f__Erysipelotrichaceae; g__Bulleidia; s__p-1630-c5

X274c6b6158831a35ed9277540b91787f	0.002398956	k__Fungi;p__Ascomycota;c__Eurotiomycetes;o__Eurotiales;f__Aspergillaceae;g__Aspergillus;s__unidentified
X3114d42cc9f862a2a18d7e5efde7fa9b	0.001833276	k__Bacteria; p__Actinobacteria; c__Coriobacteriia; o__Coriobacteriales; f__Coriobacteriaceae; g__Slackia; s__
X2e4f2b53b856c4def6d021d01f5abb70	0.001833276	k__Bacteria; p__Firmicutes; c__Clostridia; o__Clostridiales; f__Lachnospiraceae; g__[Ruminococcus]; s__gnavus
X26b0fd3cb46859713999bbf58faea436	0.001697068	k__Archaea; p__Euryarchaeota; c__Thermoplasmata; o__E2; f__[Methanomassiliicoccaceae]; g__vadinCA11; s__
X86d2b555791a721b710afe3154c88b62	0.001501226	k__Bacteria; p__Firmicutes; c__Clostridia; o__Clostridiales; f__; g__; s__
X5e6805e860c751a55bd7ce70ca4b7534	0.001501226	k__Bacteria; p__Firmicutes; c__Clostridia; o__Clostridiales; f__Ruminococcaceae; g__; s__
fb428d07d03e32be0b85eacbed10df0a	0.001501226	k__Bacteria; p__Firmicutes; c__Clostridia; o__Clostridiales; f__Lachnospiraceae; g__Coprococcus; s__eutactus
X40adacc1c9e72c3b716624f295496e6c	0.001414899	k__Fungi;p__Ascomycota;c__Saccharomycetes;o__Saccharomycetales; f__Pichiaceae;g__Pichia;s__Pichia_norvegensis
bff475e3d60f57d6e4fbe246643e89de	0.000952761	k__Bacteria; p__Firmicutes; c__Clostridia; o__Clostridiales; f__Ruminococcaceae; g__; s__
X6348584315770e0d041c8543e15394f1	0.000952761	k__Bacteria; p__Firmicutes; c__Clostridia; o__Clostridiales; f__Ruminococcaceae
d084475ce17d67557615ecfb105797ab	0.000952761	k__Bacteria; p__Firmicutes; c__Clostridia; o__Clostridiales; f__Ruminococcaceae; g__; s__
X5654714fd17021f92582b02756943a86	0.000952761	k__Bacteria; p__Firmicutes; c__Clostridia; o__Clostridiales; f__; g__; s__
b7bb03f41b2044386d4c609b1085f722	0.000247956	k__Bacteria; p__Verrucomicrobia; c__Verruco-5; o__WCHB1-41; f__RFP12; g__; s__
X4f480409cf34da6fb985eaa1cf33a65b	0.000217501	k__Bacteria; p__Firmicutes; c__Clostridia; o__Clostridiales; f__Lachnospiraceae
X2343d35d2595e2249b6b0788c291563a	0.000198116	k__Fungi;p__Ascomycota;c__Saccharomycetes;o__Saccharomycetales
X114a0bce87e416d7710a2ea64c381af6	0.000178388	k__Fungi;p__Ascomycota;c__Eurotiomycetes;o__Eurotiales;f__Aspergillaceae;g__Aspergillus;s__unidentified
X93b258110943cf1f460fcc29f9914873	0.000156478	k__Fungi
X923fed1e3a5c67f886eb287b585a924a	2.74E-05	k__Bacteria; p__Firmicutes; c__Clostridia; o__Clostridiales; f__Lachnospiraceae; g__Coprococcus; s__
X545f2b8b71aad3c042a8f20fe0e25df2	2.74E-05	k__Bacteria; p__Firmicutes; c__Clostridia; o__Clostridiales; f__Ruminococcaceae; g__Faecalibacterium; s__prausnitzii
fea7f04af92d6da8eef36144b3589ad9	2.47E-05	k__Bacteria; p__Bacteroidetes; c__Bacteroidia; o__Bacteroidales; f__Porphyromonadaceae; g__Parabacteroides; s__
f663dd604f793f22ce20452b09494608	2.17E-05	k__Bacteria; p__Firmicutes; c__Clostridia; o__Clostridiales; f__Ruminococcaceae; g__; s__
X9e42f44aaff2b3eeb6336925728fd29f	1.63E-17	k__Fungi;p__Ascomycota;c__Saccharomycetes;o__Saccharomycetales
d61afa93b12293a3087aa4f0f2d73164	1.63E-17	k__Fungi;p__Mucoromycota;c__Mucoromycetes;o__Mucorales;f__Rhizopodaceae;g__Rhizopus;s__Rhizopus_arrhizus
c48070f3061b086b60ff32f77e0002fa	3.65E-18	k__Bacteria; p__Firmicutes; c__Clostridia; o__Clostridiales; f__Ruminococcaceae; g__Faecalibacterium; s__prausnitzii
c66ec67719e54d2cb0afc3154b3c23af	3.65E-18	k__Bacteria; p__Firmicutes; c__Clostridia; o__Clostridiales; f__; g__; s__
c3337d877a02fb500fc37be8aa4fd234	3.65E-18	k__Bacteria; p__Firmicutes; c__Clostridia; o__Clostridiales; f__[Mogibacteriaceae]; g__; s__
X0501d0de95c0ade37e2eb0c56cf6df87	3.65E-18	k__Fungi
X45764da73ff78cb08974dcef2aeff320	3.65E-18	k__Fungi;p__Ascomycota;c__Saccharomycetes;o__Saccharomycetales; f__Saccharomycetaceae;g__Saccharomyces
cbff7dcda657642a9c4e949a23aadfe3	3.65E-18	k__Fungi;p__Ascomycota;c__Eurotiomycetes;o__Eurotiales;f__Aspergillaceae;g__Aspergillus;s__Aspergillus_brasiliensis

X35cbb315e019e0a8768d4b73862df415	2.30E-18	k__Bacteria; p__Actinobacteria; c__Coriobacteriia; o__Coriobacteriales; f__Coriobacteriaceae; g__Collinsella; s__aerofaciens
X288359d621a383c1e46f0c0418660242	1.15E-18	k__Fungi;p__Ascomycota;c__Dothideomycetes;o__Botryosphaeriales;f__Botryosphaeriaceae;g__Lasiodiplodia
X980d3ec25be6051aca44c0a6c4fbd6bb	1.15E-18	k__Fungi

Supplementary Table2. Sheet3. Key taxa in Captive Chimps-Ostrava identified based on the network hub score.

ASV	HubScore	Taxa
X842b231d632137e161df6d969797c2b9	1	k__Fungi
X429cf0d4d6dbee8b629acba2252d194a	0.508763197	k__Fungi;p__Ascomycota;c__Eurotiomycetes;o__Eurotiales;f__Aspergillaceae;g__Penicillium;s__unidentified
b54a583c333aa96b10152106510ca6cd	0.320856626	k__Fungi
X799cc660a43d6ee89a7ad4907111c073	0.307889867	k__Fungi;p__unidentified;c__unidentified;o__unidentified;f__unidentified;g__unidentified;s__unidentified
X64efc7cc8a98397ed156bb7deb9ea0d1	0.30356223	k__Fungi
X022e203c0237562b1af030c2cda7cfd9	0.284171427	k__Fungi;p__unidentified;c__unidentified;o__unidentified;f__unidentified;g__unidentified;s__unidentified
cd27dfa017d816443e5e48c2d8993e06	0.2593872	k__Fungi;p__Ascomycota;c__Sordariomycetes;o__Microascales;f__Microascaceae;g__unidentified;s__unidentified
X9f07dabd22066fabc4620a2ef42400d3	0.170561371	k__Fungi;p__Ascomycota;c__Dothideomycetes;o__Capnodiales
X37f004671c936285c26471120596b2f5	0.166014189	k__Bacteria; p__Firmicutes; c__Clostridia; o__Clostridiales; f__Clostridiaceae; g__Sarcina; s__
X35cbb315e019e0a8768d4b73862df415	0.141057552	k__Bacteria; p__Actinobacteria; c__Coriobacteriia; o__Coriobacteriales; f__Coriobacteriaceae; g__Collinsella; s__aerofaciens
X3a0c5873e8683c4c3cec68faa75b3bb0	0.141057552	k__Bacteria; p__Proteobacteria; c__Gammaproteobacteria; o__Aeromonadales; f__Succinivibrionaceae; g__Ruminobacter; s__
fcfe725f65877522a4a8db1e89380d7e	0.130361494	k__Bacteria
X94a302cc7a648f23c27353f085b24e06	0.126766203	k__Bacteria; p__Firmicutes; c__Clostridia; o__Clostridiales; f__Veillonellaceae; g__Anaerovibrio; s__
b0b24b383b2d7d47b8858b31c76b40ee	0.119521047	k__Bacteria; p__Firmicutes; c__Clostridia; o__Clostridiales; f__Ruminococcaceae; g__; s__
X6128c061f0201a07dcabe9199a0ba49e	0.119521047	k__Bacteria; p__Spirochaetes; c__Spirochaetes; o__Spirochaetales; f__Spirochaetaceae; g__Treponema; s__
c04c87ff72eb8ad4ec07894d5b0cf2a7	0.117353546	Unassigned
aae4c4ed528ae16ec417df816699cfdb	0.117353546	k__Bacteria; p__Firmicutes; c__Clostridia; o__Clostridiales; f__Ruminococcaceae; g__; s__
X4e2409473c1c1d45bc74650c14680360	0.105657624	k__Fungi;p__unidentified;c__unidentified;o__unidentified;f__unidentified;g__unidentified;s__unidentified

X159f25ee8dfdd348c2ffee847ac7a30c	0.091384642	k__Bacteria; p__Planctomycetes; c__Planctomycetia; o__Pirellulales; f__Pirellulaceae; g__; s__
a8b4b0d58275413b83f3fe9986834bb9	0.091384642	k__Bacteria; p__Firmicutes; c__Clostridia; o__Clostridiales; f__Ruminococcaceae; g__; s__
a63e647026935bffac1572dc6237a5d7	0.091384642	k__Bacteria; p__Firmicutes; c__Clostridia; o__Clostridiales; f__Ruminococcaceae; g__; s__
deb90060cd7e33d6b3e583a968e4a11e	0.091384642	k__Bacteria; p__Bacteroidetes; c__Bacteroidia; o__Bacteroidales; f__Prevotellaceae; g__Prevotella; s__copri
X3114d42cc9f862a2a18d7e5efde7fa9b	0.091384642	k__Bacteria; p__Actinobacteria; c__Coriobacteriia; o__Coriobacteriales; f__Coriobacteriaceae; g__Slackia; s__
c48070f3061b086b60ff32f77e0002fa	0.091384642	k__Bacteria; p__Firmicutes; c__Clostridia; o__Clostridiales; f__Ruminococcaceae; g__Faecalibacterium; s__prausnitzii
c6c3ab4e828fb40d6e05967b7aac9338	0.091384642	k__Bacteria; p__Firmicutes; c__Clostridia; o__Clostridiales; f__Lachnospiraceae; g__Blautia; s__
X32fdd0ac9d955cf84083aeff0d0eead6	0.079281986	k__Fungi;p__unidentified;c__unidentified;o__unidentified;f__unidentified;g__unidentified;s__unidentified
d008df29aded33e62b0a9f066fffba9a	0.078942889	k__Bacteria; p__Spirochaetes; c__Spirochaetes; o__Spirochaetales; f__Spirochaetaceae; g__Treponema; s__
X67291fcee109ab8a23a8be4925084a4a	0.074234068	k__Bacteria
b4c0e06c3ac9a3a297c9c02a2a95ece1	0.074234068	k__Bacteria; p__Firmicutes; c__Clostridia; o__Clostridiales; f__Lachnospiraceae; g__Coprococcus; s__
e92159c91aeb204e77ba7a625416173e	0.074234068	k__Bacteria; p__Spirochaetes; c__Spirochaetes; o__Spirochaetales; f__Spirochaetaceae; g__Treponema; s__
X673c876927c20776ed0bdc60805ab4c7	0.074234068	k__Bacteria; p__Bacteroidetes; c__Bacteroidia; o__Bacteroidales; f__S24-7; g__; s__
X66deff5f5235adafa76ebd45e526df43	0.074234068	k__Bacteria; p__Firmicutes; c__Erysipelotrichi; o__Erysipelotrichales; f__Erysipelotrichaceae; g__Allobaculum; s__
a0d16414f352e45bb94592a4cfdd6c37	0.074234068	k__Bacteria; p__Cyanobacteria; c__4C0d-2; o__YS2; f__; g__; s__
X0b85c3b6bae1477ecc014b93711dca4d	0.051527485	k__Fungi;p__Basidiomycota;c__Tremellomycetes;o__Trichosporonales;f__Trichosporonaceae;g__Cutaneotrichosporon;s__Cutaneotrichosporon_smithiae
X74a6b2d8faae4662532b0e4123964dac	0.048952892	k__Bacteria; p__Tenericutes; c__Mollicutes; o__RF39; f__; g__; s__
cc1cf7a701a618852f5e2f3da57a6277	0.046493142	k__Bacteria; p__Firmicutes; c__Clostridia; o__Clostridiales; f__Ruminococcaceae; g__Ruminococcus; s__flavefaciens
X8adadd953ff6f3831d8492aaecdbe713	0.046493142	k__Bacteria; p__Proteobacteria; c__Gammaproteobacteria; o__Aeromonadales; f__Succinivibrionaceae; g__Succinivibrio; s__

X24284cec3ba6a25cbdeb7322cc8450f8	0.046493142	k__Bacteria; p__Verrucomicrobia; c__Verruco-5; o__WCHB1-41; f__RFP12; g__; s__
X6a8452da8f7a21e41e8b7b1f96c18f41	0.046493142	k__Bacteria; p__Cyanobacteria; c__4C0d-2; o__YS2; f__; g__; s__
X528aa0da258f5c8f78efa1027e6acbe8	0.044908058	k__Bacteria; p__Firmicutes; c__Clostridia; o__Clostridiales; f__Lachnospiraceae
X5e9a4e49c57a0f1457663cbec94ead42	0.044908058	k__Bacteria; p__Lentisphaerae; c__[Lentisphaeria]; o__Z20; f__R4-45B; g__; s__
X880ff2cf5f6257cc1b867cd60fc8d0c6	0.044908058	k__Bacteria; p__Proteobacteria; c__Alphaproteobacteria; o__Rickettsiales; f__; g__; s__
X06ca1d8e7b0adc837e3a73dc4938a0b7	0.044908058	k__Bacteria; p__Firmicutes; c__Clostridia; o__Clostridiales; f__Ruminococcaceae; g__Ruminococcus; s__
X9639a3291729a3758207b47715d9205f	0.038976852	k__Bacteria; p__Firmicutes; c__Clostridia; o__Clostridiales; f__Ruminococcaceae; g__Gemmiger; s__formicilis
X0a7fe3595e40caeafecb26f55ecf526d	0.032138821	k__Fungi;p__Ascomycota;c__Dothideomycetes;o__Capnodiales;f__Cladosporiaceae;g__Cladosporium
f7e2b0a56c65be92cbe4e45ec5be863b	0.029321368	k__Bacteria; p__Firmicutes; c__Clostridia; o__Clostridiales; f__Christensenellaceae; g__; s__
X39993a34fd31592b41224d9e4f81ebb7	0.029321368	k__Bacteria; p__Verrucomicrobia; c__Verruco-5; o__WCHB1-41; f__RFP12; g__; s__
b7bb03f41b2044386d4c609b1085f722	0.029321368	k__Bacteria; p__Verrucomicrobia; c__Verruco-5; o__WCHB1-41; f__RFP12; g__; s__
X4109fbbe9cd1407916d6f86e0fc3038d	0.029321368	k__Bacteria; p__Firmicutes; c__Clostridia; o__Clostridiales; f__; g__; s__
ef302df176b1e1e14c64f4c3ccc6a54a	0.029321368	k__Bacteria; p__Verrucomicrobia; c__Verruco-5; o__WCHB1-41; f__RFP12; g__; s__
X61535a40adb8221483821e3984d0b1ac	0.029321368	k__Bacteria; p__Firmicutes; c__Clostridia; o__Clostridiales; f__Lachnospiraceae
X3e77391a15f92a914190ae7e675bafd9	0.029321368	k__Bacteria; p__Firmicutes; c__Clostridia; o__Clostridiales; f__Lachnospiraceae; g__; s__
X86ff0efd7fd88b82809500c90c4a7832	0.029321368	k__Bacteria; p__Firmicutes; c__Clostridia; o__Clostridiales; f__Ruminococcaceae; g__Faecalibacterium; s__prausnitzii
X7857dc942cdb7fe42401378e8b21a98b	0.028136405	k__Bacteria; p__Elusimicrobia; c__Elusimicrobia; o__Elusimicrobiales; f__Elusimicrobiaceae; g__; s__
X0dcf5023e96fb8175d7bf02d900c9dd1	0.027740926	k__Bacteria; p__Firmicutes; c__Clostridia; o__Clostridiales; f__Ruminococcaceae; g__; s__
X2543dd8ec108ba946069649982de4e33	0.026916439	k__Fungi;p__Ascomycota;c__Eurotiomycetes;o__Eurotiales;f__Aspergillaceae;g__Penicillium

cc7b0c4fe7613151aee46e9be3ea14a9	0.02370400 6	k__Bacteria; p__Firmicutes; c__Clostridia; o__Clostridiales; f__Ruminococcaceae; g__Oscillospira; s__
X56df15deaff211010e1470c27a31477f	0.02370400 6	k__Bacteria; p__Firmicutes; c__Clostridia; o__Clostridiales; f__; g__; s__
e865f29a716e8d51f83931befa951240	0.02370400 6	k__Bacteria; p__Firmicutes; c__Erysipelotrichi; o__Erysipelotrichales; f__Erysipelotrichaceae; g__; s__
ff1b8c1c7f48d303e4bbc51cf5089e56	0.02370400 6	k__Bacteria; p__Firmicutes; c__Erysipelotrichi; o__Erysipelotrichales; f__Erysipelotrichaceae; g__[Eubacterium]; s__biforme
a1c8a4289a5c93d48a8c7cc17815ba8a	0.02370400 6	k__Bacteria; p__Firmicutes; c__Erysipelotrichi; o__Erysipelotrichales; f__Erysipelotrichaceae; g__Bulleidia; s__p-1630-c5
f4f5f27b452dc435c90e33bc62500294	0.02370400 6	k__Bacteria; p__Bacteroidetes; c__Bacteroidia; o__Bacteroidales; f__; g__; s__
X8f7e3b18e53065a81eacfdf0fb13c0b1	0.02370400 6	k__Bacteria; p__Bacteroidetes; c__Bacteroidia; o__Bacteroidales; f__; g__; s__
X54947f672cc093728ece078418837f82	0.02370400 6	k__Bacteria; p__Spirochaetes; c__Spirochaetes; o__Sphaerochaetales; f__Sphaerochaetaceae; g__Sphaerochaeta; s__
X6348584315770e0d041c8543e15394f1	0.01852368 4	k__Bacteria; p__Firmicutes; c__Clostridia; o__Clostridiales; f__Ruminococcaceae
X8ebc5c1f92c8c0fd263bafc9882304a2	0.01852368 4	k__Bacteria; p__Bacteroidetes; c__Bacteroidia; o__Bacteroidales; f__; g__; s__
X17b93228ec8a29b236559a1b56a71c26	0.01558669	k__Bacteria; p__Bacteroidetes; c__Bacteroidia; o__Bacteroidales; f__; g__; s__
X4f73103c89da8800036a75ac5df9fb74	0.01558669	k__Bacteria; p__Firmicutes; c__Clostridia; o__Clostridiales; f__Lachnospiraceae; g__; s__
X65990c74559e4adfaa672a884ad7d464	0.01558669	k__Bacteria; p__Firmicutes; c__Clostridia; o__Clostridiales; f__Lachnospiraceae; g__Coprococcus; s__catus
b726627e18f29e0da5289f81ca294f92	0.01558669	k__Bacteria; p__Firmicutes; c__Clostridia; o__Clostridiales; f__Ruminococcaceae
X3219af75ee8056ed06ba81e873b0d0cb	0.01558669	k__Bacteria; p__Firmicutes; c__Clostridia; o__Clostridiales; f__Ruminococcaceae; g__Oscillospira; s__
X3d6e1834125d9ab26f23a1bf8db2195f	0.01558669	k__Bacteria; p__Proteobacteria; c__Deltaproteobacteria; o__GMD14H09; f__; g__; s__
X274d44a105684512d6df0a983a5b6663	0.01259247 9	k__Bacteria; p__Firmicutes; c__Clostridia; o__Clostridiales; f__Clostridiaceae; g__02d06; s__
X935a26480fd0df674c49c7a7f219c786	0.01128089	k__Fungi;p__Ascomycota;c__Sordariomycetes;o__Sordariales;f__Sordariaceae;g__Sordaria;s__Sordaria_fimicola
X09887403d0ff191779ed75738262917c	0.01022855 5	k__Bacteria; p__Spirochaetes; c__Spirochaetes; o__Spirochaetales; f__Spirochaetaceae; g__Treponema; s__

e68c1bb6fac22947077f421d9a5bf031	0.009539107	k__Fungi;p__unidentified;c__unidentified;o__unidentified;f__unidentified;g__unidentified;s__unidentified
a9e2afd4297fd011649a216315249232	0.009147784	k__Bacteria; p__Bacteroidetes; c__Bacteroidia; o__Bacteroidales; f__Prevotellaceae; g__; s__
X40a99f13336f2908c2101fada03e8925	0.008276056	k__Bacteria; p__Firmicutes; c__Clostridia; o__Clostridiales; f__Lachnospiraceae; g__Coprococcus; s__
b33432097ae173340229ec6cf0cb249c	0.00704295	k__Fungi
X9f5863a1c1d6034cff75066ead33e04e	0.006270976	k__Fungi;p__unidentified;c__unidentified;o__unidentified;f__unidentified;g__unidentified;s__unidentified
f5f5e0da89730462abaf6301a9557193	0.005352438	k__Bacteria; p__Firmicutes; c__Clostridia; o__Clostridiales; f__Ruminococcaceae; g__Faecalibacterium; s__prausnitzii
X45158693222383612f0b6b469c0be1fe	0.004708821	k__Bacteria; p__Bacteroidetes; c__Bacteroidia; o__Bacteroidales; f__[Paraprevotellaceae]; g__[Prevotella]; s__
X3e24620a7af3193ab178578867926be2	0.004708821	k__Bacteria; p__Firmicutes; c__Clostridia; o__Clostridiales; f__Peptostreptococcaceae
X54239c6454a6a3d3a48e90a79744afb7	0.002936995	k__Bacteria; p__Firmicutes; c__Clostridia; o__Clostridiales; f__Ruminococcaceae
cb908ae7d11351c78c0e4ce0d849c744	0.002936995	k__Bacteria; p__Firmicutes; c__Clostridia; o__Clostridiales; f__Ruminococcaceae; g__; s__
a2d300d1e9c621dbc5239cc092d433c3	0.002936995	k__Bacteria; p__Firmicutes; c__Clostridia; o__Clostridiales; f__; g__; s__
ed6b49ec452f003a91e2b30eb15cdf6c	0.001838712	k__Fungi;p__Ascomycota;c__Eurotiomycetes;o__Eurotiales;f__Aspergillaceae;g__Penicillium;s__unidentified
d76d59ec71de0e3b22da0c9cd564d41a	0.001515345	k__Bacteria; p__Firmicutes; c__Clostridia; o__Clostridiales; f__Lachnospiraceae
X96cf4779be686036f92ee15e58b72d52	0.001515345	k__Bacteria; p__Firmicutes; c__Clostridia; o__Clostridiales; f__Veillonellaceae; g__Phascolarctobacterium; s__
X89ddf52e50218f8dff5d6e93e6e65f0a	0.001515345	k__Bacteria; p__Firmicutes; c__Clostridia; o__Clostridiales; f__Ruminococcaceae; g__; s__
X4102c0c9284b080a36ae312a7b497915	0.001039758	k__Bacteria; p__Proteobacteria; c__Deltaproteobacteria; o__Desulfovibrionales; f__Desulfovibrionaceae; g__Desulfovibrio; s__
ff6fd693d1740f436ec1317219115750	0.0010309	k__Bacteria; p__Bacteroidetes; c__Bacteroidia; o__Bacteroidales; f__Porphyromonadaceae; g__Parabacteroides; s__
X59777186ad2e0947e97615b5d6225136	0.0010309	k__Bacteria; p__Firmicutes; c__Clostridia; o__Clostridiales; f__Ruminococcaceae; g__Faecalibacterium; s__prausnitzii
X01e2df15a7c13fe503196a9b9ebff980	0.0010309	k__Bacteria; p__Tenericutes; c__Mollicutes; o__Anaeroplasmatales; f__Anaeroplasmataceae; g__Anaeroplasma; s__

X6077859e3a7ca4807e8d3b9ec2dfe082	0.000871728	k__Bacteria; p__Firmicutes; c__Clostridia; o__Clostridiales; f__; g__; s__
X2c56af9b2c17b64fd4c7a2fa95fcf197	0.000871728	k__Bacteria; p__Spirochaetes; c__Spirochaetes; o__Sphaerochaetales; f__Sphaerochaetaceae; g__Sphaerochaeta; s__
f499d206798403d58d114c5d0ed6c832	0.000871728	k__Bacteria; p__Cyanobacteria; c__4C0d-2; o__YS2; f__; g__; s__
X11e80f6b0f99ffd9c86001e7dcc2472f	0.000811648	k__Bacteria; p__Firmicutes; c__Erysipelotrichi; o__Erysipelotrichales; f__Erysipelotrichaceae; g__RFN20; s__
dfe6c643b7485f470e3cc6d40221a8df	0.000811648	k__Bacteria; p__Firmicutes; c__Clostridia; o__Clostridiales; f__Lachnospiraceae
X20a68b281025ba8f8addfffca22b306a	0.000643618	k__Bacteria; p__Bacteroidetes; c__Bacteroidia; o__Bacteroidales; f__Prevotellaceae; g__Prevotella; s__
fe6fc175855fb067abd854c21279b845	0.000643618	k__Bacteria; p__Firmicutes; c__Clostridia; o__Clostridiales; f__Ruminococcaceae
X3b3f9c7bb2ca421e0c31645737223b39	0.00060339	k__Bacteria; p__Verrucomicrobia; c__Verruco-5; o__WCHB1-41; f__RFP12; g__; s__
c95b6b8f54f860200da7644517333239	0.000573071	k__Bacteria; p__Bacteroidetes; c__Bacteroidia; o__Bacteroidales; f__[Paraprevotellaceae]; g__CF231; s__
X1a8ad95a0274f9686ca16a69ab153918	0.00033177	k__Fungi;p__unidentified;c__unidentified;o__unidentified;f__unidentified;g__unidentified;s__unidentified
X54a2bfb0a96adc52c4ccca239ad01466	0.00017692	k__Bacteria; p__Firmicutes; c__Clostridia; o__Clostridiales; f__Ruminococcaceae; g__Ruminococcus; s__
X9acb555d3c5dd77e9035a661e7ad1bb6	0.00016803	k__Bacteria; p__Firmicutes; c__Clostridia; o__Clostridiales; f__Clostridiaceae; g__Clostridium; s__celatum
X3afd2ff14c94969b67b49b45adafb760	0.00016803	k__Bacteria; p__Spirochaetes; c__Spirochaetes; o__Spirochaetales; f__Spirochaetaceae; g__Treponema; s__
e432a172c15b9c449cea7ac7828eba95	0.00016803	k__Bacteria; p__Firmicutes; c__Bacilli; o__Lactobacillales; f__Lactobacillaceae; g__Lactobacillus; s__
X2ec8a70980fe98f5039f02c624042c91	9.73E-05	k__Fungi;p__unidentified;c__unidentified;o__unidentified;f__unidentified;g__unidentified;s__unidentified

Supplementary Table2. Sheet4. Key taxa in Captive Western Lowland Gorilla identified based on the network hub score.

ASV	HubScore	Taxa
cccb119ab38bd8544732a0d525c9125d	1	k__Fungi
X9aeaf3016bff201f723b2c61aed4d73a	0.8339941 8	k__Fungi
X2fddd02a57f8ceb4c8ae4f7e6ee0040d	0.6990463 4	k__Fungi;p__unidentified;c__unidentified;o__unidentified;f__unidentified;g__unidentified;s__unidentified
ef974ef33a37e04588c24fb83f533d4e	0.5720550 7	k__Fungi;p__unidentified;c__unidentified;o__unidentified;f__unidentified;g__unidentified;s__unidentified
eae7022c2729b4b1ea7a62abe468e799	0.5594086 8	k__Fungi
X4508f7952b28669ca020b60e95bec469	0.3994286 9	k__Fungi;p__Ascomycota;c__Sordariomycetes;o__Sordariales;f__Lasiosphaeriaceae;g__Podospora;s__Podospora_longicollis
X170b12863014c91d2a9b791d6540ef77	0.3162999 5	k__Fungi
X96e7e25cdc80521a63c692e16f047093	0.2898528	k__Fungi;p__unidentified;c__unidentified;o__unidentified;f__unidentified;g__unidentified;s__unidentified
X31e61e702cd999e03597ff23fcb3aa64	0.2750148 7	k__Bacteria; p__Lentisphaerae; c__[Lentisphaeria]; o__Z20; f__R4-45B; g__; s__
ac7fe0192cd9a64714d246e95fe0bb87	0.2712932 2	k__Fungi
a00b80d9e17196f223b318053de2fc63	0.2051190 8	k__Fungi;p__unidentified;c__unidentified;o__unidentified;f__unidentified;g__unidentified;s__unidentified
X536e8f9e5358ea477cfa84657d69043a	0.1669647 1	k__Bacteria; p__Bacteroidetes; c__Bacteroidia; o__Bacteroidales; f__RF16; g__; s__
a7571cd4237c0aba55f06d43296a3eb5	0.1442226 2	k__Bacteria; p__Firmicutes; c__Clostridia; o__Clostridiales; f__Clostridiaceae; g__Clostridium; s__
X9272b77aeff548a2efc2b1917e111018	0.1442226 2	k__Bacteria; p__Firmicutes; c__Clostridia; o__Clostridiales; f__Ruminococcaceae; g__; s__
X2e7dd2d044c222cf34a93c75ce67eaaa	0.1442226 2	k__Bacteria; p__Spirochaetes; c__Spirochaetes; o__Spirochaetales; f__Spirochaetaceae; g__Treponema; s__
d68bf654aa3d8a13b2ed6ce7ff9e7826	0.1442226 2	k__Bacteria; p__Spirochaetes; c__Spirochaetes; o__Spirochaetales; f__Spirochaetaceae; g__Treponema; s__
cf5031364fd6f4fdbfccfb2fd88bd4f8	0.1442226 2	k__Bacteria; p__Firmicutes; c__Clostridia; o__Clostridiales; f__Ruminococcaceae; g__Oscillospira; s__
X72fda00662eaa167bd1c082d0302f709	0.1442226 2	k__Bacteria; p__Firmicutes; c__Clostridia; o__Clostridiales; f__; g__; s__

X3127bd317be815d64dff5a7895408312	0.1442226 2	k__Bacteria; p__Firmicutes; c__Clostridia; o__Clostridiales; f__Ruminococcaceae; g__; s__
e2f5328726e4b0bb7e365a0c1d9e85b7	0.1442226 2	k__Bacteria; p__Firmicutes; c__Clostridia; o__Clostridiales; f__Ruminococcaceae; g__; s__
X7d6b17e49613cdad1210312654549570	0.1213582 2	k__Bacteria; p__Spirochaetes; c__Spirochaetes; o__Sphaerochaetales; f__Sphaerochaetaceae; g__Sphaerochaeta; s__
X612c1a863fb0f34f5edbccffa3a9c73a	0.1206192 9	k__Archaea; p__Euryarchaeota; c__Thermoplasmata; o__E2; f__[Methanomassiliicoccaceae]; g__vadinCA11; s__
ffe811c34c6c002613241c56ed0322a0	0.1194799 5	k__Bacteria; p__Firmicutes; c__Clostridia; o__Clostridiales; f__Lachnospiraceae
X5e6805e860c751a55bd7ce70ca4b7534	0.1194799 5	k__Bacteria; p__Firmicutes; c__Clostridia; o__Clostridiales; f__Ruminococcaceae; g__; s__
X0c8c796cb9568bb42a42b207bc78a7e8	0.1176270 5	k__Fungi
X524145c68413c96798867bbdb482bea8	0.1044213 2	k__Bacteria; p__Bacteroidetes; c__Bacteroidia; o__Bacteroidales; f__; g__; s__
X794cc1c6386320420a2c2ea76eedaaf8	0.1044213 2	k__Bacteria; p__Firmicutes; c__Clostridia; o__Clostridiales; f__Ruminococcaceae; g__; s__
d6431d139bc9a31eddd4c56368217b4d	0.0903921	k__Bacteria; p__Firmicutes; c__Clostridia; o__Clostridiales; f__Christensenellaceae; g__; s__
d7907139a74128fa9785178a7a3e4421	0.0895074 1	k__Bacteria; p__Verrucomicrobia; c__Verruco-5; o__WCHB1-41; f__RFP12; g__; s__
cc1cf7a701a618852f5e2f3da57a6277	0.0872860 6	k__Bacteria; p__Firmicutes; c__Clostridia; o__Clostridiales; f__Ruminococcaceae; g__Ruminococcus; s__flavefaciens
X0088fdc9bf1dfa441e53e964ad7591a5	0.0856733 6	k__Bacteria; p__Firmicutes; c__Clostridia; o__Clostridiales; f__Ruminococcaceae; g__; s__
f6be8fd3434273e20c0b5a1461ee3c85	0.0839476 8	k__Bacteria; p__Firmicutes; c__Clostridia; o__Clostridiales; f__; g__; s__
X59b961d6271b069ab40420ecdbe35e0c	0.0809248 8	k__Bacteria; p__Firmicutes; c__Clostridia; o__Clostridiales; f__Ruminococcaceae; g__; s__
bd85ca4d76167e7ecf3ec65f8701e35c	0.0809248 8	k__Bacteria; p__Firmicutes; c__Erysipelotrichi; o__Erysipelotrichales; f__Erysipelotrichaceae; g__RFN20; s__
X2c56af9b2c17b64fd4c7a2fa95fcf197	0.0800556 1	k__Bacteria; p__Spirochaetes; c__Spirochaetes; o__Sphaerochaetales; f__Sphaerochaetaceae; g__Sphaerochaeta; s__
X3cdea77d0433c3e929515bd898a9724a	0.0749455 9	k__Bacteria; p__Bacteroidetes; c__Bacteroidia; o__Bacteroidales; f__; g__; s__
c0241dff3c50c2bd8484d30accbbea2a	0.0749455 9	k__Bacteria; p__Cyanobacteria; c__4C0d-2; o__YS2; f__; g__; s__

X32fdd0ac9d955cf84083aeff0d0eead6	0.0743468 5	k__Fungi;p__unidentified;c__unidentified;o__unidentified;f__unidentified;g__unidentified;s__unidentified
X1e6f14b638e1593de746394f96eed0df	0.0720036 2	k__Bacteria; p__Firmicutes; c__Clostridia; o__Clostridiales; f__Ruminococcaceae; g__; s__
X8ec7ad4ccbf595f7af04d4cf4394e14d	0.0705538 9	k__Fungi;p__unidentified;c__unidentified;o__unidentified;f__unidentified;g__unidentified;s__unidentified
b73dfbea02dfca1e4ab401e53fc31ab5	0.0702268 6	k__Bacteria; p__Firmicutes; c__Clostridia; o__Clostridiales; f__Ruminococcaceae; g__; s__
b852420578e04f24dfef8591f9322eeb	0.0702268 6	k__Bacteria; p__Firmicutes; c__Clostridia; o__Clostridiales; f__Ruminococcaceae
a217b6092dfe383f3663e46a925ef893	0.0684387 5	k__Bacteria; p__Firmicutes; c__Clostridia; o__Clostridiales; f__; g__; s__
aa77ead1195463b9ccc93ecaa33bf6f4	0.0670831	k__Bacteria; p__Firmicutes; c__Clostridia; o__Clostridiales; f__Veillonellaceae; g__Schwartzia; s__
X847dd19dd00c43810f3051cf594a1a77	0.0644216 6	k__Bacteria
X66bf0cd4dc666eb2858567908e536a3e	0.0644216 6	k__Bacteria; p__Proteobacteria; c__Alphaproteobacteria; o__RF32; f__; g__; s__
bfd71e3ae2d65c810f5a8b922073d55a	0.0644216 6	k__Bacteria; p__Proteobacteria; c__Gammaproteobacteria; o__Aeromonadales; f__Succinivibrionaceae; g__; s__
bbf05181187cd72dd323041c57ba73f9	0.0644216 6	k__Bacteria; p__Tenericutes; c__RF3; o__ML615J-28; f__; g__; s__
X961207bc9171c53851bf1ae389fdcb8f	0.0644216 6	k__Bacteria; p__Cyanobacteria; c__4C0d-2; o__YS2; f__; g__; s__
de538b4503aa6cb78336f2067daaed3c	0.0572771 6	k__Bacteria; p__Planctomycetes; c__Planctomycetia; o__Pirellulales; f__Pirellulaceae; g__; s__
X291e288a11df43742c359d0276e911c1	0.0569365 6	k__Bacteria; p__Firmicutes; c__Clostridia; o__Clostridiales; f__; g__; s__
X2986bbe7d293a27dd6204cde2ab28477	0.0569365 6	k__Bacteria; p__Firmicutes; c__Clostridia; o__Clostridiales; f__Lachnospiraceae; g__; s__
bb47077706877cba0ec7ac2f57d4dc87	0.0569365 6	k__Bacteria; p__Tenericutes; c__Mollicutes; o__RF39; f__; g__; s__
c48070f3061b086b60ff32f77e0002fa	0.0569365 6	k__Bacteria; p__Firmicutes; c__Clostridia; o__Clostridiales; f__Ruminococcaceae; g__Faecalibacterium; s__prausnitzii
X41a682ff5b44533c0912c7cd987419ff	0.0541918 1	k__Bacteria; p__Verrucomicrobia; c__Verruco-5; o__WCHB1-41; f__RFP12; g__; s__
d1720fa992a428f1a4a9fb8e436b7866	0.0530911 7	k__Bacteria; p__Proteobacteria; c__Betaproteobacteria; o__Burkholderiales; f__Alcaligenaceae; g__Sutterella; s__

X74b205364779788584cf822deb1615be	0.0529063 4	k__Archaea; p__Euryarchaeota; c__Thermoplasmata; o__E2; f__[Methanomassiliicoccaceae]; g__vadinCA11; s__
e6c95d620576f6503d607275d777bef1	0.0525870 8	k__Archaea; p__Euryarchaeota; c__Thermoplasmata; o__E2; f__[Methanomassiliicoccaceae]; g__; s__
ec3c4e6dd84442b5789313cc9d9e5cfb	0.0498598 4	k__Bacteria; p__Spirochaetes; c__Spirochaetes; o__Spirochaetales; f__Spirochaetaceae; g__Treponema; s__
a9e2afd4297fd011649a216315249232	0.0480173 5	k__Bacteria; p__Bacteroidetes; c__Bacteroidia; o__Bacteroidales; f__Prevotellaceae; g__; s__
X902dc2afbcc15736090e3fe703673268	0.0474847 6	k__Bacteria; p__Proteobacteria; c__Deltaproteobacteria; o__Desulfovibrionales; f__Desulfovibrionaceae; g__Bilophila; s__
X39d811658a63215ad320d73c5f0c0d16	0.0474847 6	k__Bacteria; p__Cyanobacteria; c__4C0d-2; o__YS2; f__; g__; s__
bd02f5c417cc8b1d74c3c5bdd030547f	0.0474847 6	k__Bacteria; p__Bacteroidetes; c__Bacteroidia; o__Bacteroidales; f__S24-7; g__; s__
X935a26480fd0df674c49c7a7f219c786	0.0470606 8	k__Fungi;p__Ascomycota;c__Sordariomycetes;o__Sordariales;f__Sordariaceae;g__Sordaria;s__Sordaria_fimicola
X76b256496b7923e3fe43c1e12fd04bea	0.0462090 6	k__Bacteria; p__Bacteroidetes; c__Bacteroidia; o__Bacteroidales; f__; g__; s__
X3ee0ff836431c7ba5a4b0106b4dd047f	0.0404803 2	k__Fungi
X207ae2c759c09be976bbb456d7d7eca6	0.0398013	k__Bacteria; p__Firmicutes; c__Clostridia; o__Clostridiales; f__[Mogibacteriaceae]; g__; s__
X10b0ccda77f21a69527f7596b15c1c23	0.0392681 3	k__Bacteria; p__Actinobacteria; c__Coriobacteriia; o__Coriobacteriales; f__Coriobacteriaceae; g__; s__
X37f004671c936285c26471120596b2f5	0.0387453 1	k__Bacteria; p__Firmicutes; c__Clostridia; o__Clostridiales; f__Clostridiaceae; g__Sarcina; s__
f90db7a7347641bf095b13079a732743	0.0365879 5	k__Bacteria; p__Cyanobacteria; c__4C0d-2; o__YS2; f__; g__; s__
X6cec9979c3e76d4316a289bf1792356f	0.0358679	k__Bacteria; p__Proteobacteria; c__Betaproteobacteria; o__Burkholderiales; f__Alcaligenaceae; g__Sutterella; s__
X7717c98118439762317647199c6005f7	0.0325708 5	k__Bacteria; p__Firmicutes; c__Clostridia; o__Clostridiales; f__Ruminococcaceae; g__; s__
X7a313870bb97fff943b4b91400a6b835	0.0325708 5	k__Bacteria; p__Firmicutes; c__Erysipelotrichi; o__Erysipelotrichales; f__Erysipelotrichaceae; g__p-75-a5; s__
X1674e324c2a5468e18c7dcbaf186fbcd	0.0318508 1	k__Bacteria; p__Proteobacteria; c__Betaproteobacteria; o__Burkholderiales; f__Alcaligenaceae; g__Sutterella; s__
X39993a34fd31592b41224d9e4f81ebb7	0.0318508 1	k__Bacteria; p__Verrucomicrobia; c__Verruco-5; o__WCHB1-41; f__RFP12; g__; s__
c5f3a4a8747b9b59b103e9483331a9e8	0.0318508 1	k__Bacteria; p__Firmicutes; c__Erysipelotrichi; o__Erysipelotrichales; f__Erysipelotrichaceae; g__RFN20; s__

X7100fdfa468d3c9b5bd2fcf5ed08fa08	0.0318508 1	k__Bacteria; p__Bacteroidetes; c__Bacteroidia; o__Bacteroidales; f__[Paraprevotellaceae]; g__[Prevotella]; s__
X19b64c1194c673268602e8d04c38bb36	0.0318508 1	k__Bacteria; p__Bacteroidetes; c__Bacteroidia; o__Bacteroidales; f__[Paraprevotellaceae]; g__YRC22; s__
X64c48d34bfc2889dfe1788d653ecda6d	0.0318508 1	k__Bacteria; p__Bacteroidetes; c__Bacteroidia; o__Bacteroidales; f__[Paraprevotellaceae]; g__YRC22; s__
ed66913c64e880ea0b4cf78c59280664	0.0289567 9	k__Bacteria; p__Proteobacteria; c__Deltaproteobacteria; o__Desulfovibrionales; f__Desulfovibrionaceae; g__Desulfovibrio; s__D168
X26736c7ee02864e6bbab37424d69a4ed	0.028182	k__Bacteria; p__Firmicutes; c__Clostridia; o__Clostridiales; f__Christensenellaceae; g__; s__
X7c104699e795bbf45f90fceac906e6b4	0.0250469 1	k__Bacteria; p__Firmicutes; c__Clostridia; o__Clostridiales; f__; g__; s__
X852ea7a368aaeed1771f6015db845c99	0.0247063 1	k__Bacteria; p__Firmicutes; c__Clostridia; o__Clostridiales; f__Ruminococcaceae; g__; s__
fe813a83837c80d34b56a8f8469d078f	0.0227421	k__Bacteria; p__Firmicutes; c__Clostridia; o__Clostridiales; f__; g__; s__
X5d08d1eb4b8a0672652dd0fc0af2dcf0	0.0227421	k__Bacteria; p__Firmicutes; c__Clostridia; o__Clostridiales; f__Ruminococcaceae; g__Oscillospira; s__
aca1c3a17e598f4af26d49efed7f1ccd	0.0227421	k__Bacteria; p__Firmicutes; c__Clostridia; o__Clostridiales; f__Veillonellaceae; g__Phascolarctobacterium; s__
X9af26e3d0d56c56bd4723ff57308c816	0.0222420 9	k__Bacteria; p__Elusimicrobia; c__Elusimicrobia; o__Elusimicrobiales; f__Elusimicrobiaceae; g__; s__
X13b0362e9465a50a41c280177cb9cb7f	0.0207362 8	k__Bacteria; p__Firmicutes; c__Clostridia; o__Clostridiales; f__Ruminococcaceae; g__; s__
a6b8973aee14771b1609fc644ddc87dc	0.0206885 1	k__Bacteria; p__Firmicutes; c__Clostridia; o__Clostridiales; f__Ruminococcaceae; g__; s__
c018ebf77815e2abed59f10638aa7bc5	0.0180090 3	k__Bacteria; p__Firmicutes; c__Clostridia; o__Clostridiales; f__Lachnospiraceae; g__; s__
a1d0f08cfb19d7aaed2955688f06c25a	0.0180090 3	k__Bacteria; p__Bacteroidetes; c__Bacteroidia; o__Bacteroidales; f__Prevotellaceae; g__Prevotella; s__
cb908ae7d11351c78c0e4ce0d849c744	0.0165032 2	k__Bacteria; p__Firmicutes; c__Clostridia; o__Clostridiales; f__Ruminococcaceae; g__; s__
X86ea0860dfe67c2d8eaa4b80741fae86	0.0165032 2	k__Bacteria; p__Verrucomicrobia; c__Verruco-5; o__WCHB1-41; f__RFP12; g__; s__
X4a566e4b581d463e92973dd4d23cd9d3	0.0159118 3	k__Bacteria; p__Bacteroidetes; c__Bacteroidia; o__Bacteroidales; f__Bacteroidaceae; g__Bacteroides
X13e5e67a2599db5c3cdfa82c4c9ea6fd	0.0154465	k__Bacteria; p__Firmicutes; c__Clostridia; o__Clostridiales; f__Ruminococcaceae; g__; s__
X5bcbc3e28007b9a9d208d6668049410e	0.0136080 5	k__Fungi;p__unidentified;c__unidentified;o__unidentified;f__unidentified;g__unidentified;s__unidentified
X0d8f66a81fffc5aa4408a33c6f4bdc68	0.0116787 8	k__Bacteria; p__Firmicutes; c__Clostridia; o__Clostridiales; f__; g__; s__

X3d6e1834125d9ab26f23a1bf8db2195f	0.0116787 8	k__Bacteria; p__Proteobacteria; c__Deltaproteobacteria; o__GMD14H09; f__; g__; s__
X0b4aeb45916a24fd10e3655fe6481966	0.0116787 8	k__Bacteria; p__Firmicutes; c__Clostridia; o__Clostridiales; f__Christensenellaceae; g__; s__
X4c974b834afe158c4265402ee0bfcbad	0.0116787 8	k__Bacteria; p__Bacteroidetes; c__Bacteroidia; o__Bacteroidales; f__S24-7; g__; s__
X44396f15b10f6577d61a11c6047c939f	0.0116787 8	k__Archaea; p__Euryarchaeota; c__Methanobacteria; o__Methanobacteriales; f__Methanobacteriaceae; g__Methanobrevibacter; s__
acaf226f9fae14dc71a81968d9c33f93	0.0116787 8	k__Bacteria; p__Firmicutes; c__Clostridia; o__Clostridiales; f__Veillonellaceae; g__; s__
f5bfbf665081e0133bdcba9fcfc6023d	0.0116787 8	k__Bacteria; p__Bacteroidetes; c__Bacteroidia; o__Bacteroidales; f__[Paraprevotellaceae]; g__CF231; s__
X16530e5b50b780018210dac05a471715	0.0116787 8	k__Bacteria; p__Firmicutes; c__Clostridia; o__Clostridiales; f__Ruminococcaceae; g__Oscillospira; s__
c66ec67719e54d2cb0afc3154b3c23af	0.0116787 8	k__Bacteria; p__Firmicutes; c__Clostridia; o__Clostridiales; f__; g__; s__
b7d0596261d747c1ac8e1c8b3dca415c	0.0116787 8	k__Bacteria; p__Firmicutes; c__Clostridia; o__Clostridiales; f__; g__; s__
X6fedd2e327d5c758d5df8408295ad0fa	0.0116787 8	k__Bacteria; p__Firmicutes; c__Clostridia; o__Clostridiales; f__Ruminococcaceae; g__; s__
X33518e48b174ac428cb7be1a216c6c6e	0.0042330 5	k__Bacteria; p__Firmicutes; c__Clostridia; o__Clostridiales; f__Lachnospiraceae; g__Coprococcus; s__
X9681441ad736892c2455edc6d137eac1	0.0040171	k__Bacteria; p__Firmicutes; c__Clostridia; o__Clostridiales; f__Ruminococcaceae; g__; s__
X3c62c88287f8b9a3a4e0f1bcd7b31b42	0.0034542 7	k__Bacteria; p__Firmicutes; c__Clostridia; o__Clostridiales; f__Ruminococcaceae; g__; s__
X9253a4863cac514c6004b26d70920768	0.0034542 7	k__Bacteria; p__Firmicutes; c__Clostridia; o__Clostridiales; f__Ruminococcaceae; g__; s__
X514b19a584ab78de6179970686a20e8e	0.0026794 7	k__Bacteria; p__Proteobacteria; c__Gammaproteobacteria; o__Aeromonadales; f__Succinivibrionaceae; g__Ruminobacter; s__
a3ea63b33a623c45f56212bfc2e18b03	0.0026794 7	k__Bacteria; p__Proteobacteria; c__Betaproteobacteria; o__Burkholderiales; f__Alcaligenaceae; g__Sutterella; s__
X11899145b894a8b737a8d3db09a78b4f	0.0023048 1	k__Bacteria; p__Bacteroidetes; c__Bacteroidia; o__Bacteroidales; f__Prevotellaceae; g__Prevotella; s__
da0953e0b53e5f2bc309110e79c16a40	0.0023048 1	k__Bacteria; p__Firmicutes; c__Erysipelotrichi; o__Erysipelotrichales; f__Erysipelotrichaceae; g__RFN20; s__
bac2ee18181e2b913542342c3aa3f3ed	0.0007748	k__Bacteria; p__Firmicutes; c__Erysipelotrichi; o__Erysipelotrichales; f__Erysipelotrichaceae; g__[Eubacterium]; s__biforme
X70e1cbf5a781d1fd56913a209d394405	0.0007748	k__Bacteria; p__Firmicutes; c__Clostridia; o__Clostridiales; f__Ruminococcaceae; g__; s__

X8dcfdd3643e8a1b873a013d7c15804dd	0.0007748	k__Bacteria; p__Firmicutes; c__Clostridia; o__Clostridiales; f__Ruminococcaceae; g__Oscillospira; s__
X022e203c0237562b1af030c2cda7cfd9	6.80E-17	k__Fungi;p__unidentified;c__unidentified;o__unidentified;f__unidentified;g__unidentified;s__unidentified
X1d90719814d690d70dcf3b29d2ec5fc6	3.40E-17	k__Bacteria; p__Firmicutes; c__Erysipelotrichi; o__Erysipelotrichales; f__Erysipelotrichaceae; g__RFN20; s__
b7bb03f41b2044386d4c609b1085f722	3.40E-17	k__Bacteria; p__Verrucomicrobia; c__Verruco-5; o__WCHB1-41; f__RFP12; g__; s__

Supplementary Table2. Sheet5. Key taxa in Chimpanzee identified based on the network hub score.

**ASV**	**HubScore**	**Taxa**
X1682bc65b78e9a7ba3134434c5ad4763	1	k__Fungi;p__unidentified;c__unidentified;o__unidentified;f__unidentified;g__unidentified;s__unidentified
X2c76a1fc7302742580bcaf04942aa3dc	0.940993753	k__Fungi;p__unidentified;c__unidentified;o__unidentified;f__unidentified;g__unidentified;s__unidentified
X905b74ba5bd550960038ca6583f52329	0.871924211	k__Fungi;p__unidentified;c__unidentified;o__unidentified;f__unidentified;g__unidentified;s__unidentified
X8b05add14a66c7c9aac0bd8775bfcb41	0.674199171	k__Fungi;p__Ascomycota;c__Sordariomycetes;o__Xylariales;f__Sporocadaceae
X3a439a0e92578bb8f02ffdeeafdef664	0.629508269	k__Fungi;p__Ascomycota;c__Sordariomycetes;o__Xylariales
X543eb306ab15d1b00b591205bb5b62a5	0.582365282	k__Fungi;p__Ascomycota;c__Sordariomycetes;o__Xylariales;f__Sporocadaceae;g__Neopestalotiopsis;s__Neopestalotiopsis_foedans
X16507e25d999abc59217324eaa106635	0.439625665	k__Fungi;p__Ascomycota;c__Sordariomycetes;o__Hypocreales;f__Nectriaceae;g__Fusarium;s__unidentified
X759c307b9fae00bc323e940bc7f73b6c	0.42746915	k__Fungi;p__Ascomycota;c__Saccharomycetes;o__Saccharomycetales;f__Phaffomycetaceae;g__Wickerhamomyces;s__Wickerhamomyces_anomalus
b9696e4173211cbb79bfa4c491885670	0.422230656	k__Fungi;p__Ascomycota;c__Sordariomycetes;o__Xylariales;f__Sporocadaceae;g__Pestalotiopsis;s__unidentified
X94d5072a6162fc603496ac5985ab8f35	0.332559484	k__Bacteria; p__Firmicutes; c__Erysipelotrichi; o__Erysipelotrichales; f__Erysipelotrichaceae
X54eabf0be27c3a36c7ac00ff3babb65b	0.330543989	k__Bacteria; p__Firmicutes; c__Erysipelotrichi; o__Erysipelotrichales; f__Erysipelotrichaceae; g__Asteroleplasma; s__anaerobium
X7b92c4712a931d594e7980d8c9c878af	0.314770715	k__Fungi;p__Ascomycota;c__Dothideomycetes;o__Dothideales;f__Aureobasidiaceae;g__Aureobasidium;s__Aureobasidium_thailandense
dc381f5656999697f8fb2a10b2931ccb	0.283118382	k__Bacteria; p__Tenericutes; c__Mollicutes; o__Acholeplasmatales
X30c03cd3eec63c8d9a6d637f93ca7b9b	0.272428084	k__Bacteria; p__Firmicutes; c__Erysipelotrichi; o__Erysipelotrichales; f__Erysipelotrichaceae; g__[Eubacterium]; s__cylindroides
d1551ff4d0702a0f8e0b9ac35a25bf6c	0.263185327	k__Fungi;p__Ascomycota;c__Dothideomycetes;o__Pleosporales;f__Periconiaceae;g__Periconia;s__Periconia_byssoides
X1a16ebd30229749f56508bfa99c58cae	0.23727315	k__Bacteria; p__Firmicutes; c__Erysipelotrichi; o__Erysipelotrichales; f__Erysipelotrichaceae; g__Asteroleplasma; s__anaerobium
X6916d386b67ae389d75219a14bf80740	0.234914538	k__Fungi;p__Ascomycota;c__Dothideomycetes;o__Capnodiales;f__Cladosporiaceae;g__Cladosporium
X2bb38254f5f0297a2d6540517a7b009c	0.227940542	k__Bacteria; p__Proteobacteria; c__Alphaproteobacteria; o__RF32; f__; g__; s__

X1072825caf7aef9acfeecafe306fa16b	0.220715897	k__Fungi;p__Ascomycota;c__Dothideomycetes;o__Pleosporales;f__Didymellaceae;g__Boeremia;s__Boeremia_exigua
X5579452b3e48a6507174b8add11f30bb	0.218534739	k__Fungi;p__Ascomycota;c__Sordariomycetes;o__Trichosphaeriales;f__Trichosphaeriaceae;g__Nigrospora;s__Nigrospora_oryzae
b8b4e7f53bee3ab157156e6aa3296055	0.216384224	k__Bacteria; p__Firmicutes; c__Clostridia; o__Clostridiales; f__Lachnospiraceae; g__; s__
X9d05afa2b6632b66b4a978a211e498ef	0.214395554	k__Bacteria; p__Actinobacteria; c__Coriobacteriia; o__Coriobacteriales; f__Coriobacteriaceae; g__; s__
f0a78a8aaf56aa57897de30884e8e374	0.21324334	k__Bacteria; p__Verrucomicrobia; c__Verruco-5; o__WCHB1-41; f__RFP12; g__; s__
X4969b714968843044292289c42999f85	0.209676833	k__Fungi;p__Ascomycota;c__Dothideomycetes
b0aeb5d3ee0bddfec14bece683a41a79	0.197870817	k__Bacteria; p__Firmicutes; c__Clostridia; o__Clostridiales; f__; g__; s__
X26b0fd3cb46859713999bbf58faea436	0.197870817	k__Archaea; p__Euryarchaeota; c__Thermoplasmata; o__E2; f__[Methanomassiliicoccaceae]; g__vadinCA11; s__
X2e4f2b53b856c4def6d021d01f5abb70	0.197531099	k__Bacteria; p__Firmicutes; c__Clostridia; o__Clostridiales; f__Lachnospiraceae; g__[Ruminococcus]; s__gnavus
X0f5f4516676336a6d4036cfb9d6bd269	0.197531099	k__Bacteria; p__Actinobacteria; c__Coriobacteriia; o__Coriobacteriales; f__Coriobacteriaceae; g__; s__
c2bf90ec366834cc59ac912f540e5fd2	0.196413764	k__Bacteria; p__Firmicutes; c__Clostridia; o__Clostridiales; f__Lachnospiraceae; g__Oribacterium; s__
X77673dde68d3a9e7de95dce383eb4c7a	0.184516512	k__Bacteria; p__Firmicutes; c__Clostridia; o__Clostridiales; f__[Mogibacteriaceae]; g__; s__
d5c34a5c01d28b731f12b218bbd43d75	0.150810329	k__Bacteria; p__Firmicutes; c__Clostridia; o__Clostridiales; f__Ruminococcaceae; g__; s__
X8a7be2a5ee5dbc9748aa8d62232ef517	0.149616162	k__Bacteria; p__Spirochaetes; c__Spirochaetes; o__Sphaerochaetales; f__Sphaerochaetaceae; g__Sphaerochaeta; s__
c0db21fc90b9cd7ed694875d1f852ff3	0.143550834	k__Bacteria; p__Cyanobacteria; c__4C0d-2; o__YS2; f__; g__; s__
X121ce4cb34a821cbfed89ea7fe14a91e	0.138788226	k__Fungi;p__Ascomycota;c__Dothideomycetes;o__Capnodiales;f__Cladosporiaceae;g__Cladosporium
X202e47cf9862390db2821ac92ad562e0	0.131677915	k__Bacteria; p__Bacteroidetes; c__Bacteroidia; o__Bacteroidales; f__[Paraprevotellaceae]; g__YRC22; s__
a2d300d1e9c621dbc5239cc092d433c3	0.12859903	k__Bacteria; p__Firmicutes; c__Clostridia; o__Clostridiales; f__; g__; s__
X85a8502c1faf8a0e682c1c689fbda1c8	0.126806105	k__Fungi;p__Ascomycota;c__Dothideomycetes;o__Pleosporales;f__Didymosphaeriaceae;g__Alloconiothyrium;s__Alloconiothyrium_aptrootii
X72fd2576530aaa70b72a2006cf53014c	0.121982874	k__Bacteria; p__Tenericutes; c__Mollicutes; o__Anaeroplasmatales; f__Anaeroplasmataceae; g__; s__

bb7bb46bb34c7f3d794d9478106efa85	0.116921446	k__Bacteria; p__Verrucomicrobia; c__Verruco-5; o__WCHB1-41; f__RFP12; g__; s__
fd53879206e220088465f824f06579e8	0.110177542	k__Bacteria; p__Firmicutes; c__Clostridia; o__Clostridiales; f__; g__; s__
ff2c9c54fc9c496e68a13025bb4acfa2	0.107631682	k__Bacteria; p__Tenericutes; c__Mollicutes; o__RF39; f__; g__; s__
X1f273a9cf201f75859455ab67d5e123b	0.105334275	k__Bacteria
df544d84ee7ef1afbc66210d1706fff8	0.103371142	k__Bacteria; p__Actinobacteria; c__Coriobacteriia; o__Coriobacteriales; f__Coriobacteriaceae; g__; s__
a171d6b000917ac0e0a39cb0c8b2fc1b	0.103200494	k__Fungi;p__Ascomycota;c__Dothideomycetes;o__Botryosphaeriales;f__Botryosphaeriaceae;g__Lasiodiplodia
X4a0b292ba716582f9af46694458c0b9b	0.101268473	k__Bacteria; p__Cyanobacteria; c__Chloroplast; o__Streptophyta; f__; g__; s__
X34bf4e2da6fa3ee1569de7b788f44d8e	0.099268947	k__Bacteria; p__Bacteroidetes; c__Bacteroidia; o__Bacteroidales; f__Prevotellaceae; g__Prevotella; s__
X92186ee2c33d31b5787c594e18fb8fa9	0.088391285	k__Bacteria; p__Bacteroidetes; c__Bacteroidia; o__Bacteroidales; f__Prevotellaceae; g__Prevotella; s__copri
X7242dd3e8dfdf19374b99109f0eccd19	0.088391285	k__Bacteria; p__TM7; c__TM7-3; o__CW040; f__F16; g__; s__
X62b5223d4d79e28288ee4daa241352f1	0.087282186	k__Bacteria; p__Firmicutes; c__Clostridia; o__Clostridiales; f__Veillonellaceae; g__Dialister; s__
cd03e9d7569ffc8418ba9676033cbaf7	0.087282186	k__Bacteria; p__Firmicutes; c__Clostridia; o__Clostridiales; f__; g__; s__
aae4c4ed528ae16ec417df816699cfdb	0.086868352	k__Bacteria; p__Firmicutes; c__Clostridia; o__Clostridiales; f__Ruminococcaceae; g__; s__
X9612ad0ea299179204ad654e41183f49	0.086224296	k__Fungi;p__Ascomycota;c__Dothideomycetes;o__Capnodiales;f__Mycosphaerellaceae;g__Passalora;s__Passalora_perplexa
d21710911b184d6eee705efbd62291d6	0.081751052	k__Fungi;p__Ascomycota;c__Dothideomycetes;o__Pleosporales;f__Didymosphaeriaceae
X19d7214df21f837591c11e7a9e12f1c6	0.07952831	k__Bacteria; p__Bacteroidetes; c__Bacteroidia; o__Bacteroidales; f__Prevotellaceae; g__Prevotella; s__
f6be8fd3434273e20c0b5a1461ee3c85	0.070343614	k__Bacteria; p__Firmicutes; c__Clostridia; o__Clostridiales; f__; g__; s__
d80066763c0ef01e9782b7db1272e31f	0.0702543	k__Bacteria; p__Bacteroidetes; c__Bacteroidia; o__Bacteroidales; f__Prevotellaceae; g__; s__
e865f29a716e8d51f83931befa951240	0.069567716	k__Bacteria; p__Firmicutes; c__Erysipelotrichi; o__Erysipelotrichales; f__Erysipelotrichaceae; g__; s__
b494a8d807d2fb57fd2d9d828ef8552d	0.068545959	k__Bacteria; p__Tenericutes; c__Mollicutes; o__RF39; f__; g__; s__

X37f004671c936285c26471120596b2f5	0.068545959	k__Bacteria; p__Firmicutes; c__Clostridia; o__Clostridiales; f__Clostridiaceae; g__Sarcina; s__
X0563d6ca03c8b32f7003744172f738d3	0.066192902	k__Bacteria; p__Bacteroidetes; c__Bacteroidia; o__Bacteroidales; f__; g__; s__
X74b7dbcd58dc83617d1a82d321245a1e	0.065939014	k__Bacteria; p__Spirochaetes; c__Spirochaetes; o__Spirochaetales; f__Spirochaetaceae; g__Treponema; s__
X79a2ad292bd3e1f5e872294165fc7fee	0.061334301	k__Bacteria; p__Verrucomicrobia; c__Verruco-5; o__WCHB1-41; f__RFP12; g__; s__
X4bc284bffb22616e035ba6ba03948917	0.061334301	k__Bacteria; p__Firmicutes; c__Clostridia; o__Clostridiales; f__Lachnospiraceae; g__; s__
X6a45e7962527c5d81859f75647fd376f	0.061334301	k__Bacteria; p__Firmicutes; c__Clostridia; o__Clostridiales; f__; g__; s__
ba00d2e0c74c53a4f32a5b6c48859a9c	0.060994583	k__Bacteria; p__Bacteroidetes; c__Bacteroidia; o__Bacteroidales; f__Prevotellaceae; g__Prevotella; s__
X18e0a23e81430f15c9040071fef9b478	0.060994583	k__Bacteria; p__Firmicutes; c__Erysipelotrichi; o__Erysipelotrichales; f__Erysipelotrichaceae; g__Asteroleplasma; s__anaerobium
X56a8014ee62437086dba0e4bc05e192c	0.060994583	k__Bacteria; p__Firmicutes; c__Clostridia; o__Clostridiales; f__; g__; s__
eafa0985b50a707247b37e92ab00f16c	0.060994583	k__Bacteria; p__Bacteroidetes; c__Bacteroidia; o__Bacteroidales; f__; g__; s__
X646c60019eae06733055ec4c46643011	0.060994583	k__Bacteria; p__Firmicutes; c__Clostridia; o__Clostridiales; f__Ruminococcaceae; g__; s__
X2c737630a237bcdb4fa848e5fe047e0b	0.05289211	k__Fungi;p__Ascomycota;c__Dothideomycetes;o__Pleosporales;f__Didymosphaeriaceae
X033f44f6ab9e723489b1802782125bc0	0.046235345	k__Bacteria; p__Actinobacteria; c__Coriobacteriia; o__Coriobacteriales; f__Coriobacteriaceae; g__; s__
X6fdb85b72abc94a4a93a9f2bd9294718	0.040965679	k__Bacteria; p__Firmicutes; c__Clostridia; o__Clostridiales; f__Lachnospiraceae; g__; s__
X50f48be0c99ba0de46f584073eca2397	0.040965679	k__Bacteria; p__Firmicutes; c__Clostridia; o__Clostridiales; f__Ruminococcaceae; g__; s__
X0a7fe3595e40caeafecb26f55ecf526d	0.040110046	k__Fungi;p__Ascomycota;c__Dothideomycetes;o__Capnodiales;f__Cladosporiaceae;g__Cladosporium
X6517fc81700d85e49ca3f67af1169049	0.03983259	k__Bacteria; p__Lentisphaerae; c__[Lentisphaeria]; o__Z20; f__R4-45B; g__; s__
X0890ba8b8ffaf51f54ceaeb56a836435	0.035451895	k__Bacteria; p__Firmicutes; c__Clostridia; o__Clostridiales; f__Veillonellaceae; g__Dialister; s__
X95636cde183c77c2a0109d48f419d158	0.034039361	k__Bacteria; p__Firmicutes; c__Clostridia; o__Clostridiales; f__; g__; s__
X4f120ddf78e0d768e7b70f4d08985ea2	0.033842572	k__Bacteria; p__Verrucomicrobia; c__Verruco-5; o__WCHB1-41; f__RFP12; g__; s__

f27951ed312a5764b0edb9f6d4eb2e5b	0.033746344	k__Bacteria; p__Bacteroidetes; c__Bacteroidia; o__Bacteroidales; f__RF16; g__; s__
X0777120ba507788e1ede4ee5b1b280cd	0.030924858	k__Bacteria; p__Firmicutes; c__Clostridia; o__Clostridiales; f__; g__; s__
X4934539d520aaa8493aa9e1fc6ee22e5	0.030924858	k__Bacteria; p__Actinobacteria; c__Coriobacteriia; o__Coriobacteriales; f__Coriobacteriaceae; g__; s__
X9de8773c1e0312e45765aef7d2f0e14b	0.030924858	k__Bacteria; p__Bacteroidetes; c__Bacteroidia; o__Bacteroidales
aa9a76f47ffd50253f375650a03efd1c	0.03027538	k__Bacteria; p__Firmicutes; c__Clostridia; o__Clostridiales
d06eb6064ab56f92985e11b40b0ecb54	0.030069725	k__Bacteria; p__Bacteroidetes; c__Bacteroidia; o__Bacteroidales; f__; g__; s__
X274d44a105684512d6df0a983a5b6663	0.030069725	k__Bacteria; p__Firmicutes; c__Clostridia; o__Clostridiales; f__Clostridiaceae; g__02d06; s__
X43fe08d52469682ebdddda3177c2d0f9	0.030069725	k__Bacteria; p__Bacteroidetes; c__Bacteroidia; o__Bacteroidales; f__; g__; s__
X168b33d848098ec8aa791e9ccda5b926	0.030069725	k__Archaea; p__Euryarchaeota; c__Thermoplasmata; o__E2; f__[Methanomassiliicoccaceae]; g__vadinCA11; s__
ca164e68ce5db67ae0182d9c3bafc5fa	0.02970123	k__Bacteria; p__Firmicutes; c__Clostridia; o__Clostridiales; f__Christensenellaceae; g__; s__
X2d1905db9c96d6429c3f04e1ed4f718f	0.028276273	k__Bacteria; p__Bacteroidetes; c__Bacteroidia; o__Bacteroidales; f__[Paraprevotellaceae]; g__YRC22; s__
X52cdc265412299f4b1520f751a1f185a	0.028276273	k__Bacteria; p__Firmicutes; c__Clostridia; o__Clostridiales; f__Ruminococcaceae; g__Ruminococcus; s__flavefaciens
X2dce8db2501d65ce638c43b33f8ca0b6	0.028207438	k__Bacteria; p__Bacteroidetes; c__Bacteroidia; o__Bacteroidales; f__Prevotellaceae; g__Prevotella; s__
ca6a6f92951f0313a72f4d332b4a5006	0.027433407	k__Bacteria; p__Bacteroidetes; c__Bacteroidia; o__Bacteroidales; f__RF16; g__; s__
X740df5c41344bf38464fc7ebfbdf8a77	0.026287603	k__Bacteria; p__Bacteroidetes; c__Bacteroidia; o__Bacteroidales; f__[Paraprevotellaceae]; g__; s__
X46b6b6856a2e1567e55108a860b62f08	0.026287603	k__Bacteria; p__Firmicutes; c__Clostridia; o__Clostridiales; f__Lachnospiraceae; g__Oribacterium; s__
a891fb44241e433fa8acf251ef4d328f	0.026287603	k__Bacteria; p__Firmicutes; c__Clostridia; o__Clostridiales; f__Lachnospiraceae; g__; s__
X311af65847e08e70a5d00e59a36bb8ef	0.025444737	k__Bacteria; p__Proteobacteria; c__Epsilonproteobacteria; o__Campylobacterales; f__Campylobacteraceae; g__Campylobacter; s__
X6d757dde747b98331cd126b525172bd7	0.025444737	k__Bacteria; p__Firmicutes; c__Erysipelotrichi; o__Erysipelotrichales; f__Erysipelotrichaceae; g__RFN20; s__
X96c7a46d58ffffc47ecc1cf2fb033f11	0.025444737	k__Bacteria; p__Firmicutes; c__Clostridia; o__Clostridiales; f__Ruminococcaceae; g__; s__

X6cabec667e80dda2f9a2b640df76b9d0	0.024264072	k__Bacteria; p__Firmicutes; c__Erysipelotrichi; o__Erysipelotrichales; f__Erysipelotrichaceae; g__RFN20; s__
X00a96fbd0ac34bfd245f9c24f8737f7d	0.024264072	k__Bacteria; p__Firmicutes; c__Clostridia; o__Clostridiales; f__Lachnospiraceae; g__Blautia; s__obeum
b1ee18482360ed4b9c6bbd4de6b1f8d1	0.024264072	k__Bacteria; p__Firmicutes; c__Clostridia; o__Clostridiales; f__Lachnospiraceae; g__; s__
caee7bc24a3a5ac8c2919c247de232c9	0.024264072	k__Bacteria; p__Firmicutes; c__Erysipelotrichi; o__Erysipelotrichales; f__Erysipelotrichaceae; g__Bulleidia; s__p-1630-c5
X74cab2281116a65629f10e53adbadf70	0.023784233	k__Bacteria; p__Bacteroidetes; c__Bacteroidia; o__Bacteroidales; f__[Paraprevotellaceae]; g__; s__
X927b104e3bc0dc538716b193b41a211b	0.023669426	k__Bacteria; p__Verrucomicrobia; c__Verruco-5; o__WCHB1-41; f__RFP12; g__; s__
X38fc79d76b28e16985c969515dacb6d8	0.022869534	k__Bacteria; p__Firmicutes; c__Erysipelotrichi; o__Erysipelotrichales; f__Erysipelotrichaceae; g__p-75-a5; s__
X847dd19dd00c43810f3051cf594a1a77	0.02278545	k__Bacteria
f13b7ca216600d3cc3b0e6a56ef0e09b	0.02214211	k__Bacteria; p__Firmicutes; c__Erysipelotrichi; o__Erysipelotrichales; f__Erysipelotrichaceae; g__; s__
X5b65913d3c536b4c350f284f72f72266	0.019246576	k__Bacteria; p__; c__; o__; f__; g__; s__
f269348cc063f545ca6552393a42a6e2	0.018513407	k__Bacteria; p__Verrucomicrobia; c__Opitutae; o__[Cerasicoccales]; f__[Cerasicoccaceae]; g__; s__
X19dea030b1c9741f5a6dad4250b7b5f6	0.018513407	k__Bacteria; p__Spirochaetes; c__Spirochaetes; o__Sphaerochaetales; f__Sphaerochaetaceae; g__Sphaerochaeta; s__
X3903585322b1fabd7ee0672e2dcf8701	0.018513407	k__Bacteria; p__Proteobacteria; c__Betaproteobacteria; o__Burkholderiales; f__Alcaligenaceae; g__Sutterella; s__
X6cec9979c3e76d4316a289bf1792356f	0.018513407	k__Bacteria; p__Proteobacteria; c__Betaproteobacteria; o__Burkholderiales; f__Alcaligenaceae; g__Sutterella; s__
X6d92f62973cac2e800df70551c1ffbd8	0.018513407	k__Bacteria; p__Firmicutes; c__Erysipelotrichi; o__Erysipelotrichales; f__Erysipelotrichaceae; g__[Eubacterium]; s__biforme
X68b655ce0b3de7da0db0b3b6869b440d	0.016524738	k__Bacteria; p__Actinobacteria; c__Coriobacteriia; o__Coriobacteriales; f__Coriobacteriaceae; g__; s__
a18c0c17fce3d52dc60506e8730bc996	0.016524738	k__Bacteria; p__Firmicutes; c__Clostridia; o__Clostridiales; f__Ruminococcaceae
X197575c617ca3ab2b6561e1c14b87507	0.016524738	k__Bacteria; p__Actinobacteria; c__Coriobacteriia; o__Coriobacteriales; f__Coriobacteriaceae; g__Adlercreutzia; s__
X2b302c2965a32285467d1c3930d5ce2b	0.016524738	k__Bacteria; p__Verrucomicrobia; c__Verruco-5; o__WCHB1-41; f__RFP12; g__; s__
X63006813a2e59cd0d5424633377e8db2	0.016179495	k__Bacteria; p__Chloroflexi; c__Anaerolineae; o__Anaerolineales; f__Anaerolinaceae; g__SHD-231; s__

X8e100aace26401d737b2b1b49df77673	0.015525954	k__Bacteria; p__Firmicutes; c__Clostridia; o__Clostridiales; f__Lachnospiraceae; g__; s__
X656fc22d077cc5d6ef83285bcd081cbe	0.015525954	k__Bacteria; p__Proteobacteria; c__Alphaproteobacteria; o__RF32; f__; g__; s__
eff99032d2e35a979c83a1273f3f739d	0.014749426	k__Archaea; p__Euryarchaeota; c__Methanobacteria; o__Methanobacteriales; f__Methanobacteriaceae; g__Methanobrevibacter; s__
fa4231d184b1582773f6d5ca65fe51f4	0.01433194	k__Fungi;p__Ascomycota;c__Dothideomycetes;o__Botryosphaeriales;f__Aplosporellaceae;g__Aplosporella
X4db84fa02ed703c170eda12a634e2abe	0.013324824	k__Bacteria; p__Firmicutes; c__Clostridia; o__Clostridiales; f__Ruminococcaceae; g__; s__
b0f1939a709af729120ace2bf4a973c9	0.011741485	k__Bacteria; p__Bacteroidetes; c__Bacteroidia; o__Bacteroidales; f__Prevotellaceae; g__Prevotella; s__
X90ba8ba071ad1adb1b1ea3f783d64b12	0.010490289	k__Bacteria; p__Bacteroidetes; c__Bacteroidia; o__Bacteroidales; f__Prevotellaceae; g__Prevotella; s__
X3e4fa97314839bb499633b9156f9ee5a	0.010341008	k__Fungi;p__Ascomycota;c__Dothideomycetes;o__Capnodiales;f__Cladosporiaceae;g__Cladosporium
d6cd19c0894d78aaada198c572506a71	0.010080981	k__Bacteria; p__Firmicutes; c__Erysipelotrichi; o__Erysipelotrichales; f__Erysipelotrichaceae; g__RFN20; s__
X017570ebc40035b4e9c2e5d3e3dffc05	0.009762865	k__Bacteria; p__Spirochaetes; c__Spirochaetes; o__Sphaerochaetales; f__Sphaerochaetaceae; g__Sphaerochaeta; s__
fd408d248a5bc3d4bf358d58f8e0ce2c	0.00892	k__Archaea; p__Euryarchaeota; c__Thermoplasmata; o__E2; f__[Methanomassiliicoccaceae]; g__vadinCA11; s__
fd325e685fae520777942e25af2d6ee0	0.00892	k__Bacteria; p__Proteobacteria; c__Deltaproteobacteria; o__Desulfovibrionales; f__Desulfovibrionaceae; g__Desulfovibrio; s__D168
e63f7e072b2de3bbaf23e446796d7acd	0.00892	k__Bacteria; p__Spirochaetes; c__Spirochaetes; o__Spirochaetales; f__Spirochaetaceae; g__Treponema; s__
X6997eb891780dac0114c005b4ceccd72	0.00892	k__Bacteria; p__Bacteroidetes; c__Bacteroidia; o__Bacteroidales; f__[Paraprevotellaceae]; g__[Prevotella]; s__
ea626bd8ae7b37fe0cade0ae60e9cbda	0.00892	k__Bacteria; p__Bacteroidetes; c__Bacteroidia; o__Bacteroidales; f__[Paraprevotellaceae]; g__YRC22; s__
X185eaa5c8c2f93c50339d283ab1ecfeb	0.00892	k__Bacteria; p__Bacteroidetes; c__Bacteroidia; o__Bacteroidales; f__; g__; s__
X8feadd6e25ff93f26d31da82c2f30819	0.00892	k__Bacteria; p__Bacteroidetes; c__Bacteroidia; o__Bacteroidales
X0a68ec564e278b0246c920ad7de41880	0.00892	k__Bacteria; p__Firmicutes; c__Clostridia; o__Clostridiales; f__Lachnospiraceae; g__Blautia; s__
a46b89f8bd35cec94fa7986c221e6a8f	0.00892	k__Bacteria; p__Bacteroidetes; c__Bacteroidia; o__Bacteroidales; f__; g__; s__
b4e1107ef19fa11e16b0aa4873de331a	0.00892	k__Bacteria; p__Firmicutes; c__Clostridia; o__Clostridiales; f__; g__; s__
eb19ee5d5f64be63a0baf81435d48434	0.00892	k__Bacteria; p__Firmicutes; c__Clostridia; o__Clostridiales; f__Ruminococcaceae; g__; s__
X94971b5330b72e64e7fe0fafda7a8c1f	0.00892	k__Bacteria; p__Bacteroidetes; c__Bacteroidia; o__Bacteroidales; f__Prevotellaceae; g__Prevotella; s__
b726627e18f29e0da5289f81ca294f92	0.00892	k__Bacteria; p__Firmicutes; c__Clostridia; o__Clostridiales; f__Ruminococcaceae

X80f23fe302e867ca241bbbb9773880fe	0.007259496	k__Bacteria; p__Firmicutes; c__Clostridia; o__Clostridiales; f__Lachnospiraceae; g__; s__
dda67a36816073f18ab90d79033aa821	0.007259496	k__Bacteria; p__Firmicutes; c__Clostridia; o__Clostridiales; f__Ruminococcaceae; g__; s__
X1062c3cd627877791824f2c58794eed2	0.007259496	k__Bacteria; p__Firmicutes; c__Erysipelotrichi; o__Erysipelotrichales; f__Erysipelotrichaceae; g__RFN20; s__
X923eb6134727a20b2fd6d2da910fa20d	0.006065329	k__Bacteria; p__Tenericutes; c__Mollicutes; o__Anaeroplasmatales; f__Anaeroplasmataceae; g__; s__
X6e15035874a659bc18efcc98b983b4f4	0.005750664	k__Bacteria; p__Verrucomicrobia; c__Verruco-5; o__WCHB1-41; f__RFP12; g__; s__
X2393b358b70b807e64b122d71982aaf3	0.005750664	k__Bacteria; p__Actinobacteria; c__Coriobacteriia; o__Coriobacteriales; f__Coriobacteriaceae; g__Collinsella; s__
X242e2043679cb2e44a5c3f3189184b42	0.005750664	k__Bacteria; p__Firmicutes; c__Erysipelotrichi; o__Erysipelotrichales; f__Erysipelotrichaceae; g__Bulleidia; s__
c0241dff3c50c2bd8484d30accbbea2a	0.003720622	k__Bacteria; p__Cyanobacteria; c__4C0d-2; o__YS2; f__; g__; s__
X0e59a95a37ad2758e345f0bbd32d55a4	0.003720622	k__Bacteria; p__Actinobacteria; c__Coriobacteriia; o__Coriobacteriales; f__Coriobacteriaceae; g__; s__
X67470a0c54968e67640a99a3278b2df0	0.003720622	k__Bacteria; p__Actinobacteria; c__Actinobacteria; o__Bifidobacteriales; f__Bifidobacteriaceae; g__; s__
d6f3aa6bc341f88139ad91564cceb0c3	0.003720622	k__Bacteria; p__Actinobacteria; c__Coriobacteriia; o__Coriobacteriales; f__Coriobacteriaceae; g__; s__
X43f56e23a3e9dfa0ee5916032e44b6f4	0.003720622	k__Bacteria; p__Firmicutes; c__Clostridia; o__Clostridiales; f__Ruminococcaceae; g__; s__
X21e391c46c36281c329617ce01b55fe0	0.003720622	k__Bacteria; p__Verrucomicrobia; c__Verruco-5; o__WCHB1-41; f__RFP12; g__; s__
X6f43ce86c60a2147e9f5fd2f633aa885	0.002821486	k__Bacteria; p__Bacteroidetes; c__Bacteroidia; o__Bacteroidales; f__Porphyromonadaceae; g__Parabacteroides; s__
X9192b3275c36e56540678451adfa601f	0.002821486	k__Bacteria; p__Firmicutes; c__Clostridia; o__Clostridiales; f__Ruminococcaceae
X4d967e590e9767040a1bd394a3de1ef7	0.001028785	k__Bacteria; p__Firmicutes; c__Clostridia; o__Clostridiales; f__Lachnospiraceae
X54947f672cc093728ece078418837f82	0.00100816	k__Bacteria; p__Spirochaetes; c__Spirochaetes; o__Sphaerochaetales; f__Sphaerochaetaceae; g__Sphaerochaeta; s__
c99d06b3b7c5b133e4406b29dce4d7cd	0.00100816	k__Bacteria; p__Actinobacteria; c__Coriobacteriia; o__Coriobacteriales; f__Coriobacteriaceae; g__Adlercreutzia; s__
X94a302cc7a648f23c27353f085b24e06	0.00100816	k__Bacteria; p__Firmicutes; c__Clostridia; o__Clostridiales; f__Veillonellaceae; g__Anaerovibrio; s__
X0332ce7f5e6cfa9c1db043e2ef4f70e5	0.00074294	k__Bacteria; p__Firmicutes; c__Clostridia; o__Clostridiales; f__Ruminococcaceae; g__Ruminococcus; s__

b1b0fe59b639bd6aa16bb11b28bfd06e	0.000727424	k__Archaea; p__Euryarchaeota; c__Methanobacteria; o__Methanobacteriales; f__Methanobacteriaceae; g__Methanosphaera; s__
X42457fbfa9a7109e71cf5dab55aafa0e	0.000727424	k__Bacteria; p__Firmicutes; c__Erysipelotrichi; o__Erysipelotrichales; f__Erysipelotrichaceae; g__RFN20; s__
a104cdb42c20a3d79353827c47a81212	0.000727424	k__Bacteria; p__Bacteroidetes; c__Bacteroidia; o__Bacteroidales; f__Prevotellaceae; g__Prevotella; s__
X5d6ba04b036af82be19961e4d19d15dd	0.000293197	k__Fungi;p__Ascomycota;c__Sordariomycetes;o__Trichosphaeriales;f__Trichosphaeriaceae;g__Nigrospora;s__Nigrospora_oryzae
X288359d621a383c1e46f0c0418660242	0.000220577	k__Fungi;p__Ascomycota;c__Dothideomycetes;o__Botryosphaeriales;f__Botryosphaeriaceae;g__Lasiodiplodia
bccc0003db4346620e231e2e5ebe7af3	1.55E-05	k__Bacteria; p__Firmicutes; c__Clostridia; o__Clostridiales
X90762fb257f23f6d1320d750f166d48b	1.55E-05	k__Bacteria; p__Firmicutes; c__Clostridia; o__Clostridiales; f__Ruminococcaceae; g__; s__

Supplementary Table2. Sheet6. Key taxa in Mangabey identified based on the network hub score.

ASV	HubScore	Taxa
X349193569e3248c780770d1861ce93bb	1	k__Bacteria; p__Bacteroidetes; c__Bacteroidia; o__Bacteroidales; f__Prevotellaceae; g__Prevotella; s__
X5ab7bae3d45cdd5979aeda9bbe636210	0.967668044	k__Bacteria; p__Firmicutes; c__Clostridia; o__Clostridiales; f__Ruminococcaceae
X6420671c26eef32065e0275c8cd6701e	0.967668044	k__Bacteria; p__Firmicutes; c__Clostridia; o__Clostridiales; f__Peptostreptococcaceae
da6a7cba87e0895b9cbe6037b9bd8b3b	0.967668044	k__Bacteria; p__Bacteroidetes; c__Bacteroidia; o__Bacteroidales; f__Prevotellaceae; g__Prevotella; s__copri
X7fd072aff74564746d11ca0c54ee6ae2	0.943720426	k__Bacteria; p__Tenericutes; c__RF3; o__ML615J-28; f__; g__; s__
e865f29a716e8d51f83931befa951240	0.943720426	k__Bacteria; p__Firmicutes; c__Erysipelotrichi; o__Erysipelotrichales; f__Erysipelotrichaceae; g__; s__
d54f71f28a2092111fd16b901d39254c	0.872182181	k__Bacteria; p__Firmicutes; c__Clostridia; o__Clostridiales; f__[Mogibacteriaceae]; g__; s__
X3b3f9c7bb2ca421e0c31645737223b39	0.872182181	k__Bacteria; p__Verrucomicrobia; c__Verruco-5; o__WCHB1-41; f__RFP12; g__; s__
X26c910e914551d2c61538c1d0418e56d	0.872182181	k__Bacteria; p__Firmicutes; c__Clostridia; o__Clostridiales; f__Ruminococcaceae; g__; s__
e2f5328726e4b0bb7e365a0c1d9e85b7	0.872182181	k__Bacteria; p__Firmicutes; c__Clostridia; o__Clostridiales; f__Ruminococcaceae; g__; s__
X13e5e67a2599db5c3cdfa82c4c9ea6fd	0.872182181	k__Bacteria; p__Firmicutes; c__Clostridia; o__Clostridiales; f__Ruminococcaceae; g__; s__
X7327a55af477aa89dc73611f2806c3e9	0.872182181	k__Bacteria; p__Firmicutes; c__Clostridia; o__Clostridiales; f__Ruminococcaceae; g__; s__
daaca63ff109dd6819af95c487d9314e	0.872182181	k__Bacteria; p__Firmicutes; c__Bacilli; o__Lactobacillales; f__Lactobacillaceae; g__Lactobacillus; s__ruminis
X33518e48b174ac428cb7be1a216c6c6e	0.689880742	k__Bacteria; p__Firmicutes; c__Clostridia; o__Clostridiales; f__Lachnospiraceae; g__Coprococcus; s__
X2ee8f2b3ab2530d891e82c4e2d48f761	0.656603123	k__Bacteria; p__Firmicutes; c__Clostridia; o__Clostridiales; f__; g__; s__
X6fb70d8c2aa551fc31eb19afebf7d74e	0.656603123	k__Bacteria; p__Bacteroidetes; c__Bacteroidia; o__Bacteroidales; f__Prevotellaceae; g__Prevotella; s__
X6d4e639b81485e3714ccdc9ffa6a147b	0.618342496	k__Bacteria; p__Firmicutes; c__Erysipelotrichi; o__Erysipelotrichales; f__Erysipelotrichaceae; g__; s__
X30af5a573043a5f612bbfee09a38ef0f	0.618342496	k__Bacteria; p__Firmicutes; c__Clostridia; o__Clostridiales; f__[Mogibacteriaceae]; g__; s__
X35cbb315e019e0a8768d4b73862df415	0.618342496	k__Bacteria; p__Actinobacteria; c__Coriobacteriia; o__Coriobacteriales; f__Coriobacteriaceae; g__Collinsella; s__aerofaciens
X54a2bfb0a96adc52c4ccca239ad01466	0.590506494	k__Bacteria; p__Firmicutes; c__Clostridia; o__Clostridiales; f__Ruminococcaceae; g__Ruminococcus; s__
X65990c74559e4adfaa672a884ad7d464	0.590506494	k__Bacteria; p__Firmicutes; c__Clostridia; o__Clostridiales; f__Lachnospiraceae; g__Coprococcus; s__catus
X025227aa4f7242f78a33a3452451eff4	0.590506494	k__Bacteria; p__Actinobacteria; c__Coriobacteriia; o__Coriobacteriales; f__Coriobacteriaceae; g__; s__
X5a6c87d6a4eb5e114959f6192f29b641	0.408205055	k__Bacteria; p__Actinobacteria; c__Actinobacteria; o__Bifidobacteriales; f__Bifidobacteriaceae; g__Bifidobacterium; s__adolescentis
X97f07ddfd7af6b5e43d0c5516aced90e	0.37716155	k__Bacteria; p__Actinobacteria; c__Coriobacteriia; o__Coriobacteriales; f__Coriobacteriaceae; g__; s__

ebb8f381bdd9f66ee55229f60acb97bb	0.349325548	k__Bacteria; p__Firmicutes; c__Clostridia; o__Clostridiales; f__Clostridiaceae
X274d44a105684512d6df0a983a5b6663	0.336666809	k__Bacteria; p__Firmicutes; c__Clostridia; o__Clostridiales; f__Clostridiaceae; g__02d06; s__
X5654714fd17021f92582b02756943a86	0.336666809	k__Bacteria; p__Firmicutes; c__Clostridia; o__Clostridiales; f__; g__; s__
X3aa6c8918fd1f91535b598ff78916c4e	0.336666809	k__Bacteria; p__Actinobacteria; c__Coriobacteriia; o__Coriobacteriales; f__Coriobacteriaceae; g__; s__
X9797582f6b7d2be641e0f060ee1d3a20	0.336666809	k__Bacteria; p__Firmicutes; c__Clostridia; o__Clostridiales; f__Veillonellaceae; g__Dialister; s__
X9d42be9468b9b3db4aeba16fed70165b	0.260312855	k__Bacteria; p__Bacteroidetes; c__Bacteroidia; o__Bacteroidales; f__Prevotellaceae; g__; s__
X61535a40adb8221483821e3984d0b1ac	0.260006659	k__Bacteria; p__Firmicutes; c__Clostridia; o__Clostridiales; f__Lachnospiraceae
X9b7e485947ffcf56e03235dc774335c4	0.138373127	k__Bacteria; p__Firmicutes; c__Clostridia; o__Clostridiales; f__Lachnospiraceae; g__; s__
aae4c4ed528ae16ec417df816699cfdb	0.13783168	k__Bacteria; p__Firmicutes; c__Clostridia; o__Clostridiales; f__Ruminococcaceae; g__; s__
X9ec111134b7f857a1d270b470fbf331e	0.13374649	k__Bacteria; p__Proteobacteria; c__Epsilonproteobacteria; o__Campylobacterales; f__Helicobacteraceae; g__Flexispira; s__
ada812eec11bae7b45c860340eb24065	0.127817819	k__Bacteria; p__Actinobacteria; c__Coriobacteriia; o__Coriobacteriales; f__Coriobacteriaceae; g__; s__
c11b67d8c052cb2b8eaae7e9bf30d1f9	0.11004998	k__Bacteria; p__Firmicutes; c__Clostridia; o__Clostridiales; f__; g__; s__
X6498699e79b7aca5e5bdbdf89ca330d3	0.109798873	k__Bacteria; p__Bacteroidetes; c__Bacteroidia; o__Bacteroidales; f__S24-7; g__; s__
c5c739d7f511d6d9c30c24225ae14254	0.103870202	k__Bacteria; p__Firmicutes; c__Clostridia; o__Clostridiales; f__[Mogibacteriaceae]; g__; s__
d0b538c235318a5baa582e70517058e5	0.103870202	k__Bacteria; p__Actinobacteria; c__Coriobacteriia; o__Coriobacteriales; f__Coriobacteriaceae; g__; s__
X0df6c802966e8670279671824da4f10a	0.095485863	k__Bacteria; p__Firmicutes; c__Bacilli; o__Lactobacillales; f__Lactobacillaceae; g__Lactobacillus; s__
X61db99aabf206a639dee17ca130833de	0.095485863	k__Bacteria; p__Firmicutes; c__Clostridia; o__Clostridiales; f__Clostridiaceae; g__Clostridium; s__butyricum
X43a9a464782c24cc71180a49ef19830e	0.086102362	k__Bacteria; p__Firmicutes; c__Clostridia; o__Clostridiales
X0c8074dbf6ae2ae3b39298e14d306f07	0.086102362	k__Bacteria; p__Actinobacteria; c__Coriobacteriia; o__Coriobacteriales; f__Coriobacteriaceae; g__; s__
b1d8e09561b600103cc3d8b388c02478	0.086102362	k__Bacteria; p__Firmicutes; c__Clostridia; o__Clostridiales; f__Lachnospiraceae
X43b6707d39d84b5e1d8ece7375bd8390	0.086102362	k__Bacteria; p__Firmicutes; c__Clostridia; o__Clostridiales; f__; g__; s__
d8a42db5c61062f2a7b11fcdf03a65d5	0.086102362	k__Bacteria; p__Firmicutes; c__Clostridia; o__Clostridiales; f__Lachnospiraceae
X59b961d6271b069ab40420ecdbe35e0c	0.074329003	k__Bacteria; p__Firmicutes; c__Clostridia; o__Clostridiales; f__Ruminococcaceae; g__; s__
X950f3856394e951bc01742ec6f39e11c	0.074329003	k__Bacteria; p__Firmicutes; c__Clostridia; o__Clostridiales; f__Ruminococcaceae; g__; s__
c4098d21f8e840f7a6a8b586984f6ace	0.071538246	k__Bacteria; p__Bacteroidetes; c__Bacteroidia; o__Bacteroidales; f__Prevotellaceae; g__Prevotella; s__
b726627e18f29e0da5289f81ca294f92	0.071538246	k__Bacteria; p__Firmicutes; c__Clostridia; o__Clostridiales; f__Ruminococcaceae
c48070f3061b086b60ff32f77e0002fa	0.071538246	k__Bacteria; p__Firmicutes; c__Clostridia; o__Clostridiales; f__Ruminococcaceae; g__Faecalibacterium; s__prausnitzii

X68df6de5da6aefa331747280edcbedba	0.070592583	k__Bacteria; p__Actinobacteria; c__Coriobacteriia; o__Coriobacteriales; f__Coriobacteriaceae; g__Collinsella; s__
X36f8822030b4f2931d27025d7574db4d	0.070592583	k__Bacteria; p__Firmicutes; c__Clostridia; o__Clostridiales; f__Ruminococcaceae; g__; s__
X33b71b63b791314abbf6af5bb0c68bf0	0.052824743	k__Bacteria; p__Bacteroidetes; c__Bacteroidia; o__Bacteroidales; f__S24-7; g__; s__
X3525e8f55cd3355cc02bb9df648623e2	0.046896072	k__Bacteria; p__Firmicutes; c__Clostridia; o__Clostridiales; f__Lachnospiraceae; g__Blautia; s__producta
a7fe69d61cb22afa7516c62655f3db91	0.044733797	k__Bacteria; p__Tenericutes; c__Mollicutes; o__RF39; f__; g__; s__
X67a27018f69d64ed3d51d08fa76e75aa	0.042887264	k__Bacteria; p__Proteobacteria; c__Betaproteobacteria; o__Burkholderiales; f__Alcaligenaceae; g__Sutterella; s__
X2e4f2b53b856c4def6d021d01f5abb70	0.038260627	k__Bacteria; p__Firmicutes; c__Clostridia; o__Clostridiales; f__Lachnospiraceae; g__[Ruminococcus]; s__gnavus
X6fdb85b72abc94a4a93a9f2bd9294718	0.038260627	k__Bacteria; p__Firmicutes; c__Clostridia; o__Clostridiales; f__Lachnospiraceae; g__; s__
X5a729139b46885a340c039ce4294ef6f	0.036958593	k__Bacteria; p__Firmicutes; c__Clostridia; o__Clostridiales; f__Ruminococcaceae; g__; s__
X2ac3c8f867f239f644664796efde1be1	0.032331956	k__Bacteria; p__Firmicutes; c__Clostridia; o__Clostridiales; f__Christensenellaceae; g__; s__
e48ce90f2625cce75a6d5d671cadf8ea	0.032331956	k__Bacteria; p__Firmicutes; c__Erysipelotrichi; o__Erysipelotrichales; f__Erysipelotrichaceae; g__RFN20; s__
X796c6e6a817580f165bce51fa5c8fcd6	0.032331956	k__Bacteria; p__Proteobacteria; c__Deltaproteobacteria; o__Desulfovibrionales; f__Desulfovibrionaceae; g__Desulfovibrio; s__
X13b6cb1c2c3697ec16865c2737a4bbb6	0.032331956	k__Bacteria; p__Tenericutes; c__Mollicutes; o__RF39; f__; g__; s__
c8da1c24e8d4e84427e8a994413d7e06	0.032331956	k__Bacteria; p__Proteobacteria; c__Deltaproteobacteria; o__GMD14H09; f__; g__; s__
X20a68b281025ba8f8addfffca22b306a	0.032331956	k__Bacteria; p__Bacteroidetes; c__Bacteroidia; o__Bacteroidales; f__Prevotellaceae; g__Prevotella; s__
X43de2d22c3f82423071816f91be76df7	0.032331956	k__Bacteria; p__Firmicutes; c__Clostridia; o__Clostridiales; f__Lachnospiraceae; g__Coprococcus; s__
X9c1ec2bcd613366802912577c147ec9d	0.032331956	k__Bacteria; p__Firmicutes; c__Clostridia; o__Clostridiales; f__Ruminococcaceae; g__; s__
X610d01f22168e220c6669dda64cf4031	0.032331956	k__Bacteria; p__Firmicutes; c__Clostridia; o__Clostridiales; f__Clostridiaceae
X67e01a89cf3eabe01c14f7f937534cf4	0.028375368	k__Bacteria; p__Firmicutes; c__Clostridia; o__Clostridiales; f__Ruminococcaceae; g__; s__
d998dc427dd8fe3ffaf45cda17e95ed0	0.024290179	k__Bacteria; p__Bacteroidetes; c__Bacteroidia; o__Bacteroidales; f__[Paraprevotellaceae]; g__; s__
X5c4563e3dd7e12dcdf279783af929eaa	0.021037287	k__Bacteria; p__Firmicutes; c__Clostridia; o__Clostridiales; f__Lachnospiraceae; g__Roseburia; s__faecis
X41f9dd2615521de4773610fc548b4ecf	0.018123205	k__Bacteria; p__Bacteroidetes; c__Bacteroidia; o__Bacteroidales; f__[Paraprevotellaceae]; g__; s__
bc29605ee2f490499f5eb3a724b29a91	0.014564116	k__Archaea; p__Euryarchaeota; c__Methanobacteria; o__Methanobacteriales; f__Methanobacteriaceae; g__Methanosphaera; s__
c0091116b0a00327058bc7d9e362647e	0.014564116	k__Bacteria; p__Bacteroidetes; c__Bacteroidia; o__Bacteroidales; f__Prevotellaceae; g__; s__
f6ad9cc7413123d17e49a62b3ad9c79a	0.014564116	k__Bacteria; p__Bacteroidetes; c__Bacteroidia; o__Bacteroidales; f__Prevotellaceae; g__Prevotella; s__

X00a96fbd0ac34bfd245f9c24f8737f7d	0.014564116	k__Bacteria; p__Firmicutes; c__Clostridia; o__Clostridiales; f__Lachnospiraceae; g__Blautia; s__obeum
X2de0f958e30d26f04cba8d2a920bbe46	0.014564116	k__Bacteria; p__Firmicutes; c__Clostridia; o__Clostridiales; f__Lachnospiraceae; g__Blautia; s__
b621d4b3cca1ec89ceca12efca8a94fb	0.014564116	k__Bacteria; p__Firmicutes; c__Clostridia; o__Clostridiales; f__; g__; s__
X1d8abf4fd570ea8cb6629871ed3f397a	0.014564116	k__Bacteria; p__Actinobacteria; c__Coriobacteriia; o__Coriobacteriales; f__Coriobacteriaceae; g__Slackia; s__
X2e84fcb567e4b042779385f4a4f7ff84	0.0143527	k__Bacteria; p__Verrucomicrobia; c__Verruco-5; o__WCHB1-41; f__RFP12; g__; s__
X0b4aeb45916a24fd10e3655fe6481966	0.013811252	k__Bacteria; p__Firmicutes; c__Clostridia; o__Clostridiales; f__Christensenellaceae; g__; s__
d76d59ec71de0e3b22da0c9cd564d41a	0.01055836	k__Bacteria; p__Firmicutes; c__Clostridia; o__Clostridiales; f__Lachnospiraceae
X527bbcd459c37296ee8a6362156c2e4e	0.01055836	k__Bacteria; p__Firmicutes; c__Clostridia; o__Clostridiales; f__Ruminococcaceae
bc979a8588f5a2b031514a23cf3971cb	0.01055836	k__Bacteria; p__Firmicutes; c__Clostridia; o__Clostridiales; f__Ruminococcaceae; g__; s__
X5c4ec55ee0033be2594e104ef107c52c	0.00647317	k__Bacteria; p__Bacteroidetes; c__Bacteroidia; o__Bacteroidales; f__; g__; s__
b7d858871c56b4130c6556ce3f29fd20	0.00647317	k__Bacteria; p__Bacteroidetes; c__Bacteroidia; o__Bacteroidales; f__Prevotellaceae; g__Prevotella; s__copri
X1dde73d06b595083e0a9b1e6cbb1e9dd	0.006166974	k__Bacteria; p__Proteobacteria; c__Epsilonproteobacteria; o__Campylobacterales; f__Helicobacteraceae; g__Flexispira; s__
e506d4d9386a8c9954e2a828ffa44098	0.006166974	k__Bacteria; p__Firmicutes; c__Clostridia; o__Clostridiales; f__Ruminococcaceae; g__; s__
X90af3675c266d09bb7986147ac5885ec	0.005916389	k__Bacteria; p__Firmicutes; c__Clostridia; o__Clostridiales; f__Christensenellaceae; g__; s__
X4d3f1d8f02b45e845e419a273f5b2386	0.004626637	k__Bacteria; p__Firmicutes; c__Clostridia; o__Clostridiales; f__Ruminococcaceae; g__Ruminococcus; s__
X44396f15b10f6577d61a11c6047c939f	0.004626637	k__Archaea; p__Euryarchaeota; c__Methanobacteria; o__Methanobacteriales; f__Methanobacteriaceae; g__Methanobrevibacter; s__
a47817d5257ecf832986bfd05546dc23	0.004626637	k__Bacteria; p__Lentisphaerae; c__[Lentisphaeria]; o__Victivallales; f__Victivallaceae; g__Victivallis; s__vadensis
X2cf3a42dfa118cabf280462b35cdeda5	0.004626637	k__Bacteria; p__Firmicutes; c__Erysipelotrichi; o__Erysipelotrichales; f__Erysipelotrichaceae; g__L7A_E11; s__
f663dd604f793f22ce20452b09494608	0.004626637	k__Bacteria; p__Firmicutes; c__Clostridia; o__Clostridiales; f__Ruminococcaceae; g__; s__
c42c33e86e7b14aa0a09a65aaabbb9df	0.004626637	k__Bacteria; p__Bacteroidetes; c__Bacteroidia; o__Bacteroidales; f__S24-7; g__; s__
X0c0089fe5e474c353b6e2496d999bfe8	0.004626637	k__Bacteria; p__Firmicutes; c__Clostridia; o__Clostridiales; f__Ruminococcaceae; g__Ruminococcus; s__
X16530e5b50b780018210dac05a471715	0.004085189	k__Bacteria; p__Firmicutes; c__Clostridia; o__Clostridiales; f__Ruminococcaceae; g__Oscillospira; s__
b3bd13f0c98ac2d4153dfd73ab56dd2a	0.004085189	k__Bacteria; p__Bacteroidetes; c__Bacteroidia; o__Bacteroidales; f__[Paraprevotellaceae]; g__; s__
b7d0596261d747c1ac8e1c8b3dca415c	0.004085189	k__Bacteria; p__Firmicutes; c__Clostridia; o__Clostridiales; f__; g__; s__

e40410eaa791ef4386cc63b3d2b3b399	0.004085189	k__Bacteria; p__Firmicutes; c__Erysipelotrichi; o__Erysipelotrichales; f__Erysipelotrichaceae; g__RFN20; s__
X0d53bc80a23106375719154fbb0ab48e	0.002790758	k__Bacteria; p__Firmicutes; c__Clostridia; o__Clostridiales; f__Ruminococcaceae; g__Faecalibacterium; s__prausnitzii
b38de8a8c802a4845cc9e30cba6615f5	0.002790758	k__Bacteria; p__Firmicutes; c__Clostridia; o__Clostridiales; f__Ruminococcaceae; g__; s__
e1533fa00a73126001596fdb42776e27	0.002790758	k__Bacteria; p__Firmicutes; c__Clostridia; o__Clostridiales; f__Ruminococcaceae; g__; s__
X2bb806f05a014ee564ccf0fcae778c80	0.002790758	k__Bacteria; p__Bacteroidetes; c__Bacteroidia; o__Bacteroidales; f__S24-7; g__; s__

Supplementary Table2. Sheet7. Key taxa in Mountain Gorilla identified based on the network hub score.

ASV	HubScore	Taxa
X4b487f6998644c9ab8261cb84388db22	1	k__Fungi;p__unidentified;c__unidentified;o__unidentified;f__unidentified;g__unidentified;s__unidentified
cd2dfd32022679063a3b38238b54fac2	0.93756451	k__Fungi;p__Basidiomycota;c__Agaricomycetes;o__Polyporales;f__Ganodermataceae;g__Ganoderma
X0446575d89fb3c528f4c6695488dc860	0.88978248	k__Fungi;p__unidentified;c__unidentified;o__unidentified;f__unidentified;g__unidentified;s__unidentified
d0cc6c33b9a2c6f77552aeb0bcd46181	0.55317453	k__Fungi;p__unidentified;c__unidentified;o__unidentified;f__unidentified;g__unidentified;s__unidentified
ede5c6111ea13ccf365bd218ab4ba3a7	0.53972004	k__Fungi
X3d11b973c1db04a364bcbaf281b86a2c	0.47070304	k__Fungi;p__unidentified;c__unidentified;o__unidentified;f__unidentified;g__unidentified;s__unidentified
X82c161da92b677d86da5706f3964019c	0.39087182	k__Fungi
c4ea277d6b5537b7319797af3d804a33	0.34713121	k__Fungi
X9244f68348642fc5fcde1f080e2c2cb0	0.2948453	k__Fungi;p__unidentified;c__unidentified;o__unidentified;f__unidentified;g__unidentified;s__unidentified
X5e52c227876eaf11960205333760c501	0.29293827	k__Fungi;p__Ascomycota;c__Dothideomycetes;o__Pleosporales;f__Didymellaceae
X99fc3b75ffa5aab60a928ded1a0bd77b	0.28257619	k__Fungi;p__Ascomycota;c__Sordariomycetes;o__Xylariales;f__Xylariaceae;g__Xylaria;s__Xylaria_hypoxylon
X6863a73b09aedd995b88bf0cd879978d	0.26117602	k__Bacteria; p__Actinobacteria; c__Coriobacteriia; o__Coriobacteriales; f__Coriobacteriaceae; g__; s__
X0758ff38c519b6337645bb62461c9511	0.24359102	k__Bacteria; p__Firmicutes; c__Clostridia; o__Clostridiales; f__Ruminococcaceae; g__; s__
X44396f15b10f6577d61a11c6047c939f	0.24345542	k__Archaea; p__Euryarchaeota; c__Methanobacteria; o__Methanobacteriales; f__Methanobacteriaceae; g__Methanobrevibacter; s__
X79ba9fd704fc7264b695cc0504c337a9	0.24042686	k__Bacteria; p__Firmicutes; c__Clostridia; o__Clostridiales
c5673c320e2c60580f945fd93295033a	0.21425294	Unassigned
X9d4fe70552215a411cfb0a4497a4b985	0.20485578	k__Bacteria; p__Actinobacteria; c__Coriobacteriia; o__Coriobacteriales; f__Coriobacteriaceae; g__; s__
e70aac52b4b9557c354927fe02c01de3	0.20087936	k__Bacteria; p__Chloroflexi; c__Anaerolineae; o__Anaerolineales; f__Anaerolinaceae; g__SHD-231; s__
cadace978f08cbdfdf018a73f46e50b8	0.19893788	k__Bacteria; p__Actinobacteria; c__Coriobacteriia; o__Coriobacteriales; f__Coriobacteriaceae; g__Adlercreutzia; s__
ddb4a8335c1975a583b6a789278a7f4f	0.197409	k__Bacteria; p__Firmicutes; c__Clostridia; o__Clostridiales
X7c1133becb9a94483cd039df023a81e6	0.18783297	k__Fungi
b4ff137b6fbd7dd581a5fe40958fb08e	0.18734463	k__Fungi
X5c60ae000f7160d4d7edfe6a1498e009	0.18696314	k__Fungi;p__unidentified;c__unidentified;o__unidentified;f__unidentified;g__unidentified;s__unidentified
f6d07d1918010fa58f6f5473555fb2f7	0.17828636	k__Bacteria; p__Firmicutes; c__Clostridia; o__Clostridiales; f__Lachnospiraceae
X840f1ab652ea9d175444d94d511cbb89	0.17417602	k__Bacteria; p__Bacteroidetes; c__Bacteroidia; o__Bacteroidales
X31815356c56406ecf0078be269d31906	0.16422345	k__Bacteria; p__Bacteroidetes; c__Bacteroidia; o__Bacteroidales; f__Prevotellaceae; g__; s__

c2a2267cf10496a25b7d0761f5dff17c	0.15917431	k__Bacteria; p__Firmicutes; c__Clostridia; o__Clostridiales; f__Ruminococcaceae; g__Ruminococcus
abef6dd0c5aa07bd468f04d4bfea2a8c	0.15582571	k__Bacteria; p__Firmicutes; c__Clostridia; o__Clostridiales; f__Lachnospiraceae; g__; s__
X2690b25aaeca8be19ac7b1d6dc1d85c8	0.15312791	k__Bacteria; p__Spirochaetes; c__Spirochaetes; o__Spirochaetales; f__Spirochaetaceae; g__Treponema; s__
ba69b8ab8a667d87dc5e1f4ac1fe9f9a	0.15282727	k__Bacteria; p__Firmicutes; c__Clostridia; o__Clostridiales; f__[Mogibacteriaceae]; g__; s__
e12f9d4e13e6e2f2d15aee4713f1be1b	0.14485126	k__Bacteria; p__Verrucomicrobia; c__Verruco-5; o__WCHB1-41; f__RFP12; g__; s__
X0fe43da51e29bb42c4839f827b42d6b6	0.14320672	k__Bacteria; p__Spirochaetes; c__Spirochaetes; o__Sphaerochaetales; f__Sphaerochaetaceae; g__Sphaerochaeta; s__
b2c87bc6d57953af4bfd1b4647e8ef8b	0.14085905	k__Bacteria; p__Actinobacteria; c__Coriobacteriia; o__Coriobacteriales; f__Coriobacteriaceae; g__Adlercreutzia; s__
ff76b6c4fd74017cec052e8a84fb2d3a	0.14060861	k__Bacteria; p__Firmicutes; c__Clostridia; o__Clostridiales; f__Lachnospiraceae; g__; s__
db7f42d2a4ded289e177bb4c00edff61	0.13866383	k__Bacteria; p__Firmicutes; c__Clostridia; o__Clostridiales; f__Lachnospiraceae; g__; s__
X543eb306ab15d1b00b591205bb5b62a5	0.13762236	k__Fungi;p__Ascomycota;c__Sordariomycetes;o__Xylariales;f__Sporocadaceae;g__Neopestalotiopsis;s__Neopestalotiopsis_foedans
bc979a8588f5a2b031514a23cf3971cb	0.13725996	k__Bacteria; p__Firmicutes; c__Clostridia; o__Clostridiales; f__Ruminococcaceae; g__; s__
bd9a9034e5e3bdb341ee33a909d4ee09	0.13725996	k__Bacteria; p__Firmicutes; c__Clostridia; o__Clostridiales; f__Lachnospiraceae; g__; s__
d2ae019a0fa9e489ed9cfc2ae0629c8f	0.13725996	k__Bacteria; p__Verrucomicrobia; c__Verruco-5; o__WCHB1-41; f__RFP12; g__; s__
e0652aed13d278f0e66e62e50360c4ab	0.13725996	k__Bacteria; p__Firmicutes; c__Clostridia; o__Clostridiales; f__Lachnospiraceae; g__Pseudobutyrivibrio; s__
X240776793aa237ffef655f53b4a7a690	0.13725996	k__Bacteria; p__Firmicutes; c__Clostridia; o__Clostridiales; f__Lachnospiraceae; g__Clostridium; s__lavalense
fa15385fd4e428c61d7459be496fd83b	0.13671366	k__Bacteria; p__TM7; c__TM7-3; o__CW040; f__F16; g__; s__
X467669415f8967eefc7943baa29b244b	0.13671366	k__Bacteria; p__Tenericutes; c__Mollicutes; o__Mycoplasmatales; f__Mycoplasmataceae; g__; s__
d8f45fe6e9250729ae9b8b534db7c1a8	0.12841075	k__Archaea; p__Euryarchaeota; c__Thermoplasmata; o__E2; f__[Methanomassiliicoccaceae]; g__; s__
a118d212b798d130e0eca5026fa3a1e9	0.11959011	k__Bacteria; p__Proteobacteria; c__Deltaproteobacteria; o__Desulfovibrionales; f__Desulfovibrionaceae; g__Desulfovibrio; s__D168
X7f20483cee471b11108b1dbed82cb33f	0.11830389	k__Bacteria; p__Firmicutes; c__Clostridia; o__Clostridiales; f__Lachnospiraceae; g__Blautia; s__
X9c7bb6afb72b7c69c5872c14e860d8a7	0.11608024	k__Bacteria; p__Firmicutes; c__Clostridia; o__Clostridiales; f__Lachnospiraceae; g__; s__
X1c06f26cc258f7b8a75e67c1de7ea6b8	0.11479931	Unassigned
X29cb1bde52f4a1799048265bda201748	0.11479931	k__Bacteria; p__Firmicutes; c__Clostridia; o__Clostridiales; f__Lachnospiraceae
X73f98259afce62b1d1a12ba85685080d	0.10763891	k__Fungi;p__unidentified;c__unidentified;o__unidentified;f__unidentified;g__unidentified;s__unidentified
X42140b89d9315b542313bc212474ed44	0.10565332	k__Bacteria; p__Actinobacteria; c__Coriobacteriia; o__Coriobacteriales; f__Coriobacteriaceae; g__; s__
e2ae3dca607ac3d0c41521221aa6d4b3	0.10113195	k__Bacteria; p__Firmicutes; c__Erysipelotrichi; o__Erysipelotrichales; f__Erysipelotrichaceae; g__; s__
X1599dc1cc5a6f06363fcbb4ac4251e77	0.09919185	k__Bacteria; p__Firmicutes; c__Clostridia; o__Clostridiales; f__Ruminococcaceae; g__; s__

X794cc1c6386320420a2c2ea76eedaaf8	0.09454194	k__Bacteria; p__Firmicutes; c__Clostridia; o__Clostridiales; f__Ruminococcaceae; g__; s__
X26e7ed2b3c2fca3c58b85d81d4e31c33	0.08855605	k__Fungi;p__unidentified;c__unidentified;o__unidentified;f__unidentified;g__unidentified;s__unidentified
X2a11904576933ec08ff762ebabeb767e	0.08247264	k__Fungi;p__unidentified;c__unidentified;o__unidentified;f__unidentified;g__unidentified;s__unidentified
e3cdda80619a877c71570fe8812129f9	0.07995223	k__Bacteria; p__Firmicutes; c__Clostridia; o__Clostridiales; f__[Mogibacteriaceae]; g__; s__
X6376ea6dab7d0fde3cd66f53b57e1484	0.07919217	k__Bacteria; p__Proteobacteria; c__Gammaproteobacteria; o__Pseudomonadales; f__Pseudomonadaceae; g__Pseudomonas; s__
X3c4b2dbf7e88a26dcbe537a0f067596f	0.07558588	k__Bacteria; p__Firmicutes; c__Clostridia; o__Clostridiales; f__Lachnospiraceae
b7ba47698ed504b740e9348aa950a544	0.07419612	k__Bacteria; p__Firmicutes; c__Clostridia; o__Clostridiales; f__Ruminococcaceae; g__; s__
da591c5d4bb1ce9587ac15167a0b87a0	0.07419612	k__Bacteria; p__Firmicutes; c__Clostridia; o__Clostridiales; f__Ruminococcaceae; g__Faecalibacterium; s__prausnitzii
X619d1441d6d638828685513300f6d641	0.05804236	k__Bacteria; p__Firmicutes; c__Clostridia; o__Clostridiales; f__Ruminococcaceae; g__; s__
a81f6cb76f072e5c9bb669ceeb52390b	0.0579044	k__Fungi;p__Basidiomycota;c__Agaricomycetes;o__Agaricales;f__Psathyrellaceae;g__Coprinellus;s__Coprinellus_disseminatus
a863aafd8bc588060b8c70de3227294e	0.0561017	k__Bacteria; p__Firmicutes; c__Clostridia; o__Clostridiales; f__Ruminococcaceae; g__; s__
e6c95d620576f6503d607275d777bef1	0.05022264	k__Archaea; p__Euryarchaeota; c__Thermoplasmata; o__E2; f__[Methanomassiliicoccaceae]; g__; s__
a964478144e83d41e8216b4cab2aea10	0.04809967	k__Bacteria; p__Proteobacteria; c__Betaproteobacteria
a3e92cdf515f0a04cdb21c6bf06cdece	0.04539179	k__Archaea; p__Euryarchaeota; c__Methanobacteria; o__Methanobacteriales; f__Methanobacteriaceae; g__Methanosphaera; s__
X785d65c6f2f55ace657add335783f516	0.0410264	k__Bacteria; p__Firmicutes; c__Clostridia; o__Clostridiales; f__[Mogibacteriaceae]; g__; s__
d205c635885fb65b4021537622356e3c	0.04094352	k__Fungi
aac76587f2b3cba87cf266dcb41e3641	0.03806811	k__Bacteria; p__Firmicutes; c__Erysipelotrichi; o__Erysipelotrichales; f__Erysipelotrichaceae; g__Bulleidia
X18bb09f33338fb287bf1cfe4acd5ca43	0.037785	k__Bacteria; p__Actinobacteria; c__Coriobacteriia; o__Coriobacteriales; f__Coriobacteriaceae; g__; s__
ac8e57fd9b2d097165cbd93c4ab83ecd	0.03612801	k__Bacteria; p__Firmicutes; c__Clostridia; o__Clostridiales; f__Ruminococcaceae; g__Gemmiger; s__formicilis
ff0c895a80b4184dea0356c214971e3a	0.03612801	k__Bacteria; p__Tenericutes; c__Mollicutes; o__RF39; f__; g__; s__
X0266597168ff0f8a0af449abe4599b0c	0.03612801	k__Bacteria; p__Verrucomicrobia; c__Verruco-5; o__WCHB1-41; f__RFP12; g__; s__
X1e735e32a159cd305a3ab49b65182812	0.03612801	k__Bacteria; p__Firmicutes; c__Clostridia; o__Clostridiales; f__Ruminococcaceae; g__; s__
X7baae0d5fced0473d9b27d518c762646	0.03457549	k__Fungi;p__unidentified;c__unidentified;o__unidentified;f__unidentified;g__unidentified;s__unidentified
d8fbb1502286d84016871435218dabfb	0.0320151	k__Fungi;p__unidentified;c__unidentified;o__unidentified;f__unidentified;g__unidentified;s__unidentified
b60ea01d9efc02c2ebd084f37e679603	0.03154753	k__Fungi;p__Basidiomycota;c__Malasseziomycetes;o__Malasseziales;f__Malasseziaceae;g__Malassezia;s__Malassezia_restricta
X1b672a28fff72c4817820c82c8a63219	0.03058554	k__Bacteria; p__Firmicutes; c__Clostridia; o__Clostridiales; f__; g__; s__
e0cdef7457817f32e98b7ff57e7b5d26	0.02556813	k__Bacteria; p__Bacteroidetes; c__Bacteroidia; o__Bacteroidales

X1d17970ef050ad232d4bdb526d5199c6	0.02460416	k__Fungi;p__unidentified;c__unidentified;o__unidentified;f__unidentified;g__unidentified;s__unidentified
X2d700ed4c9f0b7db34a6604af085741a	0.02447644	k__Bacteria; p__Bacteroidetes; c__Bacteroidia; o__Bacteroidales; f__[Paraprevotellaceae]; g__[Prevotella]; s__
f4687285ab01e29bf37cde777678687f	0.02246065	k__Bacteria; p__Bacteroidetes; c__Bacteroidia; o__Bacteroidales; f__; g__; s__
f6be8fd3434273e20c0b5a1461ee3c85	0.02246065	k__Bacteria; p__Firmicutes; c__Clostridia; o__Clostridiales; f__; g__; s__
X0eba36394b3b0935266fc09e7c3d6eb5	0.02246065	k__Bacteria; p__Spirochaetes; c__Spirochaetes; o__Spirochaetales; f__Spirochaetaceae; g__Treponema; s__
X7327a55af477aa89dc73611f2806c3e9	0.02246065	k__Bacteria; p__Firmicutes; c__Clostridia; o__Clostridiales; f__Ruminococcaceae; g__; s__
X8db0cc8463bf7cddcda37e038edcb1bc	0.02246065	k__Bacteria; p__Lentisphaerae; c__[Lentisphaeria]; o__Z20; f__R4-45B; g__; s__
X2d2b904b13603efa7a0bfd708302a676	0.02191436	k__Bacteria; p__Firmicutes; c__Clostridia; o__Clostridiales
fd9604431c04c520809e157ab0a26a8f	0.01959828	k__Bacteria; p__Actinobacteria; c__Coriobacteriia; o__Coriobacteriales; f__Coriobacteriaceae; g__; s__
X2553ed5072c6f97741e17feaaad9aec4	0.01727452	k__Bacteria; p__Bacteroidetes; c__Bacteroidia; o__Bacteroidales; f__; g__; s__
X7399d67fa79f8849916f3f34caa5f63f	0.01587064	k__Bacteria; p__Tenericutes; c__Mollicutes; o__RF39; f__; g__; s__
X70ecf3f1d0cee3c5c8e704e99eef8e3e	0.01523341	k__Bacteria; p__Bacteroidetes; c__Bacteroidia; o__Bacteroidales
X003d2f871db236415b4a4bcc8b0c829f	0.01282823	k__Fungi;p__unidentified;c__unidentified;o__unidentified;f__unidentified;g__unidentified;s__unidentified
X7a313870bb97fff943b4b91400a6b835	0.01197166	k__Bacteria; p__Firmicutes; c__Erysipelotrichi; o__Erysipelotrichales; f__Erysipelotrichaceae; g__p-75-a5; s__
X2abb85ea251f01e94623408e600f934b	0.01147349	k__Bacteria; p__Proteobacteria; c__Deltaproteobacteria; o__Desulfovibrionales; f__Desulfovibrionaceae; g__Desulfovibrio; s__D168
X68b655ce0b3de7da0db0b3b6869b440d	0.00928906	k__Bacteria; p__Actinobacteria; c__Coriobacteriia; o__Coriobacteriales; f__Coriobacteriaceae; g__; s__
add9d4387a6350194317b01fafa5a680	0.00762662	k__Bacteria; p__Bacteroidetes; c__Bacteroidia; o__Bacteroidales; f__Porphyromonadaceae; g__Parabacteroides; s__
X233039daf52bf206a2558eede3c5fad6	0.00762662	k__Bacteria; p__Actinobacteria; c__Coriobacteriia; o__Coriobacteriales; f__Coriobacteriaceae; g__; s__
X847b2eb6121654af9059db00367fa4f9	0.00762662	k__Bacteria; p__Actinobacteria; c__Coriobacteriia; o__Coriobacteriales; f__Coriobacteriaceae; g__; s__
d0b716a1b345a39ec1fb098778fb4fab	0.00760679	k__Bacteria; p__Firmicutes; c__Clostridia; o__Clostridiales; f__[Mogibacteriaceae]; g__; s__
X24a2d03bd66addf61ae0d8b3dbb1145c	0.00760679	k__Bacteria; p__Firmicutes; c__Clostridia; o__Clostridiales; f__Lachnospiraceae; g__; s__
X31c0203284637c9382d689abc06a0d85	0.00760679	k__Bacteria; p__Firmicutes; c__Clostridia; o__Clostridiales; f__Ruminococcaceae; g__; s__
X3903585322b1fabd7ee0672e2dcf8701	0.0075913	k__Bacteria; p__Proteobacteria; c__Betaproteobacteria; o__Burkholderiales; f__Alcaligenaceae; g__Sutterella; s__
b5d9a9960cd0513b1c89e084b335c3d2	0.00359566	k__Bacteria; p__Firmicutes; c__Clostridia; o__Clostridiales; f__; g__; s__
cc7b0c4fe7613151aee46e9be3ea14a9	0.00359566	k__Bacteria; p__Firmicutes; c__Clostridia; o__Clostridiales; f__Ruminococcaceae; g__Oscillospira; s__
X026badcb181adb78fecbe14919ea4f12	0.00359566	k__Bacteria; p__Bacteroidetes; c__Bacteroidia; o__Bacteroidales; f__; g__; s__
X252ae349c56c501852b3cd61097190ed	0.0023511	k__Bacteria; p__Firmicutes; c__Clostridia; o__Clostridiales; f__Christensenellaceae; g__; s__
X2393b358b70b807e64b122d71982aaf3	0.00167352	k__Bacteria; p__Actinobacteria; c__Coriobacteriia; o__Coriobacteriales; f__Coriobacteriaceae; g__Collinsella; s__

X6a2b2a6def4ca2de81aa7719db83eeca	0.00140388	k__Bacteria; p__Firmicutes; c__Clostridia; o__Clostridiales; f__Lachnospiraceae; g__; s__
X8e73663f2154a81f8b7a40000f2d4b4c	0.00140388	k__Bacteria; p__Firmicutes; c__Clostridia; o__Clostridiales; f__Ruminococcaceae; g__Oscillospira; s__
X5f78d2e842250d6c48ff921006d0f9d7	0.00052087	k__Bacteria; p__Actinobacteria; c__Coriobacteriia; o__Coriobacteriales; f__Coriobacteriaceae; g__; s__
X241dab63b8c1f6ab83ead7a5142499bd	0.00027289	k__Fungi;p__unidentified;c__unidentified;o__unidentified;f__unidentified;g__unidentified;s__unidentified
b6e2c9c93b4dd7babcbf52038fa34b78	1.11E-05	k__Bacteria; p__Firmicutes; c__Clostridia; o__Clostridiales; f__; g__; s__
X5ad2e2d434beabfedd0ec753ad1a329b	1.11E-05	k__Bacteria; p__Fusobacteria; c__Fusobacteriia; o__Fusobacteriales; f__Fusobacteriaceae; g__; s__

Supplementary Table2. Sheet8. Key taxa in Western Lowland Gorilla identified based on the network hub score.

ASV	HubScore	Taxa
X35ffcc3b809d667286737d79670b8de5	1	k__Bacteria; p__Actinobacteria; c__Actinobacteria; o__Bifidobacteriales; f__Bifidobacteriaceae; g__Bifidobacterium
ff175bf248f825575b442007206b0a2c	0.84854616	k__Bacteria; p__Bacteroidetes; c__Bacteroidia; o__Bacteroidales; f__; g__; s__
a2d620b95b186edf72c0e73c452a30ae	0.78425976	k__Bacteria; p__Bacteroidetes; c__Bacteroidia; o__Bacteroidales; f__Prevotellaceae; g__Prevotella; s__
X3e1088a9b0a946fd0ce8e3621fb33e85	0.45506115	k__Bacteria; p__Firmicutes; c__Clostridia; o__Clostridiales
X0229a3ed44e215720e8926eb3b372933	0.45506115	k__Bacteria; p__Firmicutes; c__Erysipelotrichi; o__Erysipelotrichales; f__Erysipelotrichaceae; g__p-75-a5; s__
f65fe04b0921a77bacea7992c72b38d8	0.45506115	k__Bacteria; p__Firmicutes; c__Clostridia; o__Clostridiales; f__Christensenellaceae; g__; s__
ed13fbb347defb92256f9d5f8bcc5add	0.45506115	k__Bacteria; p__Firmicutes; c__Clostridia; o__Clostridiales; f__Ruminococcaceae; g__; s__
X8db0cc8463bf7cddcda37e038edcb1bc	0.45506115	k__Bacteria; p__Lentisphaerae; c__[Lentisphaeria]; o__Z20; f__R4-45B; g__; s__
c89de32164d066b9cee9de971d8db6e1	0.45506115	k__Bacteria; p__Tenericutes; c__Mollicutes; o__Anaeroplasmatales; f__Anaeroplasmataceae; g__; s__
X2a6665c89f80025ad1305e78f0bfdcb0	0.45506115	k__Bacteria; p__Firmicutes; c__Clostridia; o__Clostridiales; f__Lachnospiraceae
f5f247005f1314b5108bb61d4423cf2c	0.45506115	k__Bacteria; p__Firmicutes; c__Erysipelotrichi; o__Erysipelotrichales; f__Erysipelotrichaceae; g__RFN20; s__
adceb3dfb4863c4707a8a6d14db02cfe	0.45506115	k__Bacteria; p__Firmicutes; c__Clostridia; o__Clostridiales; f__Ruminococcaceae; g__; s__
X4335c4f074c4ce7600acb94b939057ec	0.44955339	k__Bacteria; p__Planctomycetes; c__Planctomycetia; o__Pirellulales; f__Pirellulaceae; g__; s__
X7e025abc1239c5550e3fe9610ff28d91	0.32919862	k__Bacteria; p__Tenericutes; c__Mollicutes; o__RF39; f__; g__; s__
X21586a45e85ce6bf1687fa668302365f	0.32919862	k__Bacteria; p__Firmicutes; c__Clostridia; o__Clostridiales; f__; g__; s__
X95636cde183c77c2a0109d48f419d158	0.32919862	k__Bacteria; p__Firmicutes; c__Clostridia; o__Clostridiales; f__; g__; s__
c47f43acb387a2fb571c452bc0c4be0e	0.32919862	k__Bacteria; p__Tenericutes; c__Mollicutes; o__RF39; f__; g__; s__
X2f6df61bac26d572d82137d547fdc7cf	0.32919862	k__Bacteria; p__Tenericutes; c__Mollicutes; o__RF39; f__; g__; s__
X7bc9a2e59b65fa37d751cee5f94c4a4f	0.32919862	k__Bacteria; p__Firmicutes; c__Clostridia; o__Clostridiales; f__[Mogibacteriaceae]; g__Mogibacterium; s__
f4687285ab01e29bf37cde777678687f	0.32919862	k__Bacteria; p__Bacteroidetes; c__Bacteroidia; o__Bacteroidales; f__; g__; s__

X48b421cf8b56e91becab03cbd246474e	0.32919862	k__Bacteria; p__Bacteroidetes; c__Bacteroidia; o__Bacteroidales; f__[Paraprevotellaceae]; g__; s__
d478910a683aff84bec4fb1213969719	0.24055677	k__Bacteria; p__Actinobacteria; c__Coriobacteriia; o__Coriobacteriales; f__Coriobacteriaceae; g__; s__
X7566bcaf1d779beeb23d386e833dab2a	0.23778692	k__Bacteria; p__Lentisphaerae; c__[Lentisphaeria]; o__Z20; f__R4-45B; g__; s__
a16930bcf789ac4fb2033e1bae65d393	0.21574024	k__Bacteria; p__Bacteroidetes; c__Bacteroidia; o__Bacteroidales; f__; g__; s__
X7023ef6e491364c659ce1f086354252c	0.21574024	k__Bacteria; p__Actinobacteria; c__Coriobacteriia; o__Coriobacteriales; f__Coriobacteriaceae; g__Collinsella; s__
a658a3950ddcd55b546e751550b6807e	0.21574024	k__Bacteria; p__Firmicutes; c__Erysipelotrichi; o__Erysipelotrichales; f__Erysipelotrichaceae; g__RFN20; s__
eaaf08a1870579ac02700c7bf82169cc	0.21574024	k__Bacteria; p__Firmicutes; c__Erysipelotrichi; o__Erysipelotrichales; f__Erysipelotrichaceae; g__p-75-a5; s__
X0ebcd9137c7542614095dfcd01905c96	0.12035477	k__Bacteria; p__Tenericutes; c__Mollicutes; o__RF39; f__; g__; s__
X847dd19dd00c43810f3051cf594a1a77	0.12035477	k__Bacteria
X3b951c1f21c92bc555de6b5c536eca3d	0.12035477	k__Bacteria; p__Firmicutes; c__Clostridia; o__Clostridiales; f__Ruminococcaceae; g__; s__
X33518e48b174ac428cb7be1a216c6c6e	0.12035477	k__Bacteria; p__Firmicutes; c__Clostridia; o__Clostridiales; f__Lachnospiraceae; g__Coprococcus; s__
bc979a8588f5a2b031514a23cf3971cb	0.02710471	k__Bacteria; p__Firmicutes; c__Clostridia; o__Clostridiales; f__Ruminococcaceae; g__; s__
e10c65b6d7afe6d6fab60b7c887d0262	0.02481654	k__Bacteria; p__Firmicutes; c__Clostridia; o__Clostridiales; f__Lachnospiraceae
X4174bf2de3b9e936974bf73289a8ae6c	0.02481654	k__Bacteria; p__Firmicutes; c__Clostridia; o__Clostridiales; f__Ruminococcaceae; g__; s__
X24a2d03bd66addf61ae0d8b3dbb1145c	0.02481654	k__Bacteria; p__Firmicutes; c__Clostridia; o__Clostridiales; f__Lachnospiraceae; g__; s__
c2a2d50669235f126fad4298df409c2c	0.02481654	k__Bacteria; p__Verrucomicrobia; c__Verruco-5; o__WCHB1-41; f__RFP12; g__; s__
X0eba36394b3b0935266fc09e7c3d6eb5	0.02204668	k__Bacteria; p__Spirochaetes; c__Spirochaetes; o__Spirochaetales; f__Spirochaetaceae; g__Treponema; s__
X17547c2f776585d1d76fb464c0b00c23	0.02204668	k__Bacteria; p__Proteobacteria; c__Epsilonproteobacteria; o__Campylobacterales; f__Campylobacteraceae; g__Campylobacter; s__
b2fb23a39d2283b55519332766d4c4ab	0.02204668	k__Bacteria; p__Verrucomicrobia; c__Verruco-5; o__WCHB1-41; f__RFP12; g__; s__
X4567ac24f23a8e96e9cbfdb56f7057c7	0.02204668	k__Bacteria; p__Bacteroidetes; c__Bacteroidia; o__Bacteroidales; f__; g__; s__
c04c87ff72eb8ad4ec07894d5b0cf2a7	0.00443917	Unassigned

X9370b6f66c750d3d42bbe22650b40a99	0.00228818	k__Bacteria; p__Firmicutes; c__Clostridia; o__Clostridiales; f__Ruminococcaceae
c1fa4eff2c73bc54ab6fc45c1e0f8caf	0.00183804	k__Bacteria; p__Tenericutes; c__Mollicutes; o__Anaeroplasmatales; f__Anaeroplasmataceae; g__; s__
b0ceeb1a9580e139ac1c42cf23f502ec	0.00108305	k__Bacteria; p__Firmicutes; c__Clostridia; o__Clostridiales; f__Lachnospiraceae; g__; s__
X71efbec757ca125dcb0e754137d9cc75	0.00075917	k__Bacteria; p__Bacteroidetes; c__Bacteroidia; o__Bacteroidales; f__; g__; s__
b38de8a8c802a4845cc9e30cba6615f5	0.00063463	k__Bacteria; p__Firmicutes; c__Clostridia; o__Clostridiales; f__Ruminococcaceae; g__; s__
X8387c8957cd0ec7b5f2b8e4cf1ebd0d0	0.00053391	k__Bacteria; p__Firmicutes; c__Clostridia; o__Clostridiales; f__Peptococcaceae; g__Peptococcus; s__
X8e8a0a64f033aae0b0302c80fd35b82a	0.00028752	k__Bacteria; p__Firmicutes; c__Clostridia; o__Clostridiales; f__; g__; s__
X252ae349c56c501852b3cd61097190ed	7.86E-05	k__Bacteria; p__Firmicutes; c__Clostridia; o__Clostridiales; f__Christensenellaceae; g__; s__
c6c3ab4e828fb40d6e05967b7aac9338	7.29E-05	k__Bacteria; p__Firmicutes; c__Clostridia; o__Clostridiales; f__Lachnospiraceae; g__Blautia; s__
X577478ea5568aa13923682d09991f152	7.29E-05	k__Bacteria; p__Actinobacteria; c__Coriobacteriia; o__Coriobacteriales; f__Coriobacteriaceae; g__; s__
X1fd72c9032729af96baca77cce0d67d5	7.29E-05	k__Bacteria; p__Actinobacteria; c__Coriobacteriia; o__Coriobacteriales; f__Coriobacteriaceae; g__; s__
X0ff6f32a812d98d9bd46bb3c7a6294cc	7.29E-05	k__Bacteria; p__Actinobacteria; c__Coriobacteriia; o__Coriobacteriales; f__Coriobacteriaceae; g__Adlercreutzia; s__
c0b1dbd7cff46a0e46798f6cf0b86c95	7.29E-05	k__Bacteria; p__Bacteroidetes; c__Bacteroidia; o__Bacteroidales; f__Prevotellaceae; g__Prevotella; s__stercorea
ff0c3058877b605f27f7558b773ee339	6.44E-05	k__Bacteria; p__Spirochaetes; c__Spirochaetes; o__Sphaerochaetales; f__Sphaerochaetaceae; g__Sphaerochaeta; s__
dc6b8f0e0f94076b835897991f6d4322	6.44E-05	k__Bacteria; p__Spirochaetes; c__Spirochaetes; o__Sphaerochaetales; f__Sphaerochaetaceae; g__Sphaerochaeta; s__
X03c51b5e257b8e286445b07fe1e7d80b	6.44E-05	k__Bacteria; p__Firmicutes; c__Clostridia; o__Clostridiales; f__Veillonellaceae; g__Dialister; s__
b707f86ae5986ddabf40a393b569d306	6.15E-06	k__Bacteria; p__Firmicutes; c__Clostridia; o__Clostridiales; f__Lachnospiraceae; g__Anaerostipes; s__
X288359d621a383c1e46f0c0418660242	1.49E-15	k__Fungi;p__Ascomycota;c__Dothideomycetes;o__Botryosphaeriales;f__Botryosphaeriaceae;g__Lasiodiplodia
a171d6b000917ac0e0a39cb0c8b2fc1b	6.35E-16	k__Fungi;p__Ascomycota;c__Dothideomycetes;o__Botryosphaeriales;f__Botryosphaeriaceae;g__Lasiodiplodia
X52cdc265412299f4b1520f751a1f185a	3.99E-16	k__Bacteria; p__Firmicutes; c__Clostridia; o__Clostridiales; f__Ruminococcaceae; g__Ruminococcus; s__flavefaciens

c489d3e41cd4004902f023b3c0d96178	2.77E-16	k__Bacteria; p__Actinobacteria
bb045933c26289f69da94dc8214151da	2.77E-16	k__Bacteria; p__Firmicutes; c__Erysipelotrichi; o__Erysipelotrichales; f__Erysipelotrichaceae; g__RFN20; s__
X31ac5d226982f55053457715c8fe524a	2.77E-16	k__Bacteria; p__Verrucomicrobia; c__Verruco-5; o__WCHB1-41; f__RFP12; g__; s__
X1b672a28fff72c4817820c82c8a63219	2.77E-16	k__Bacteria; p__Firmicutes; c__Clostridia; o__Clostridiales; f__; g__; s__
c06720adbf0bf0a25ca2dbd0facfc52d	2.77E-16	k__Bacteria; p__Bacteroidetes; c__Bacteroidia; o__Bacteroidales; f__; g__; s__
X49653229f8c1f6c9149ee61ed1ba36db	1.21E-16	k__Bacteria; p__Proteobacteria; c__Betaproteobacteria; o__Burkholderiales
bf3912133bb98bbb4d46d779886bb2c2	1.21E-16	k__Bacteria; p__Firmicutes; c__Clostridia; o__Clostridiales; f__Ruminococcaceae; g__; s__
X67a27018f69d64ed3d51d08fa76e75aa	1.21E-16	k__Bacteria; p__Proteobacteria; c__Betaproteobacteria; o__Burkholderiales; f__Alcaligenaceae; g__Sutterella; s__
a915ba59510cd0721f007689db26287b	1.02E-16	k__Fungi;p__unidentified;c__unidentified;o__unidentified;f__unidentified;g__unidentified;s__unidentified
f8f3af87f13963b734bb2b2710cf37ee	4.12E-17	k__Bacteria; p__Firmicutes; c__Clostridia; o__Clostridiales; f__Ruminococcaceae; g__; s__
a2f49b2ad0d2db2f52a7f4d435e45259	4.12E-17	k__Fungi;p__Ascomycota;c__Dothideomycetes;o__Pleosporales
X79751e6d6445f7bdb789b2b72df77205	3.78E-17	k__Bacteria; p__Firmicutes; c__Clostridia; o__Clostridiales; f__; g__; s__
X68bdcdd3d60e66e4154c1d015155cb69	3.78E-17	k__Bacteria; p__Bacteroidetes; c__Bacteroidia; o__Bacteroidales; f__RF16; g__; s__
X22ec27b7367ce45e8ac8e9a944a038a1	2.27E-17	k__Fungi;p__Ascomycota;c__Saccharomycetes;o__Saccharomycetales;f__Saccharomycetales_fam_Incertae_sedis;g__Candida;s__Candida_quercitrusa
f6d6ab0c61d1317105a443fbbaf5b891	1.14E-17	k__Bacteria; p__Firmicutes; c__Clostridia; o__Clostridiales; f__[Mogibacteriaceae]; g__; s__
ff76b6c4fd74017cec052e8a84fb2d3a	1.14E-17	k__Bacteria; p__Firmicutes; c__Clostridia; o__Clostridiales; f__Lachnospiraceae; g__; s__

Supplementary Table2. Sheet9. Key taxa in USA-Human identified based on the network hub score.

ASV	HubScore	Taxa
X1c9ff9e2aeeaebc0df06f1e182022bb7	1	k__Fungi;p__Ascomycota;c__Saccharomycetes;o__Saccharomycetales;f__Saccharomycetaceae;g__Saccharomyces
X742e1f191223f58590661de80b9d6e3b	0.14285714	k__Bacteria; p__Bacteroidetes; c__Bacteroidia; o__Bacteroidales; f__Rikenellaceae; g__Alistipes; s__onderdonkii
e335f74033bc634af43ee6baa84fa247	0.14285714	k__Bacteria; p__Firmicutes; c__Clostridia; o__Clostridiales; f__Peptostreptococcaceae; g__; s__
b15193fce14759d1c06728933e044af6	0.14285714	k__Bacteria; p__Bacteroidetes; c__Bacteroidia; o__Bacteroidales; f__Bacteroidaceae; g__Bacteroides; s__uniformis
X1b9ab7ba8573cbfe5f1a9f59f0b38ff4	0.14285714	k__Bacteria; p__Actinobacteria; c__Coriobacteriia; o__Coriobacteriales; f__Coriobacteriaceae; g__Adlercreutzia; s__
X0a68ec564e278b0246c920ad7de41880	0.14285714	k__Bacteria; p__Firmicutes; c__Clostridia; o__Clostridiales; f__Lachnospiraceae; g__Blautia; s__
X9e1f282914b07cd79711ce21e7cf4aff	0.14285714	k__Bacteria; p__Firmicutes; c__Clostridia; o__Clostridiales; f__Lachnospiraceae; g__Lachnospira; s__
f4cc6e4932f507b33d1335d801f54563	0.14285714	k__Bacteria; p__Firmicutes; c__Clostridia; o__Clostridiales; f__Lachnospiraceae; g__Dorea; s__formicigenerans

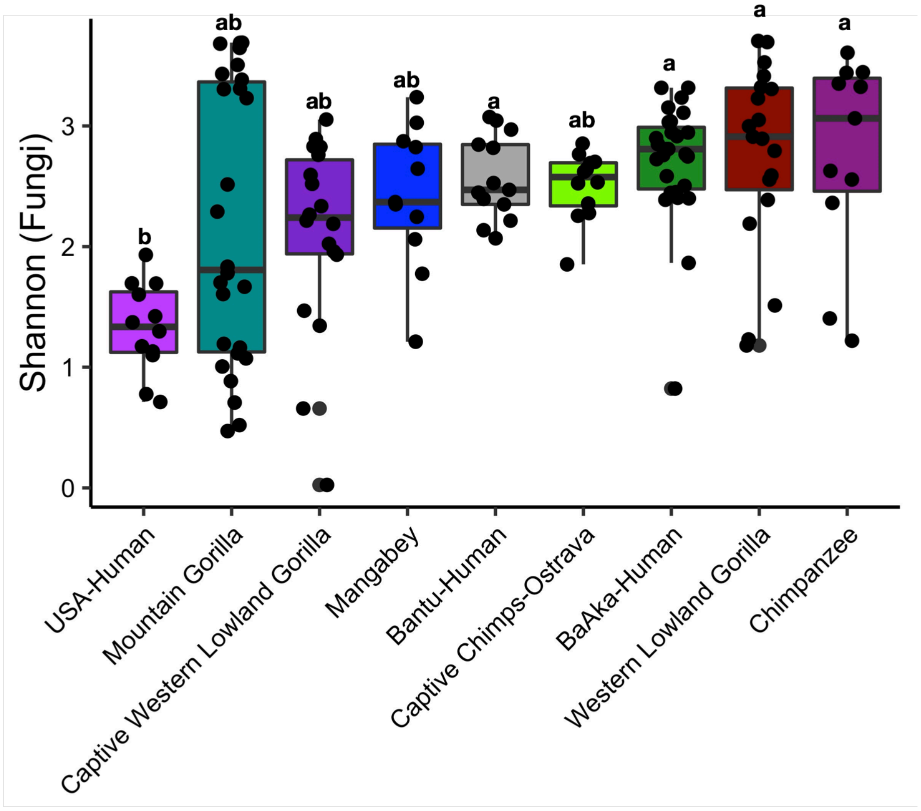

Supplementary Figure 1. Gut mycobiome differs based on subsistence strategies across different primates. Alpha diversity analysis showing lower fungal diversity in western humans as compared to other primate groups. Different letters denote significant differences according to FDR-adjusted Kruskal-Wallis tests.

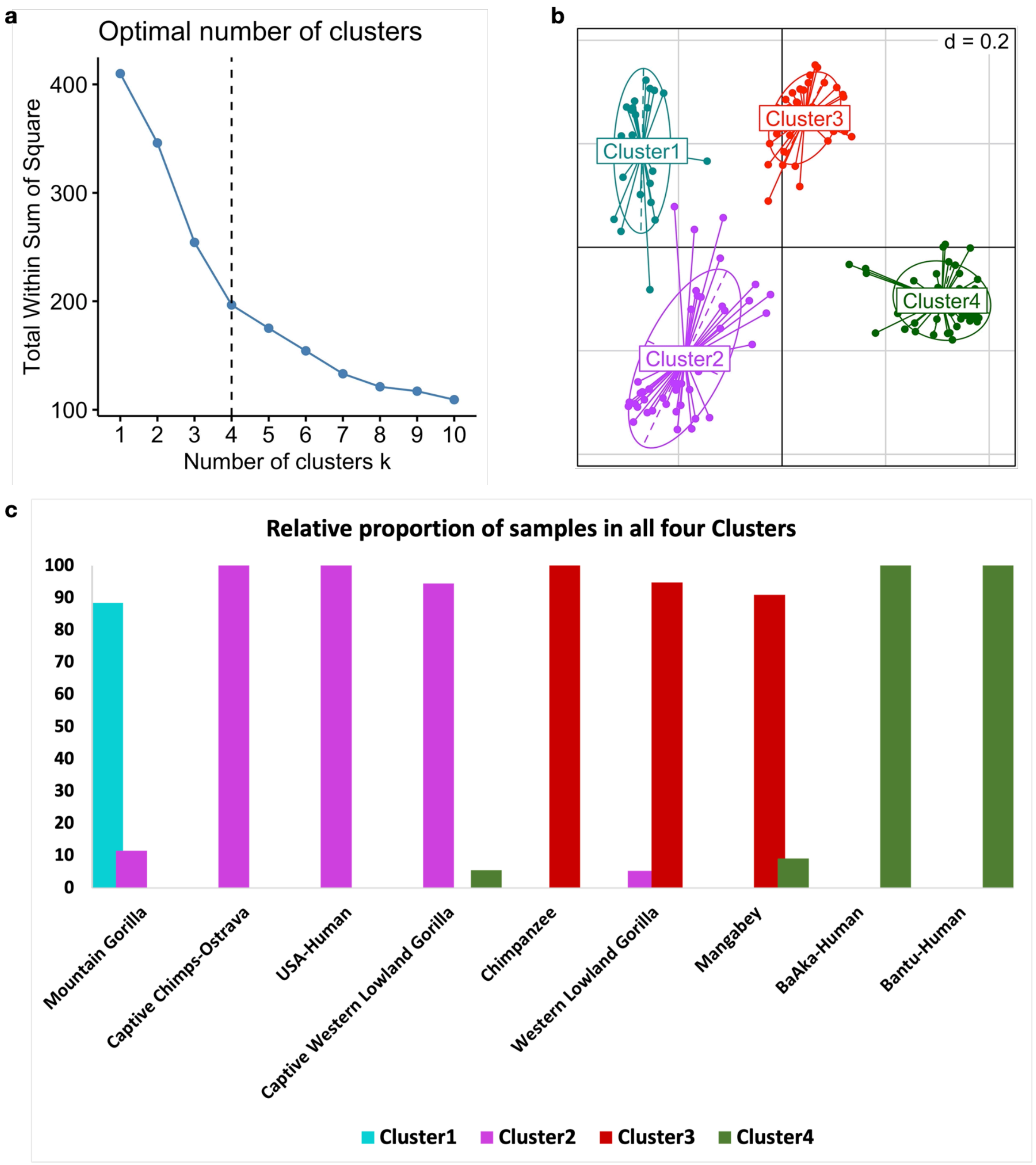

Supplementary Figure 2. Gut mycobiome community composition of different primates can be classified into four different clusters. (**a)** Partition around medoids (PAM) clustering analysis led to the identification of minimum four clusters based on reduction in sums of squares after k=4. (**b)** Bray-Curtis PCoA ordination showing the presence of four clusters classifying gut mycobiome community composition across different primate species. Each dot in the plot represents the fecal sample of a given primate and ellipses show the confidence intervals (95% standard errors) in multivariate space. (**c)** Proportion of individuals within each primate species classified into each of the four clusters shown in the bar plot.

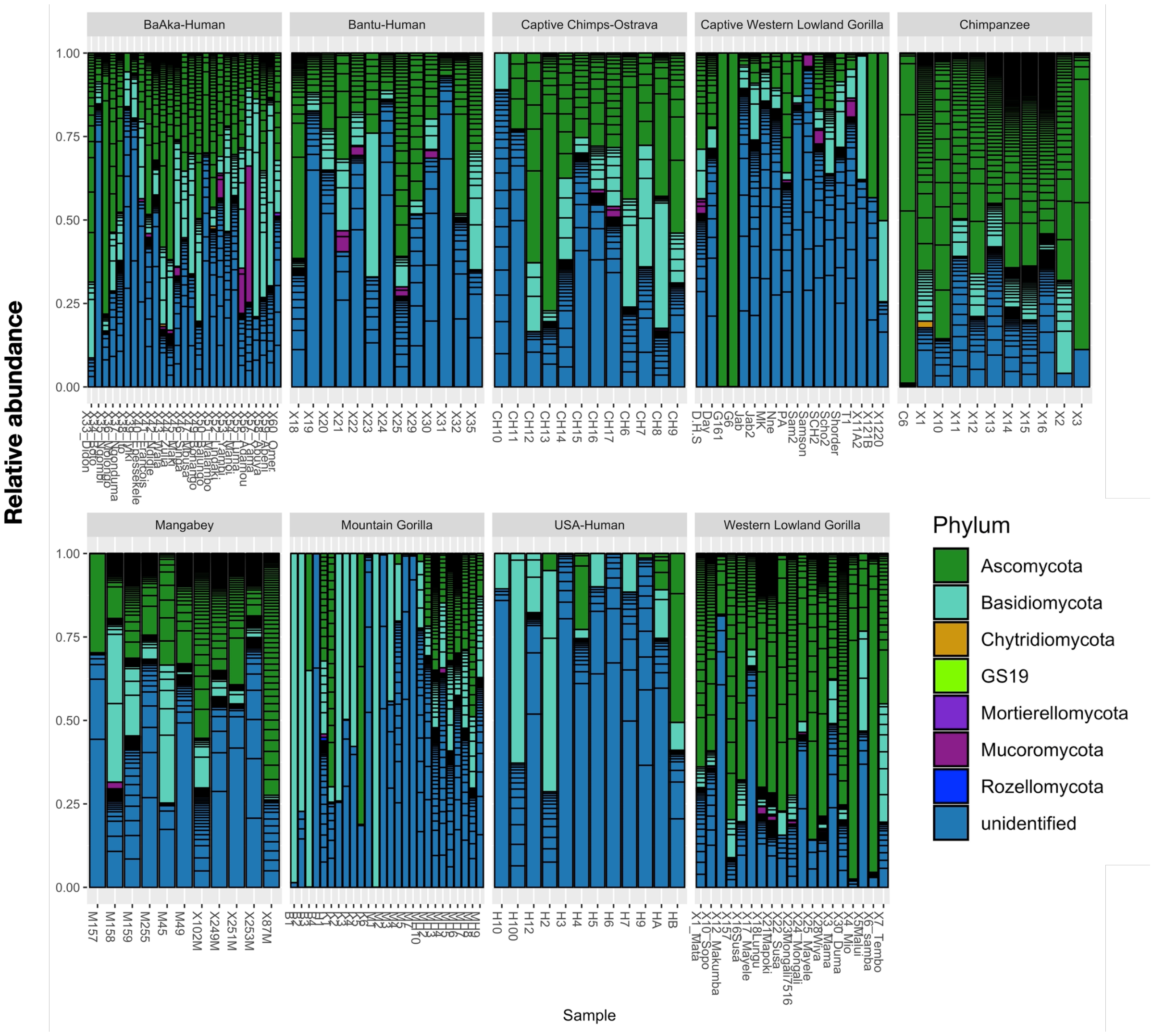

Supplementary Figure 3. Gut mycobiome community composition across different primate groups. Bar plots showing relative abundance of fungal phyla detected in each primate group.

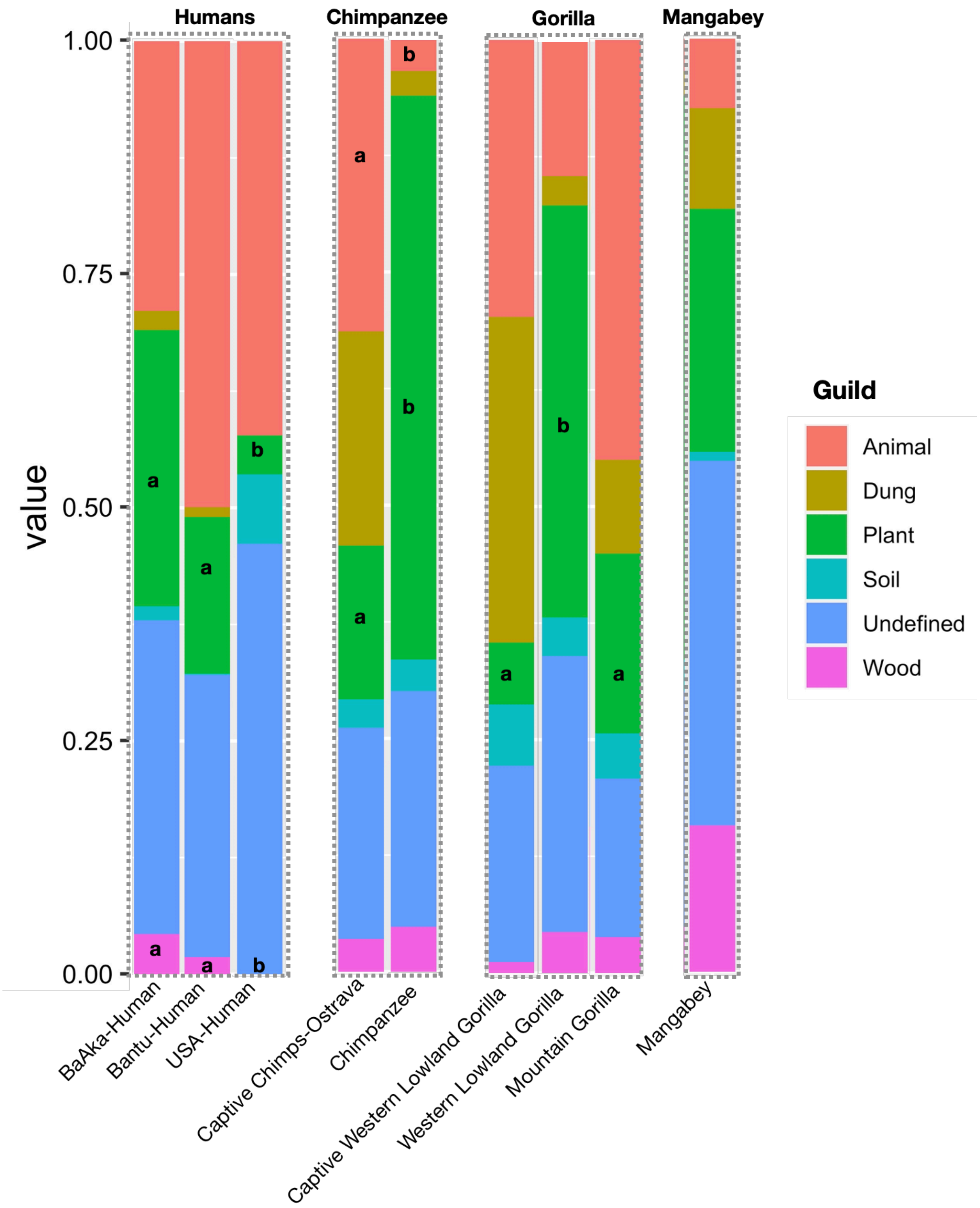

Supplementary Figure 4. Cumulative relative abundance of all fungal ASVs that can utilize similar types of resources (guild source). Relative distributions of guild source were detected from the FUNGuild assignments of fungal ASVs. Symbols highlight statistical differences (Kruskal-Wallis, $p < 0.05$) in the distribution of guild source within each human, chimpanzee and gorilla population.

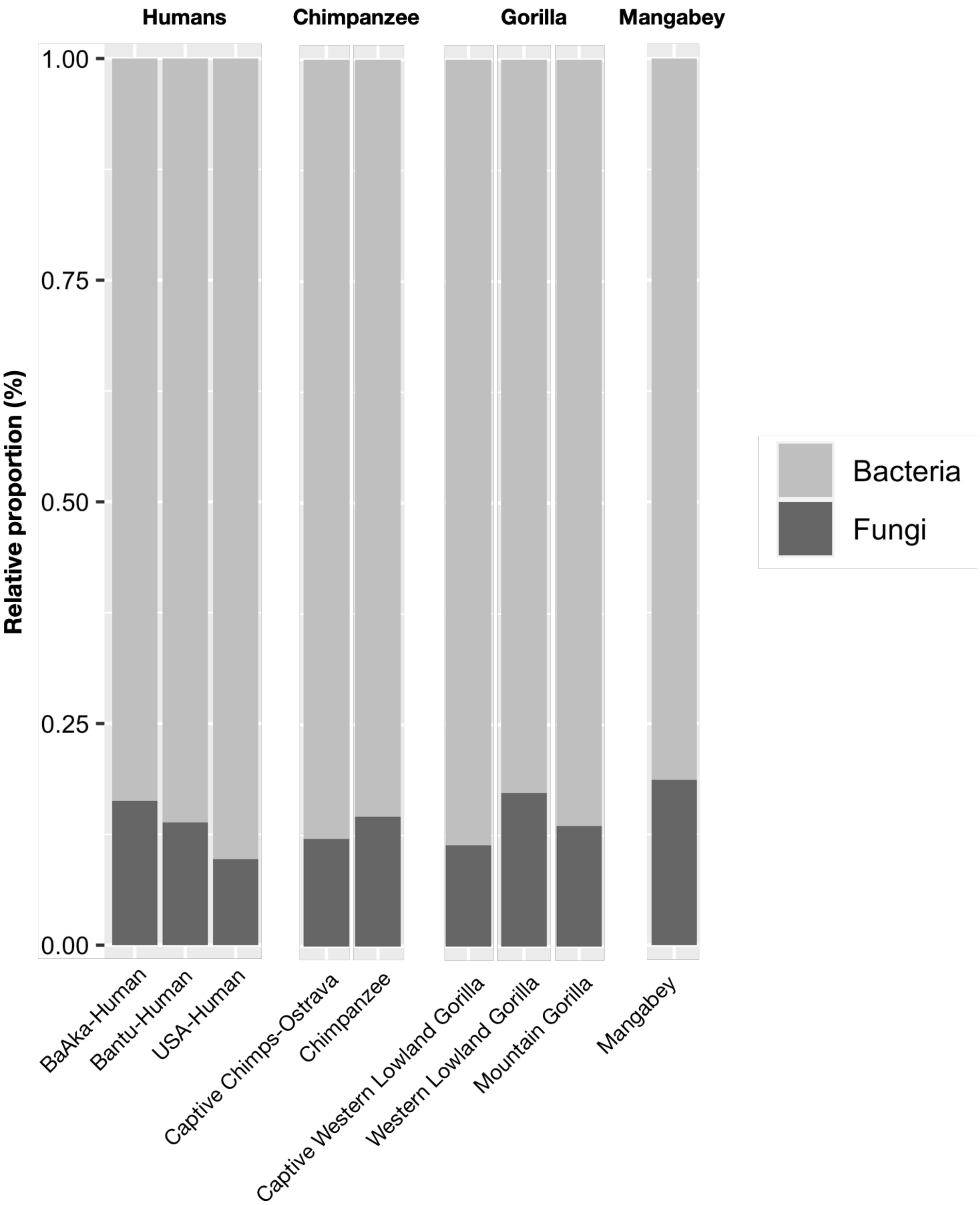

Supplementary Figure 5. Relative proportion of gut mycobiome and bacteriome among different primate groups. Centered log-ratio (CLR) transformation was used on bacterial and fungal count data separately before merging and assessing the relative proportions. Bacteriome composition is always higher (~4 times, ratio=4:1) than mycobiome irrespective of the primate host.

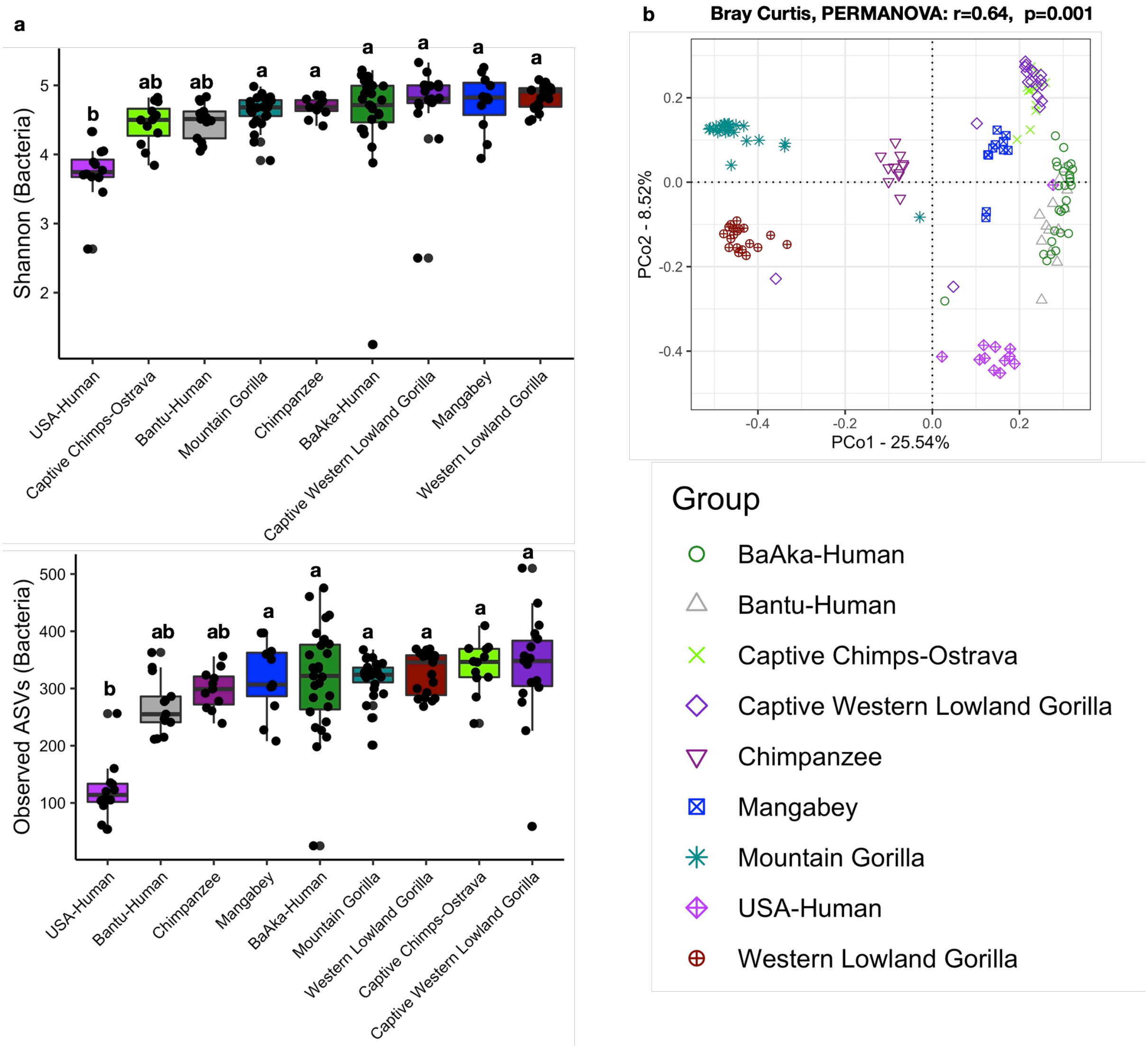

Supplementary Figure 6. Gut bacterial community composition differs based on subsistence strategies across different primates. **(a)** Alpha diversity analysis showing lower bacterial diversity and richness in western humans compared with other primate populations. **(b)** Principal coordinate analysis based on Bray-Curtis showing different bacterial community composition across different primate groups analyzed (PERMANOVA, R2=0.53, p=0.001, and R2=0.64, p=0.001). Each symbol represents the fungal composition, at the ASV level, in the fecal samples of an individual primate species.

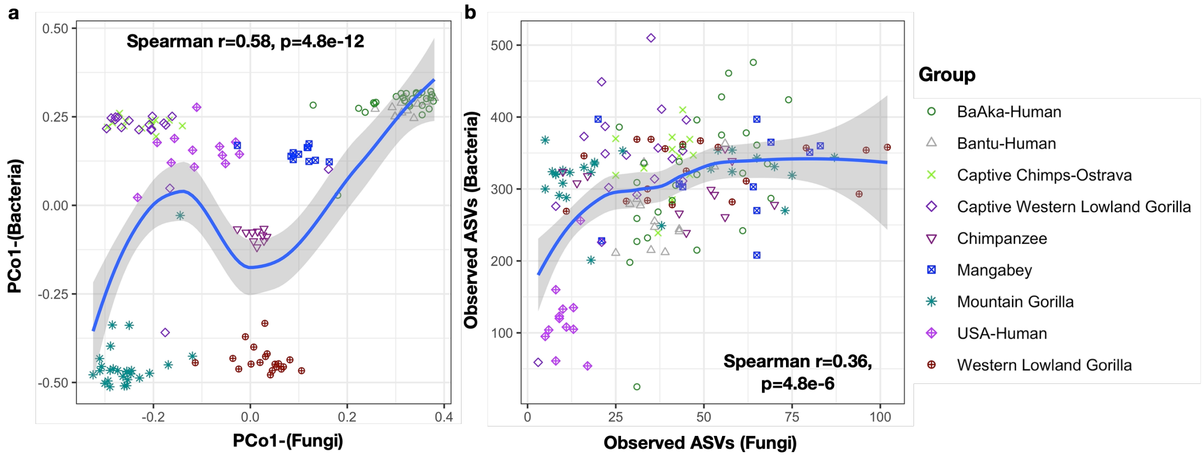

Supplementary Figure 7. Correspondence between gut bacteriome and mycobiome composition across different primates. **(a)** Correlation between ordination scores along PCo1 based on Bray-Curtis distances calculated on gut mycobiome and bacteriome composition, and (**b)** correlation between gut fungal and bacterial richness.

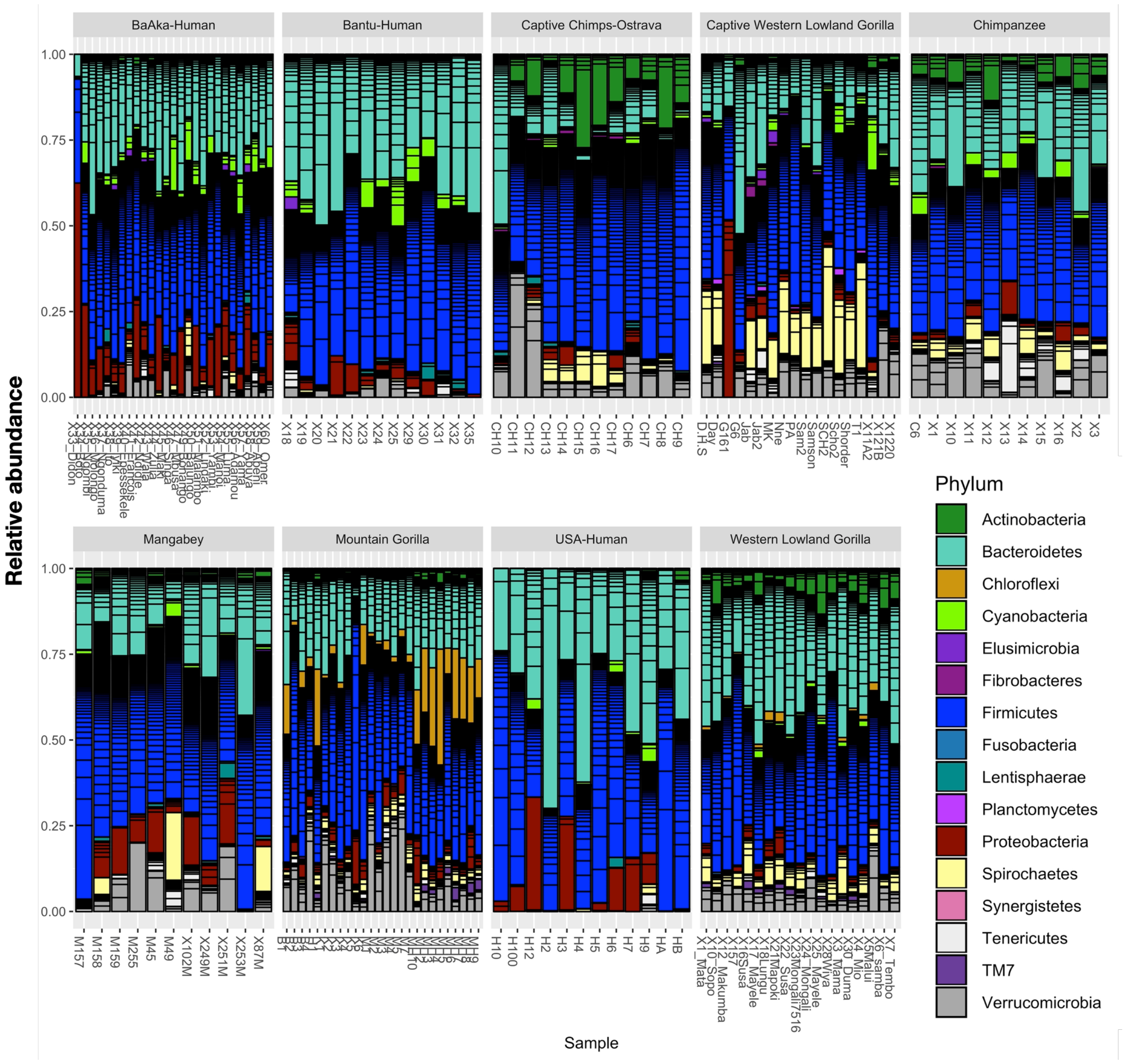

Supplementary Figure 8. Gut bacterial community composition across different primate groups. Bar plots showing relative abundance of bacterial phylums detected in each primate group.

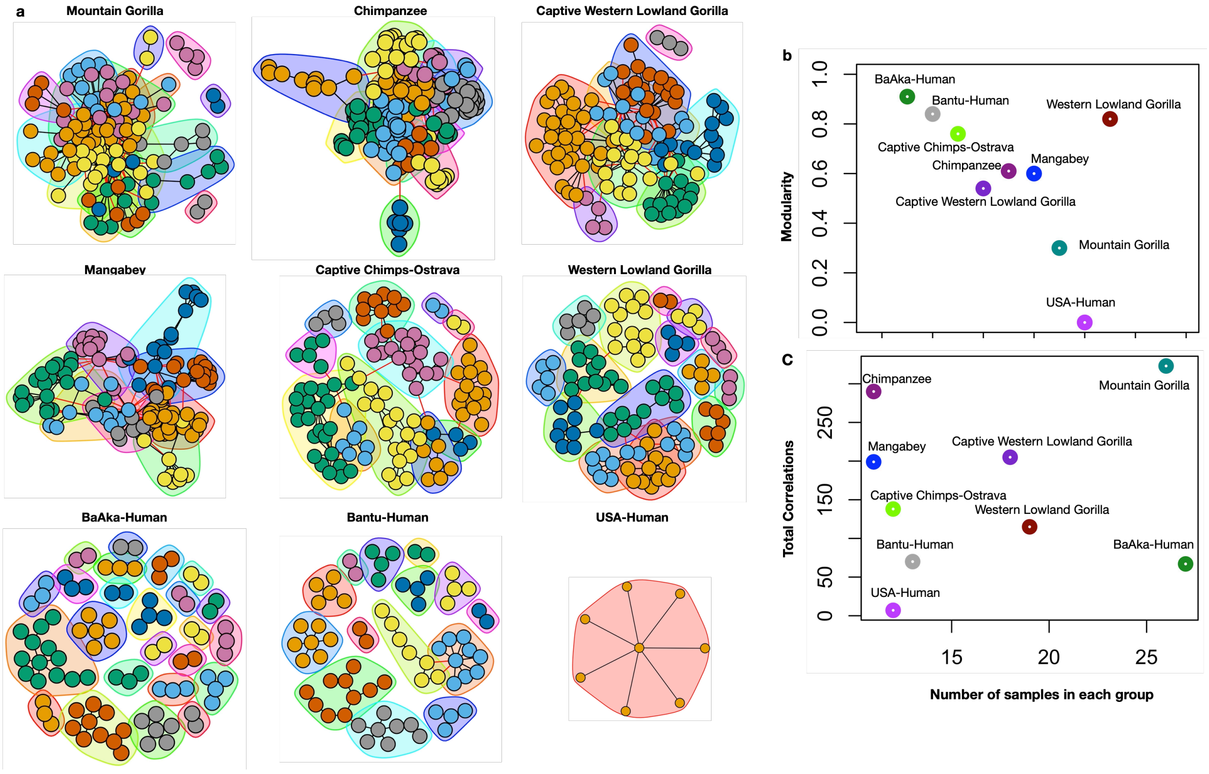

Supplementary Figure 9. Gut mycobiome-bacteriome co-occurrence network analysis across different primates. **(a)** Graphical representation of co-occurrence network modularity constructed using all significant correlations, **(b)** Network modularity of all primate groups where lower modularity results in dense connections (except of USA humans where only one node is connected). **(c)** association between number of samples and number of significant correlations between mycobiome and bacteriome obtained in each primate group.

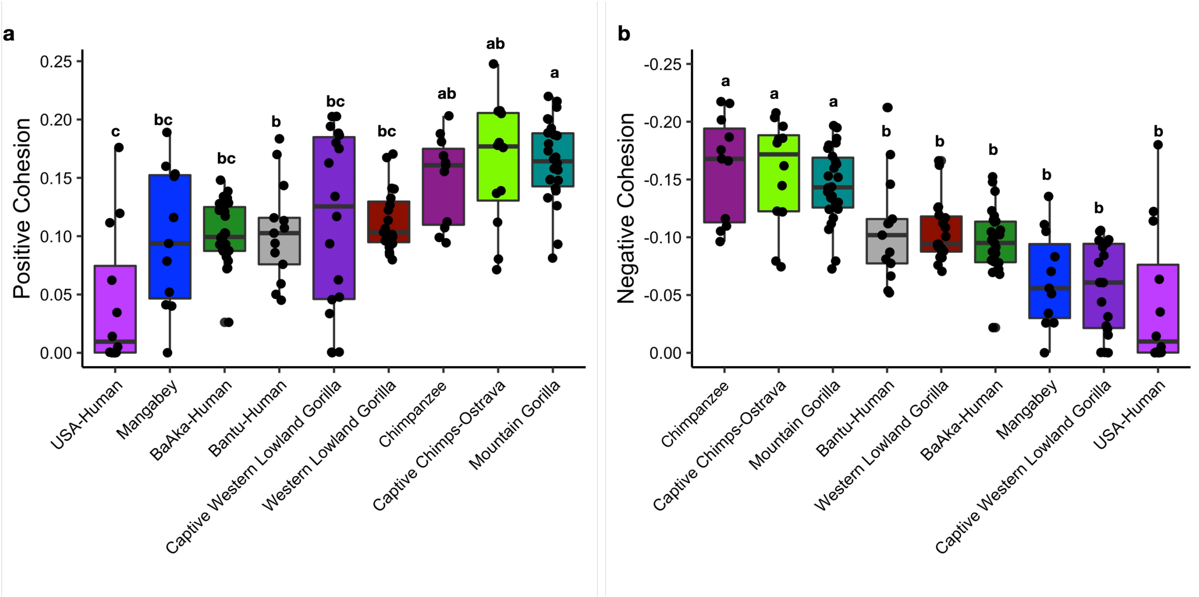

Supplementary Figure 10. Gut mycobiome-bacteriome co-occurrence network analysis across different primates. **(a)** positive cohesion **(b)** negative cohesion across different primate groups. Boxplots show inner quartiles and median values. Statistical significance was derived using Kruskal-Wallis multiple comparisons.

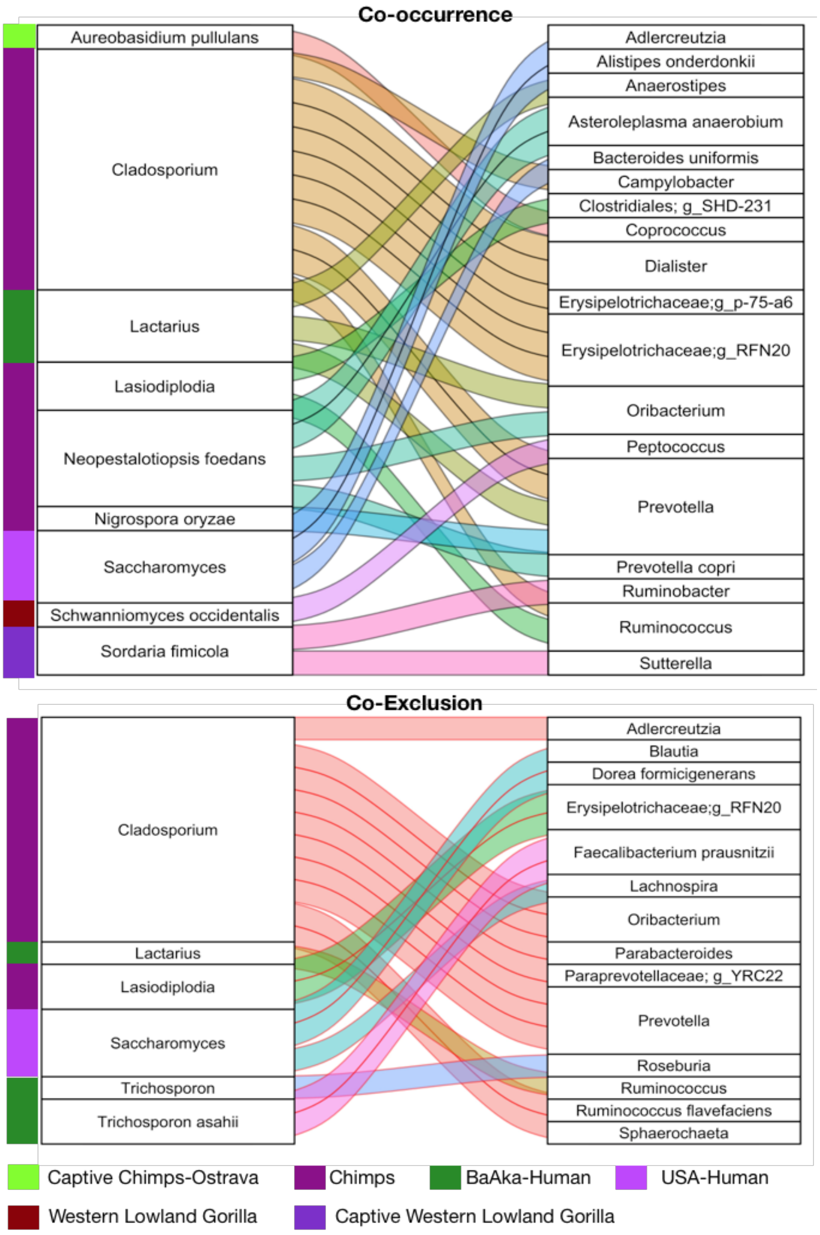

Supplementary Figure 11. Association between bacterial taxa and representative fungal genus of individual primate groups. Compositional correlations (co-occurrence and co-exclusion, r>0.6, q<0.01) were calculated between all significantly discriminating bacterial ASVs (species indicator analysis, p<0.01) and discriminant fungal genera.
