# Supplementary material for: The primate gut mycobiome-bacteriome interface is impacted by environmental and subsistence factors
Source: NPJ Biofilms Microbiomes. 2022 Mar 17;8:12. doi: 10.1038/s41522-022-00274-3 (PMC8930997; doi:10.1038/s41522-022-00274-3)
Supplement: Supplementary file 2 — Reporting Summary [file 41522_2022_274_MOESM2_ESM.pdf]

nature portfolio

Corresponding author(s): NPJBIOFILMS-01470R

Last updated by author(s): Jan 18, 2022

# Reporting Summary

Nature Portfolio wishes to improve the reproducibility of the work that we publish. This form provides structure for consistency and transparency in reporting. For further information on Nature Portfolio policies, see our Editorial Policies and the Editorial Policy Checklist.

## Statistics

For all statistical analyses, confirm that the following items are present in the figure legend, table legend, main text, or Methods section.

| n/a | Confirmed | |
|---|---|---|
| ☐ | ☒ | The exact sample size (*n*) for each experimental group/condition, given as a discrete number and unit of measurement |
| ☐ | ☒ | A statement on whether measurements were taken from distinct samples or whether the same sample was measured repeatedly |
| ☐ | ☒ | The statistical test(s) used AND whether they are one- or two-sided<br>*Only common tests should be described solely by name; describe more complex techniques in the Methods section.* |
| ☐ | ☒ | A description of all covariates tested |
| ☐ | ☒ | A description of any assumptions or corrections, such as tests of normality and adjustment for multiple comparisons |
| ☐ | ☒ | A full description of the statistical parameters including central tendency (e.g. means) or other basic estimates (e.g. regression coefficient) AND variation (e.g. standard deviation) or associated estimates of uncertainty (e.g. confidence intervals) |
| ☐ | ☒ | For null hypothesis testing, the test statistic (e.g. *F*, *t*, *r*) with confidence intervals, effect sizes, degrees of freedom and *P* value noted<br>*Give P values as exact values whenever suitable.* |
| ☒ | ☐ | For Bayesian analysis, information on the choice of priors and Markov chain Monte Carlo settings |
| ☒ | ☐ | For hierarchical and complex designs, identification of the appropriate level for tests and full reporting of outcomes |
| ☐ | ☒ | Estimates of effect sizes (e.g. Cohen's *d*, Pearson's *r*), indicating how they were calculated |

*Our web collection on statistics for biologists contains articles on many of the points above.*

## Software and code

Policy information about availability of computer code

| | |
|---|---|
| Data collection | GreenGenes, and UNITE database |
| Data analysis | Cutadapt, Fastx_toolkit, Qiime2/2020.4, Dada2, FUNGuild, CCREPE, Cytoscape_v3.8.2, Igraph, Vegan, Ape, Phyloseq, SPIEC-EASI, Labdsv, hclust, kruskalmc, pgirmess, NMF, and ggplot2 |

For manuscripts utilizing custom algorithms or software that are central to the research but not yet described in published literature, software must be made available to editors and reviewers. We strongly encourage code deposition in a community repository (e.g. GitHub). See the Nature Portfolio guidelines for submitting code & software for further information.

## Data

Policy information about availability of data

All manuscripts must include a data availability statement. This statement should provide the following information, where applicable:

- Accession codes, unique identifiers, or web links for publicly available datasets
- A description of any restrictions on data availability
- For clinical datasets or third party data, please ensure that the statement adheres to our policy

16S rRNA and ITS2 sequences generated in this study have been deposited in the NCBI SRA under the BioProject identifier (ID) code: PRJNA686661

# Field-specific reporting

Please select the one below that is the best fit for your research. If you are not sure, read the appropriate sections before making your selection.

☐ Life sciences ☐ Behavioural & social sciences ☒ Ecological, evolutionary & environmental sciences

For a reference copy of the document with all sections, see nature.com/documents/nr-reporting-summary-flat.pdf

# Ecological, evolutionary & environmental sciences study design

All studies must disclose on these points even when the disclosure is negative.

| | |
|---|---|
| Study description | This study focuses on characterizing variation in mycobiome composition and mycobiome - bacteriome interactions across different primate species, including effect o lifestyle (captivity or westernization vs free range or traditional subsistence) |
| Research sample | The groups targeted were: western lowland gorillas (Gorilla gorilla gorilla, n=19), agile mangabeys (Cercocebus agilis, n=11), BaAka hunter-gatherers (n=27), Bantu agriculturalists (n=13), eastern chimpanzees (Pan troglodytes schweinfurthii, n=11), captive western lowland gorillas (n=18), captive chimpanzees (n=12), mountain gorillas (Gorilla beringei beringei, n=26), and a US human cohort (n=12). We aimed to sample primates under different subsistence strategies including both apes and monkeys. |
| Sampling strategy | Sample size had to be based on the availability of subjects within each filed location- we tried to collect as many subjects we could for a given primate group, aiming for a minimum of 10 samples per group, which we have demonstrated is sufficient to capture inter/ intra-species differences in microbiome composition among primates. |
| Data collection | About 1 gr of fecal sample, taken from the inner core of feces, avoiding the exterior and within 1-2 hours after voiding, was collected and then stored in 5 ml tubes containing RNAlater (Qiagen, Germany, 1g of fecal sample in 2 volumes of solution). Then, the solution was mixed thoroughly to homogenize the sample. Depending on field/site infrastructure, samples remained at room temperature from 24 hours for a maximum of three weeks before storage at -20oC until DNA extraction. Samples and data were collected/ generated by all authors in the study. |
| Timing and spatial scale | Fecal samples were collected from all primate groups in a span of three years, from 2012 and 2016; starting with G..g. beringiei and C. agiliis, followed by the captive primates and US humans. Frequency of collection o wild samples depended on visits to field sites. DNA was extracted between 2013 and 2017 and sequencing was performed in 2018. Data analyses were performed from 2019-2020 |
| Data exclusions | No samples or data were excluded. |
| Reproducibility | We possess a comprehensive database of 16S rRNA data obtained from fecal samples of diverse primate species, based on 454 pyrosequencing and metagenomic sequencing, as such, reproducibility of these data were confirmed by contrasting with our previously generated datasets. Reproducibility of the ITS2 data is yet to be confirmed as this is the first dataset of ITS2 data across these species of non human primates, and mock fungal communities were not available. Regardless, fungal data corresponds to what has been reported in other mammalian and human ITS2 sequencing projects. |
| Randomization | Randomization is not relevant in this study. None of the subjects were allocated to any dietary treatment |
| Blinding | Blinding is not relevant in this study. None of the subjects were allocated to any dietary treatment |

Did the study involve field work? ☒ Yes ☐ No

## Field work, collection and transport

| | |
|---|---|
| Field conditions | In field conditions, samples were collected throughout the year including seasons of low precipitation (G.g.beringei) or rainy seasons (G.g.gorilla, P. tr. schweinfurthii and C.agilis). |
| Location | -Samples from four social groups of western lowland gorillas (Gorilla gorilla gorilla, n=19), one group of agile mangabeys (Cercocebus agilis, n=11), BaAka hunter-gatherers (n=27), and Bantu agriculturalists (n=13) were collected at the Dzanga Sangha Protected Areas, Central African Republic (2o50'N,16o28'E).<br>-Samples from one group of eastern chimpanzees (Pan troglodytes schweinfurthii, n=11) were collected in Bulindi, Uganda (1.4663° N, 31.4442° E)<br>-Samples from captive western lowland gorillas (n=18) and US humans were collected at Como zoo in St Paul Minnesota, USA (44.9537° N, 93.0900° W)<br>-Samples from captive chimpanzees were collected at Ostrava (n=12) zoos in the Czech Republic (49.8209° N, 18.2625° E)<br>Samples from mountain gorillas (Gorilla beringei beringei, n=26) from four social groups were collected at Bwindi Impenetrable National Park, Uganda (0o53'–1o08'S, 29o35'—29o50'E). |
| Access & import/export | Samples from wild western lowland Gorillas, Mangabeys and traditional human populations from the Central African Republic were collected with approval by the Ministre de l'Education Nationale, de l'Alphabetisation, de l'Enseignement Superieur, and de la Recherche (Central African Republic) while samples from wild mountain gorillas were collected with approval from The Uganda Wildlife Authority and the Uganda National Council for Science and Technology. Samples from wild chimpanzees were collected with approval from Makerere University in Uganda, protocol number HDREC421 and from Oxford Brooks University in the UK, protocol number UREC-160989. Samples from captive apes from St.Paul, MN, USA and Europe were collected under protocol ID 2003-37934A |

| | |
|---|---|
| | granted by the University of Minnesota, Twin Cities. Samples from US humans were collected with approval by the University of Minnesota, Twin Cities, and its Institutional review board (IRB) for the protection of human subjects, protocol number STUDY00004208 |
| Disturbance | All samples were collected non-invasively and within 1-2 hours after defecation; thus no significant disturbance was imposed on the animals |

# Reporting for specific materials, systems and methods

We require information from authors about some types of materials, experimental systems and methods used in many studies. Here, indicate whether each material, system or method listed is relevant to your study. If you are not sure if a list item applies to your research, read the appropriate section before selecting a response.

## Materials & experimental systems

| n/a | Involved in the study |
|---|---|
| ☒ | ☐ Antibodies |
| ☒ | ☐ Eukaryotic cell lines |
| ☒ | ☐ Palaeontology and archaeology |
| ☐ | ☒ Animals and other organisms |
| ☐ | ☒ Human research participants |
| ☒ | ☐ Clinical data |
| ☒ | ☐ Dual use research of concern |

## Methods

| n/a | Involved in the study |
|---|---|
| ☒ | ☐ ChIP-seq |
| ☒ | ☐ Flow cytometry |
| ☒ | ☐ MRI-based neuroimaging |

# Animals and other organisms

Policy information about studies involving animals; ARRIVE guidelines recommended for reporting animal research

| | |
|---|---|
| Laboratory animals | Study did not involve laboratory animals |
| Wild animals | Fecal samples from wild animals were collected in the field. |
| Field-collected samples | All samples were collected in RNAlater (1g of fecal sample in 2 volumes of solution) in the field before storage at -80°C until processing |
| Ethics oversight | Nonhuman primate samples: Samples from wild western lowland Gorillas and Mangabeys from the Central African Republic were collected with approval by the Ministre de l'Education Nationale, de l'Alphabetisation, de l'Enseignement Superieur, and de la Recherche (Central African Republic) while samples from wild mountain gorillas were collected with approval from The Uganda Wildlife Authority and the Uganda National Council for Science and Technology. Samples from wild chimpanzees were collected with approval from Makerere University in Uganda, protocol number HDREC421 and from Oxford Brooks University in the UK, protocol number UREC-160989. Samples from captive apes from St.Paul, MN, USA and Europe were collected under protocol ID 2003-37934A granted by the University of Minnesota, Twin Cities. |

Note that full information on the approval of the study protocol must also be provided in the manuscript.

# Human research participants

Policy information about studies involving human research participants

| | |
|---|---|
| Population characteristics | Male and female healthy participants from US humans and from Central African Republic were recruited to understand the gut mycobiome and bacteriome differences due to lifestyle differences. |
| Recruitment | BaAka hunter-gatherers (n=27), and Bantu agriculturalists (n=13) were collected at the Dzanga Sangha Protected Areas, Central African Republic. Samples from the US human population (n=12) were collected from healthy subjects in St. Paul MN. |
| Ethics oversight | Human samples: Approval to collect samples from US humans was granted by the University of Minnesota, Twin Cities, and its Institutional review board (IRB) for the protection of human subjects, protocol number STUDY00004208. All work carried out with hunter-gatherers and agriculturalists from the Central African Republic, including sample collection, was approved according to the rules and regulations from the Ministre de l'Education Nationale, de l'Alphabetisation, de l'Enseignement Superieur, and de la Recherche (Central African Republic), as well as the IRB for the protection of human subjects from the University of Illinois at Urbana-Champaign (permit number 13045). All participants provided written informed consent to take part in the study. Samples from US humans were collected with approval by the University of Minnesota, Twin Cities, and its Institutional review board (IRB) for the protection of human subjects, protocol number STUDY00004208 |

Note that full information on the approval of the study protocol must also be provided in the manuscript.
